# Combining GWAS and RNA-Seq Approaches for Detection of the Causal Mutation for Hereditary Junctional Epidermolysis Bullosa in Sheep

**DOI:** 10.1371/journal.pone.0126416

**Published:** 2015-05-08

**Authors:** Aroa Suárez-Vega, Beatriz Gutiérrez-Gil, Julio Benavides, Valentín Perez, Gwenola Tosser-Klopp, Christophe Klopp, Stephen J. Keennel, Juan José Arranz

**Affiliations:** 1 Departamento de Producción Animal, Facultad de Veterinaria, Universidad de León, Campus de Vegazana s/n, 24071, León, Spain; 2 Departamento de Sanidad Animal (Anatomía Patológica), Instituto de Ganadería de Montaña (CSIC-ULE), Facultad de Veterinaria, Universidad de León, Campus de Vegazana s/n, 24071, León, Spain; 3 INRA, UMR1388 GenPhySE (Génétique, Physiologie et Systèmes d’Elevage), F-31326, Castanet-Tolosan, France; 4 Université de Toulouse, INP, ENSAT, GenPhySE (Génétique, Physiologie et Systèmes d’Elevage), F-31326, Castanet-Tolosan, France; 5 Université de Toulouse, INP, ENVT, GenPhySE (Génétique, Physiologie et Systèmes d’Elevage), F-31076, Toulouse, France; 6 INRA, Plateforme bioinformatique Toulouse Midi-Pyrénées, UR875 Biométrie et Intelligence Artificielle, BP 52627, 31326, Castanet-Tolosan Cedex, France; 7 Graduate School of Medicine, University of Tennessee, Knoxville, 37920, Tennessee, United States of America; University of Vienna, AUSTRIA

## Abstract

In this study, we demonstrate the use of a genome-wide association mapping together with RNA-seq in a reduced number of samples, as an efficient approach to detect the causal mutation for a Mendelian disease. Junctional epidermolysis bullosa is a recessive genodermatosis that manifests with neonatal mechanical fragility of the skin, blistering confined to the lamina lucida of the basement membrane and severe alteration of the hemidesmosomal junctions. In Spanish Churra sheep, junctional epidermolysis bullosa (JEB) has been detected in two commercial flocks. The JEB locus was mapped to *Ovis aries* chromosome 11 by GWAS and subsequently fine-mapped to an 868-kb homozygous segment using the identical-by-descent method. The *ITGB4*, which is located within this region, was identified as the best positional and functional candidate gene. The RNA-seq variant analysis enabled us to discover a 4-bp deletion within exon 33 of the *ITGB4* gene (*c*.*4412_4415del*). The *c*.*4412_4415del* mutation causes a frameshift resulting in a premature stop codon at position 1472 of the integrin β4 protein. A functional analysis of this deletion revealed decreased levels of mRNA in JEB skin samples and the absence of integrin β4 labeling in immunohistochemical assays. Genotyping of *c*.*4412_4415del* showed perfect concordance with the recessive mode of the disease phenotype. Selection against this causal mutation will now be used to solve the problem of JEB in flocks of Churra sheep. Furthermore, the identification of the *ITGB4* mutation means that affected sheep can be used as a large mammal animal model for the human form of epidermolysis bullosa with aplasia cutis. Our approach evidences that RNA-seq offers cost-effective alternative to identify variants in the species in which high resolution exome-sequencing is not straightforward.

## Introduction

Epidermolysis bullosa (EB) is a clinically and genetically heterogeneous group of inherited mechanobullous disorders caused by mutations in several structural skin proteins. EB is characterized by structural and mechanical fragility of the skin, leading to recurrent blistering and erosions that impair the life of EB affected individuals [1]. Following the latest classification [2], there are four major types of EB: EB simplex (EBS), junctional EB (JEB), dystrophic EB (DEB) and Kindler. This classification has been developed based on the level of blisters and the location of the aberrant proteins involved.

JEB encompasses all of the subtypes of EB that have mechanical fragility and blistering confined to the lamina lucida of the basement membrane and severe alteration of the hemidesmosomal junctions [1]. The hemidesmosomes are multi-protein complexes that play a critical role in the stable association of basal epithelial cells to the underlying basement membrane in stratified and pseudostratified epithelia [3]. Mutations in the genes encoding hemidesmosomal proteins and functionally associated structures have been shown to result in different subtypes of JEB [4, 5]. JEB occurs in various mammalian species [6–13]. In sheep, a Herlitz subtype of JEB has been described in German Black-headed Mutton sheep [14–16].

**Fig 1:**
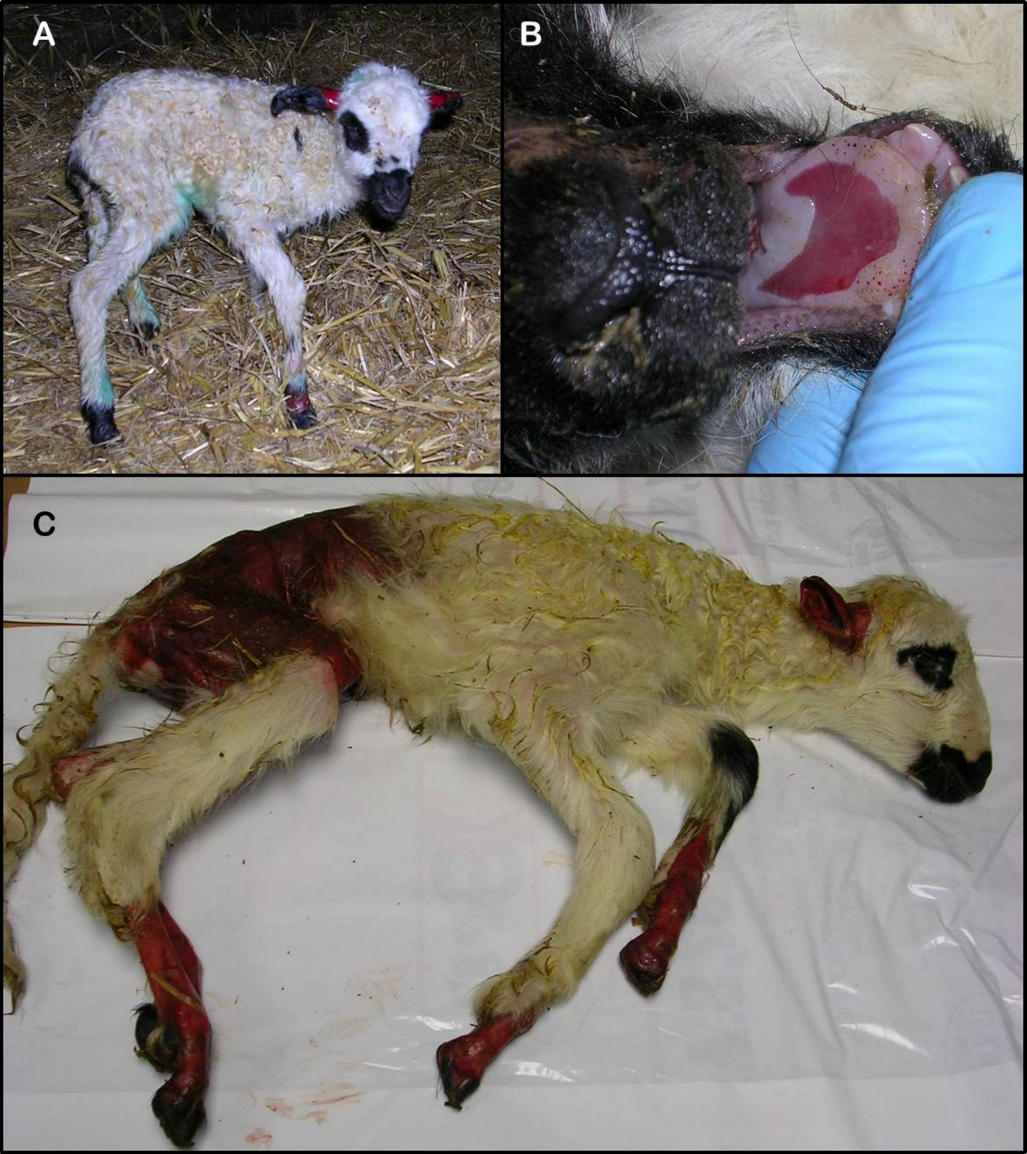
Clinical features of JEB in Churra sheep. (A) Affected lamb showing skin lesions at the ears and limbs. (B) Erosions in the tongue. (C) Affected lamb showing severe skin lesions that involve the ears, limbs and caudal part of the body.

In Spanish Churra sheep, JEB has been detected in two commercial flocks belonging to the National Churra Sheep Breeders’ Association (ANCHE). The affected animals show erosions and ulcers of the skin and mucous membranes (Fig 1). Initial analyses showed that the JEB in Churra sheep is a recessive autosomal inherited defect. Microscopically, JEB with congenital absence of skin have been diagnosed. In humans, the concurrence of JEB and congenital absence of skin is described in a subtype of JEB known as JEB with pyloric atresia (JEB-PA) [2]. Pyloric atresia has not been identified in the affected Churra lambs. Because of the clinical and molecular heterogeneity of EB, there are some rare cases of JEB-PA-affected humans who did not show pyloric atresia [17]. JEB-PA is related to mutations in the *ITGA6* and *ITGB4* genes [18–22].

In recent years, genome-wide mapping strategies have proven to be effective approaches for the identification of defective genes underlying diseases with Mendelian inheritance patterns [15, 23, 24]. The methods to identify the causal mutation in a Mendelian disorder generally involve the detection of a disease locus followed by candidate gene sequencing. Recently, Next Generation Sequencing (NGS) technologies have enabled high-throughput functional genomic research to be performed [25]. High-throughput sequencing of RNA (RNA-seq) has been developed to identify and quantify the gene expression in different tissues [26, 27]. RNA-seq also offers new opportunities for efficient and cost-effective detection of transcriptome variants (SNPs and short indels) in different tissues and species [28–30]. In humans, recent studies have highlighted the advantages of using NGS in EB diagnostics [31, 32].

In the present study, our aim was to find the causative mutation underlying JEB in Spanish Churra sheep. For this goal, a SNP array was used to map the causative region of JEB, and then, the disease-causing mutation was identified via an RNA-seq variant analysis. This study demonstrates the effectiveness of combining both approaches to map and identify the causal mutations in recessive Mendelian diseases.

## Results

### Mapping the causative gene for JEB

**Fig 2:**
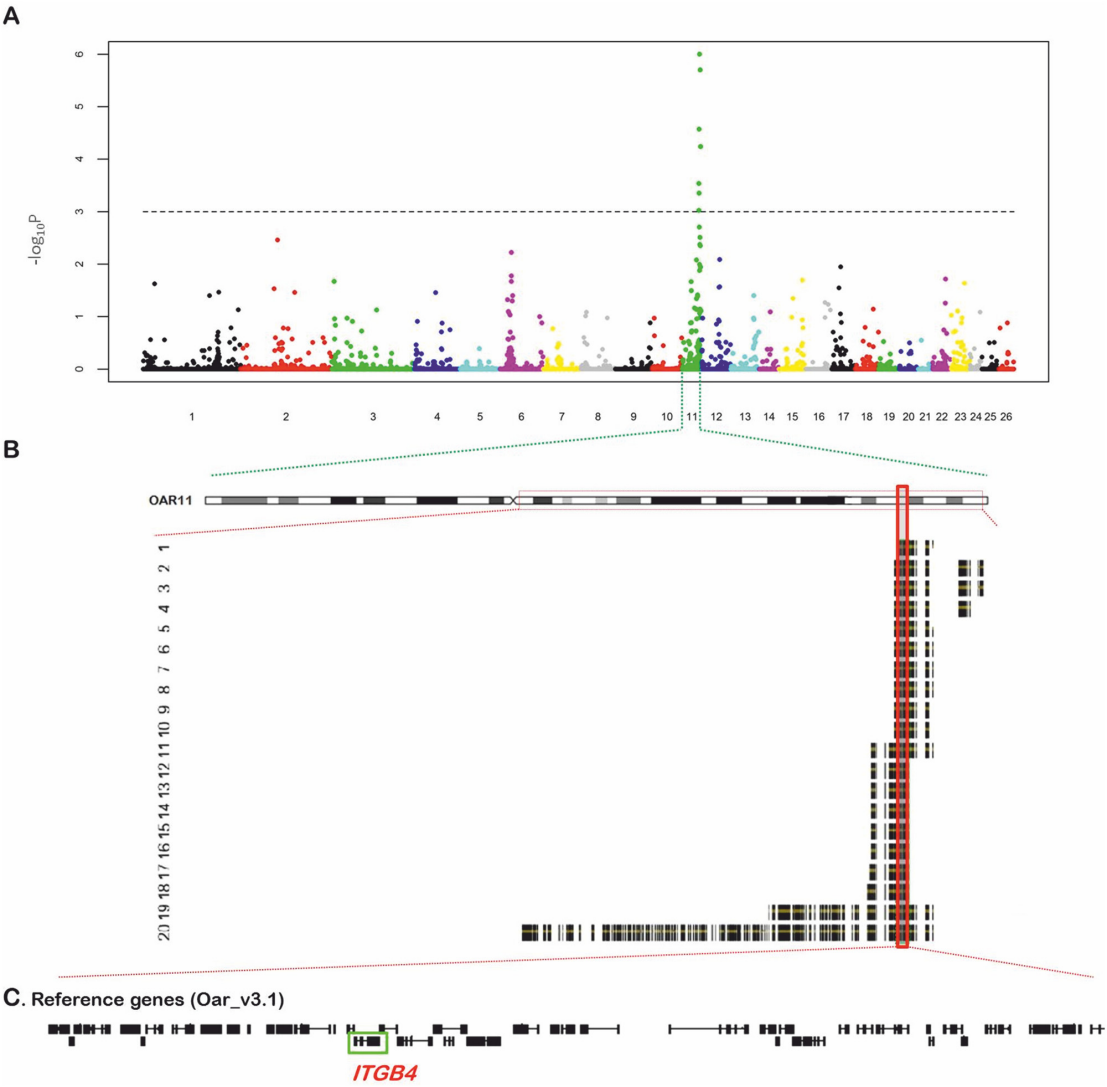
Workflow chart for the discovery of the mutant locus of inherited JEB in Churra sheep. (A) Manhattan plot that shows the results of the case-control GWA analysis performed. The X-axis shows the positions of the SNPs analyzed across the 26 ovine autosomes; different colors represent each different chromosome. The Y-axis represents the −log10P values obtained after performing 1 million permutations using PLINK. The most significant associated SNP is located at position 54,939,690 bp on OAR11. (B) IBD analysis on OAR11. The analysis of SNPs genotypes from affected lambs shows an extended homozygous region on chromosome 11. All twenty affected lambs shared overlapping homozygous blocks (black and yellow blocks, which represent the genes within the homozygous block of each animal). The consensus haplotype block (indicated by a solid red box) that contains the putative causal mutation expands a region of 868-kb, from 54,632,309–55,500,100 bp on OAR11. (C) Gene content of the 868-kb interval shared by the JEB affected.

Approximately 44,800 evenly spaced SNPs were genotyped in 20 JEB-affected lambs and 28 related unaffected and 48 unrelated control sheep. A significant genome-wide association between the SNP genotypes and JEB was shown for sheep chromosome 11 (OAR11) (Fig 2A). The marker rs410387229, located at position OAR11:54,939,690 bp of the OARv3.1 sheep genome assembly, showed a genome-wide corrected p-value of 0.00001. There were three additional SNP markers on OAR11, rs418150485, rs425824336 and rs412661420, located at positions 56,897,973, 54,003,142 and 58,681,220 bp, respectively, with corrected p-values < 0.0001.

The location of the JEB locus was refined using the identical-by-descent (IBD) mapping method. The IBD analysis narrowed the defective region to an 868-kb homozygous segment, from 54,632,309–55,500,100 bp on OAR11, in all 20 affected lambs (Fig 2B). The consensus region contains 16 consecutive SNP loci. Regarding the chromosomal location of the genes related to JEB in humans (OMIM #226730: *ITGB4*,*ITGA6*), the *ITGB4* gene was identified as a promising positional and functional candidate, as it is located at 54,848,050–54,874,496 on OAR11, which is within the identified block of homozygosis common to all of the cases (Fig 2C). The *ITGB4* gene codes for integrin β4 protein. In stratified epithelia, integrin α6β4 is located in specialized epidermal cell-basement membrane adhesion structures, called hemidesmosomes [33].

### Ovine ITGB4 gene sequence and annotation

Because of its association with human JEB [17, 22, 34], the *ITGB4* gene was identified as the most promising candidate. Our first aim was to identify the causal mutation via Sanger sequencing of the *ITGB4* gene in four JEB cases and four unrelated controls. Through the Sanger approach for genomic sequencing, we were able to obtain 84% (29,936 bp) of the complete genomic sequence of the *ITGB4* gene (ENSOARG00000009764), in the four cases and four controls. By comparing the sequences of the unaffected and the JEB-affected lambs, we identified eight single nucleotide polymorphisms (SNPs) that had have different genotypes in the cases (*g*.*54857417T>G*. *g*.*54857293C>T*, *g*.*54856642A>G*, *g*.*54853969C>G*, *g*.*5485255C>A*, *g*.*54851357C>T*, *g*.*54850814C>T* and *g*.*54848707G>A*). All of the identified SNPs were located in non-coding *ITGB4* regions. A search for the SNPs in the International Sheep Genomic Consortium (ISGC) database (https://data.csiro.au/dap/landingpage?execution=e1s2) allowed us to discard these mutations as causal mutations, as all of them were already published on healthy sheep from other breeds.

Because of the difficulties in performing Sanger DNA sequencing of the whole gene using the *ITGB4* reference sequence available in Ensembl (ENSOARG00000009764), we isolated the *ITGB4* sequence from three ovine BAC clones (CH243-344E15, CH243-306J7 and CH243-504K6) [35]. The BAC clone sequencing allowed us to obtain the complete genomic sequence of *ITGB4* (GenBank: KP025765). Pairwise alignment between the sheep *ITGB4* reference obtained via BAC clone sequencing and the *ITGB4* reference available in Ensembl (ENSOARG00000009764) revealed an identity of 83.1% between the two sequences and a gap percentage of 16.6%. Excluding the 5’- and 3’UTR regions that were not available in the Ensembl sequence, 14 gaps, ranging from 2 to 1119 bp, were detected (S1 Fig).

In parallel to the BAC clone sequencing, an RNA-seq analysis was conducted. Sequencing of the cDNA libraries from the RNA skin samples from one JEB case and one control generated a total of 69.31-million paired-end reads, with an average of 34.65-million paired-end reads for each sample. The aligner STAR [36] was used to map the paired-end reads from each sample to the ovine reference genome sequence (Oar_v3.1). Approximately 83.54% of the reads (85.69% in the case and 81.39% in the control) were mapped to a unique location in the ovine v3.1 genome. The combination of the *ITGB4* sequence obtained from the BAC clone with the RNA-seq data from the control allowed us to predict the complete CDS and the integrin β4 protein sequence. To determine the gene structure of the *ITGB4* sequence, Augustus software was used [37, 38]. In total, of 5360 reads that were overlapping the *ITGB4* region in the control sample were used to generate a hints file for Augustus. A complete CDS of 5256 bp was found. This transcript is distributed into 38 exons. The sheep *ITGB4* CDS sequence encodes a predicted protein of 1751 amino acids. The BLAST tool (http://blast.ncbi.nlm.nih.gov/Blast.cgi) was used to compare the predicted amino acid sequence from the ovine *ITGB4* gene with several available sequences, including the cow (DAA18168.1), camel (EQB77252.1) human (aai43743.1) and mouse (NP_598424.2) sequences. The predicted ovine integrin β4 protein sequence showed 92% homology with the bovine protein, 92% homology with the camel sequence, 89% homology with the human, and 88% homology with the mouse sequence.

### Detection of the genetic variation between the JEB-affected and unaffected skin samples via RNA-Seq

GATK [39] was used for variant detection in the RNA-seq data from the JEB-affected and unaffected samples. GATK can identify single base substitutions in addition to small insertions and deletions. In total, 495921 different variants were detected using the RNA-seq reads in both samples. The VEP tool from Ensembl processed a total of 490,196 variants, among which 5.2% (25247) corresponded to already-described variants and 94.8% (464949) corresponded to novel variants. There were 20,828 variants overlapping with known transcripts. Most of the variants detected were SNPs (91.5%), and 8.4% (40956) were indels.

Among the variants, we selected those that overlapped with the *ITGB4* gene. In total, 54 variants were found in the *ITGB4* region (OAR11: 54,848,050–54,874,496). Following the assumption that JEB in the Churra sheep is caused by a homozygous recessive mutation, the variants were sifted to retain those present at a homozygous stage in the affected case (1/1) and absent in the control animal (0/0). Fourteen variants matched this filter: nine intron variants, three synonymous variants, one missense variant, and one frameshift variant and feature truncation. We discarded the mutations located in introns, which had average DP (coverage) values of 3.2 in the case and 5.6 in the control, and the synonymous variants.

**Fig 3:**
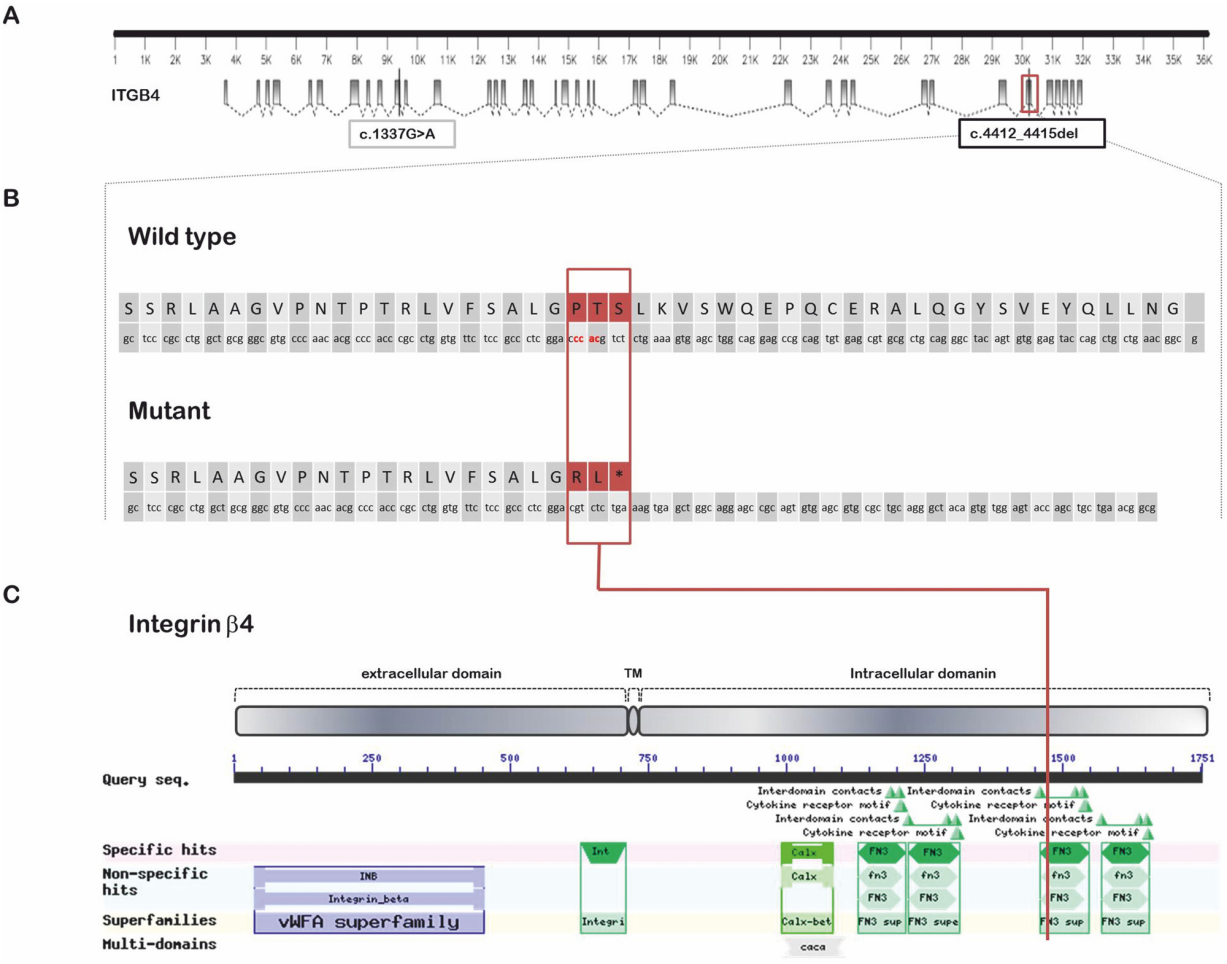
*ITGB4* mutation analysis. (A) Genomic structure of *ITGB4* sequence based in the information obtained by BAC clone sequencing and RNA-seq data. Grey boxes represent exons. Grey lines crossing the exons represent the two mutations with protein effects found in this study (*c*.*1337G>A* and *c*.*4412_4415del*). The red box highlights the exon 33 in which *c*.*4412_4415del* is located. (B) The first sequence corresponds to exon 33 and its translation in a wild type protein, the second sequence corresponds to mutant exon 33 and the resulting translation. The affected codons at exon 33 are highlighted in red. This 4-bp deletion leads to a truncated protein of 1,472 amino acids (1,470 amino acids of normal integrin β4 followed by two missense amino acids and a premature termination codon). (C) Putative resulting protein of *ITGB4* gene (GenBank KP025765). The mutation is predicted to delete the region spanning the last 281 amino acids in the intracellular domain, including the third and fourth FNIII repeats.

The missense variant detected in the RNA-seq variant analysis is a G>A substitution located at OAR11, which was 54,868,894 bp of the sheep Oar_v3.1 assembly. Regarding the *ITGB4* sequence (GenBank: KP025765) the mutation is positioned at 9,381 bp of the *ITGB4* genomic sequence. In the predicted CDS, the SNP is located at 1337 bp (*c*.*1337G>A*), in exon ten of the *ITGB4* gene (Fig 3). The mutation leads to an arginine-to-glutamine residue change at position 446 of the integrin β4 protein (*G>A p*.*Arg446Gln*). The genotype quality (GQ) value for *c*.*1337G>A* was 99 for the control and 42 for the case. In general, the determined coverage (DP value) for the *ITGB4* variants was higher in the control lamb than in the case; for this SNP the DP value was 75 and 14, respectively. This non-synonymous SNP has been observed at a homozygous stage in other sheep breeds (ISGC SNP database) and was predicted to be non-deleterious by SIFT [40] and PolyPhen-2 [41]. Therefore, the *c*.*1337G>A* variant was discarded as a putative causal mutation of JEB.

The frameshift variant detected in the RNA-seq variant analysis is a four-bp deletion located at OAR11:54,849,767–54,849,770 bp of sheep Oar_v3.1 assembly. In the ITGB4 sequence (GenBank: KP025765) the mutation is positioned at 30,204–30,207 bp of the ITGB4 genomic sequence. In the predicted CDS, the deletion is located at 4,412–4,415 bp (*c*.*4412_4415del*). The GQ value for the deletion was 99 for the control and 35 for the case, and the DP value was 180 and 11, respectively. This mutation is located on sheep *ITGB4* predicted exon 33 and leads to a shift in the open reading frame of the *ITGB4* coding sequence and a stop codon at position 1,473 (Fig 3). This mutation could lead to a truncated protein of 1,472 amino acids (1,470 amino acids of normal integrin β4 followed by two missense amino acids and a premature termination codon) in contrast to the 1,751-amino-acid length of the normal integrin β4 protein. Based on the deleterious effect on the composition and structure of the resulting protein, this 4-bp deletion was identified as the putative causal mutation for JEB in sheep.

### Functional analysis identifies ITGB4 as a causative gene

To quantify the levels of the *ITGB4* mRNA in the affected lambs and compare them with the levels in unaffected animals, we performed qRT-PCR using six replicates for each condition. The qRT-PCR confirmed that the *ITGB4* transcript levels were significantly lower in the cases than in the controls (relative expression average: 8.3 in the cases *vs* 100 in the controls; P<0.001). The mRNA expression analysis suggested that the JEB was likely caused by reduced expression of *ITGB4* in the skin.

**Fig 4:**
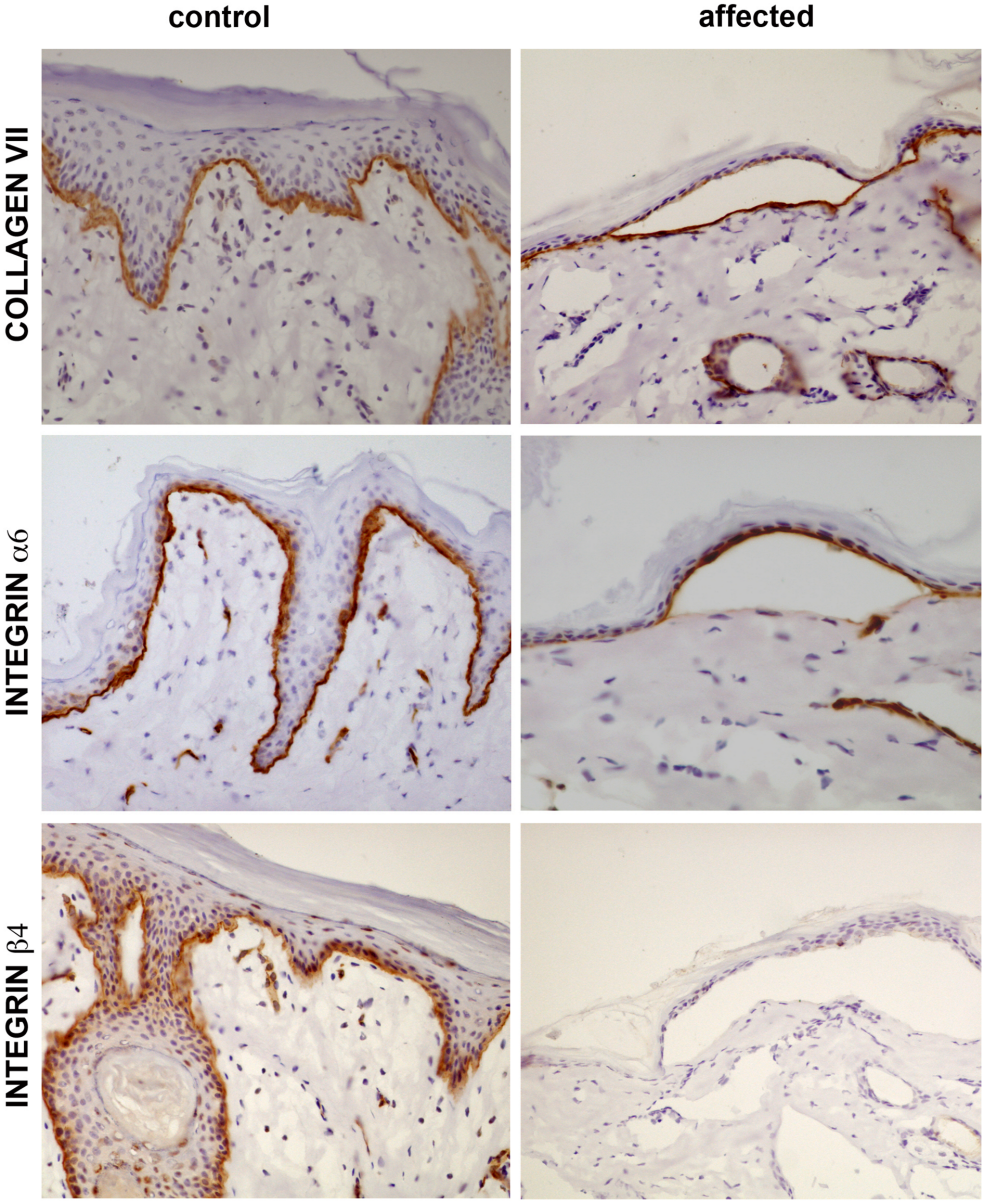
Immunohistochemical labeling of collagen VII and integrins α6 and β4. The three proteins are labeled (brown signal) in the skin samples of the control lamb, at the basal cell layer while in the samples from the affected lamb integrin β4. The signal of the other two proteins is normal. Note the localization of collagen VII at the base of the blister in the sample from the affected lamb while the integrin α6 remains attached to the basal keratinocytes, at the roof of the blister.

Immunohistochemical labeling of integrin β4 showed the absence of this protein in the skin of lambs with gross JEB lesions, whereas it was widely expressed in the basal cell layer in the control animal (Fig 4). The labeling of the two other epidermal basement membrane proteins that were studied, collagen VII and integrin α6, was similar between the control and the affected lambs. In the lambs showing gross JEB lesions, collagen VII was expressed primarily at the base of the blister, whereas integrin α6 was found at the roof. This labeling pattern confirms the localization of the cleavage between the lamina lucida and the lamina densa of the basement membrane (Fig 4).

### Validation of c.4412_4415del at the DNA level

After the putative causal mutation in the RNA-seq variant analysis was identified, the mutation was confirmed via analysis of 46 samples at the genomic DNA level. The association between the *c*.*4412_4415del* allelic variant and the JEB phenotype was confirmed by genotyping the deletion in the gDNA sequences of affected, alleged carrier and control animals. From the DNA samples, the amplicon of the putative exon 33 was obtained via PCR for 27 cases, 10 unrelated controls and sires and dams of affected lambs. The resulting PCR product was sequenced and analyzed using SeqScape v2.5 (Applied Biosystems). All of the samples from the JEB-affected animals were homozygous for the identified 4-bp deletion (*c*.*4412_4415del/c*.*4412_4415del or EB/EB*), the sires and dams for later animals were heterozygous (*EB/+*) and the controls from the different flocks were homozygous (*+/+*) for the wild-type allele.

## Discussion

We herein demonstrated that a 4-bp deletion within exon 33 of *ITGB4* gene (*c*.*4412_4415del*) is responsible for JEB in Churra sheep, based on medium-resolution mapping and RNA-seq variant analysis. The JEB locus was mapped to *Ovis aries* chromosome 11 via a GWA analysis using cases, related animals and unrelated controls. The region was fine-mapped to an 868-kb homozygous segment using IBD analysis. The *ITGB4* gene, which is located within this region, was the best positional and functional candidate gene. The RNA-seq variant analysis of control and JEB-affected skin RNA samples allowed us to find the causal mutation (*c*.*4412_4415del*). In this study, we found that individuals that are homozygous for this mutation lack the integrin β4 polypeptide. The mutation was confirmed at the genomic level, and three genotypes segregated with the disease status: homozygous mutants (*EB/EB*) were the affected lambs, heterozygous animals (*EB/+*) corresponded to the phenotypically normal carriers (ewes and rams of the affected lambs), and the wild-type genotype (*+/+*) was identified in all of the analyzed control individuals. These genotypes completely agreed with the autosomal recessive mode of Mendelian inheritance inferred for JEB in Churra sheep. The discovery of this mutation has allowed screening of affected flocks to identify phenotypically normal individuals carrying the 4-bp deletion to avoid at-risk mattings. Simple and rapid JEB genotyping methods like fragment length analysis in capillary electrophoresis sequencer and restriction fragment length polymorphism analysis are proposed (S2 Fig). The implementation of this gene-assisted selection approach will solve the problem of JEB in flocks of Churra sheep.

De novo BAC clone sequencing allowed us to obtain the complete *ITGB4* gene sequence (GenBank KP025765). The previous *ITGB4* ovine gene sequence (ENSOARG00000009764) was missing 16.6% of the bases when compared with KP025765 (S1 Fig). The RNA-seq approach enables the generation of extensive transcriptome information in the absence of a well-annotated genome. In the present study, the transcriptome information was essential in the annotation of the *ITGB4* gene sequence (GenBank: KP025765). The sheep *ITGB4* CDS obtained from the *KP025765* sequence is distributed into 38 exons and encodes a predicted protein of 1751 amino acids. The transcript available in Ensembl (ENSOART00000010637) has 42 exons and encodes a predicted protein of 1835 residues. The percentage of identity of the ovine integrin β4 protein with that of other species, such as cow (DAA18168.1) or human (AAI43743.1), is higher for the integrin β4 protein obtained from KP025765 (92% and 89%, respectively) than for the sequence obtained from the ENSOART00000010637 transcript (86% and 82%, respectively), which suggests that the *ITGB4* sequence obtained in this study is more reliable than the available sequence from the ovine genome assembly (Oar_v3.1).

Integrin β4 associates with integrin α6 to form a transmembrane constituent of hemidesmosomes anchoring complex [3]. Mutations in α6β4 have been associated with JEB-PA in humans (OMIM: #226730). Although several mutations have been described in these two genes, most mutations were found in the *ITGB4* gene (approximately 70), whereas only five cases have been reported in the *ITGA6* gene [42]. In this study we found a causal mutation in the *ITGB4* gene which leads into JEB in sheep. Several forms of EB have been associated with congenital absence of skin, primarily with DEB (Bart type (OMIM: #132000)). Churra lambs lack PA; however, previous reports on humans suggest that PA is not necessarily a feature of integrin-associated JEB [42, 43]. A recent report about JEB in cattle due to a deletion in *ITGB4* also describes the absence of PA [13]. Lack of PA could be due to the particular anatomical characteristics of ruminant’s pyloric sphincter, less muscular than in monogastric animals. The resemblance between the integrin β4-associated EB lesions described in humans [18], cattle [13] and ovine JEB supports the causative nature of the 4-bp deletion in the *ITGB4* gene that was reported here in relation to the JEB phenotype observed in Churra sheep.

The 4-bp deletion on putative exon 33 of the *ITGB4* gene (*c*.*4412_4415del*) results in a premature termination codon (PTC) and is predicted to delete the region spanning the last 281 amino acids in the intracellular domain (Fig 3), which has been shown to interact with the 180-kD bullous pemphigoid antigen [44]. This results in the loss of the C-terminal region, including the third and fourth FNIII repeats. The lack of labeling by the monoclonal antibody (clone 450-10D) used against integrinβ4 is consistent with this prediction, as this antibody binds to the intracellular region of the integrin [45]. The persistency of the extracellular domain could enable the formation of an α6β4 dimer. Also supporting this possibility, is the wide expression of integrin α6, which was observed via immunohistochemistry at its physiological location, the lamina lucida. However, the highly decreased amount of mRNA in the JEB skin samples compared with the control samples (identified through the qRT-PCR analysis) suggests that the truncated mRNA products are degraded by nonsense-mediated mRNA decay (NMD) machinery. Therefore, the absence of both the extracellular and intracellular domains, of integrin β4 cannot be ruled out, as deficiency of the whole protein would also produce a lack of labeling by the monoclonal antibody specific for the intracellular epitope. In humans, mutations related with PTC in both alleles are predominantly associated with lethal forms of the disease, whereas missense mutations are associated with mid-range phenotypes [46]. However, lethal/non-lethal consequences are primarily associated with the existence of a functional integrin β4, which, is why the immunological labeling of the protein is also important in the diagnostic procedure [21]. Similar to the integrin β4 subunit, when transmission electron microscopy (TEM) is performed in the skin samples of JEB lambs we could see that hemidesmosomes were also absent. Schaapveld et al (1998) [47] indicated that keratinocytes lacking β4 expression are not capable of forming organized hemidesmosome-like structures. No hemidesmosome structures were found in *ITGB4* knock-out mouse [48], it was thus proposed that alternative adhesion molecules, such as integrin α3β1, could explain the residual adhesion of the non-detached skin areas.

In conclusion, we have identified the causal mutation for ovine JEB in Churra sheep. This mutation (*c*.*4412_4415del*) is located in putative exon 33 of the *ITGB4* gene and leads to the formation of a premature termination codon without protein expression. The *ITGB4* gene can be rapidly applied as a selection marker for identification of carriers to avoid at-risk mattings, enabling the eradication of this lethal mutation from the Churra sheep breed. We have also demonstrated the successful combination of medium-density SNP panels with RNA-seq approaches for localization of mutant locus causing a recessive defect, which can serve as a reference for other studies performed in species in which high resolution exome sequencing is not straightforward. Furthermore, given the relatively low cost of maintaining the carriers, sheep with JEB could be used as a valuable large-animal model for the study of this disease and the development of therapeutic approaches.

## Material and Methods

### Ethics statement

The blood and tissue samples were collected from rams, ewes and lambs by qualified veterinarians following standard procedures and conducted under a license issued in accordance with the European Union legislation (European Community Directive, 86/609/E and Directive 2010/63/EU of the European Parliament and of the Council). All of the animals were managed in accordance with the guidelines for the accommodation and care of animals. The DNA samples used in this study were extracted from leucocytes in 3 ml of blood obtained via jugular venipuncture. The tissues were collected immediately after euthanasia (performed by a qualified veterinarian via intravenous injection of T-61 (Intervet)); animals were euthanized as standard of care. As the samples were from two commercial flocks that underwent veterinary examination, we were in a special situation in veterinary medicine and there was no “animal experiment” according to the legal definitions in Spain (Animal care legislation “Ley 32/2007”). According to the Ethics Commission of the University of León, formal ethical approval is not required under these circumstances.

### Animals

Blood and/or tissue samples were collected from a total of 27 JEB-affected lambs from two commercial flocks belonging to the Spanish Churra Sheep Breeders’ Association (ANCHE). Additionally, samples from 28 healthy Churra individuals from the same flocks were collected. The samples included the parents, siblings and half-siblings of the JEB-affected offspring. Fifty samples from unaffected Churra sheep from different flocks with unknown relatedness to the affected lambs were collected for use as controls.

### Genome-wide association and homozygosity mapping studies

In total, 96 DNA samples were genotyped using the Illumina OvineSNP50 BeadChip. These samples included 48 unrelated, healthy Churra individuals from different flocks of the Churra Selection Nucleus and 48 animals from the affected flock. Twenty of the animals from the affected flock were JEB lambs, and the rest of the animals were related to them (sires, dams, siblings and half-siblings).

The results were analyzed using PLINK, v1.07 [49]. A quality control procedure was performed to eliminate SNPs with genotyping rates lower than 0.05 and minor allele frequencies lower than 0.01 and to eliminate animals with call rates lower than 0.95. After the QC pruning, 44,785 SNPs were considered in the subsequent analysis. The—assoc option in PLINK was applied to perform a case-control genome-wide association study (GWAS). To correct empirical p-values, a one-million permutation procedure was performed.

After the GWAS was performed, a run of homozygosity or identity-by-descent (IBD) analysis was performed. The run of homozygosity is performed by filtering the cases to identify the allele-sharing regions. The SNPs flanking the consensus region obtained from this analysis were used to trace this region in the Ovine Genome Assembly Oar_v3.1 Browser (http://gbrowse-ext.bioinformatics.csiro.au/fgb2/gbrowse/oarv3/). The genes identified within the homozygosity block were evaluated as putative positional candidate genes.

### Isolation of the ovine ITGB4 gene

*ITGB4* was isolated from three sheep CHORI-243 bacterial artificial chromosome (BAC) libraries [50]. The selected BAC clones predicted to span the region in which the *ITGB4* gene is located were CH243-344E15, CH243-306J7 and CH243-504K6. The BACMAX DNA Purification Kit (Epicentre) was used for purification of the DNA.

Ion Torrent PGM data were generated from the three BAC libraries constructed from 500 ng of DNA quantitated using the Qubit Fluorometer 2.0 (Life Technologies, Grand Island, NY). The fragment libraries were constructed using the Ion Xpress Plus Fragment Library kit based on Ion Shear chemistry according to the user guide. The barcode and adaptor ligation were performed per the manufacturer’s protocol using the Ion Xpress Barcode Adapters 1–16 Kit. The quantitation and size distribution of the fragments were analyzed on an Agilent Bioanalyzer using the High Sensitivity Kit (Agilent). The template preparation and emulsion PCR were performed using the Ion PGM Template OT2 200 Kit (Life Technologies) according to the manufacturer's instructions. The template product then was quantitated using the Ion Sphere Quality Control Kit on at Qubit Fluorometer 2.0. OneTouch ES was used for the enrichment of the Ion Sphere Particles (ISP) template products. Finally, the samples were loaded onto a 316 chip V2 and sequenced using the Ion PGM 200 Sequencing Kit V2 (Life Technologies).

The reads were collected using Ion Torrent Suite v4.0, which also sorts the data according to the barcodes. The Torrent Suite software scores the quality of the reads by assigning Q20 scores according to Ion Torrent's quality scoring computation. MIRA (version 3.4.0) was used for de novo assembly of all of reads from each of the three BACs. The mean coverage was 120X for each BAC. The Torrent Mapping Alignment Program (TMAP) was used for alignment of all of the reads from each of the three BACs against the reference (Chr 19 Cow sequence; chromosome: UMD3.1:19:1:64057457:1). Both, the BAC DNA sequencing and the de novo assembly were performed at AC-gen Reading Life (Valladolid, Spain).

### RNA-seq analysis

For the RNA-seq analysis, skin samples from a control lamb and a JEB-affected lamb were collected for RNA extraction. The skin samples were harvested immediately after euthanasia and preserved in RNA Stabilization Reagent (RNAlater, Ambion). Slices of up to 500 mg of skin tissue were processed using the RNeasy Fibrous Tissue Midi Kit (Qiagen) to extract the total RNA. RNA integrity and concentration were evaluated using an Agilent Technologies 2100 Bioanalyzer. Both samples had RNA Integrity Number (RIN) values higher than 7.3. The TrueSeq RNA Sample Prep Kit v2 (Illumina) was used to prepare paired-end libraries with fragments of 300 bp. Two paired-end cDNA libraries were constructed, one for each of the two samples (JEB-affected and unaffected skin samples). The cDNA libraries were loaded onto the flow cell channels of an Illumina Hi-Seq 2000 platform to perform massive parallel sequencing at the CNAG (Centro Nacional de Análisis Genómico, Barcelona), generating 75-bp paired-end reads.

The *Ovis aries* genome, the annotation file and the reference variant file were downloaded from the Ensembl database (http://www.ensembl.org/index.html). The quality of the reads generated was assessed using the program FastQC (http://www.bioinformatics.babraham.ac.uk/projects/fastqc/). The paired ends for each sample were aligned to Oar v3.1 using STAR [36].

For the *ITGB4* gene prediction, Augustus software was used [37, 38]. Once the alignment to the reference genome was performed, Samtools [51] was used to sort, index and extract the IDs of the reads aligned within the *ITGB4* region from the bam files (OAR11: 54,830,000–54,880,000). Seqtk tool (https://github.com/lh3/seqtk) was used with the ID list from the bam file and the initial fastq file to create a new fastq file that contained only the reads that align within the *ITGB4* region. The new fastq files were mapped against the *ITGB4* sequence obtained from the BAC clones using STAR. Cufflinks [52] was used to assemble the bam files and to create an exonpart hints file for Augustus. The putative coding transcript for the *ITGB4* BAC clone sequence was identified with Augustus by providing the *ITGB4* sequence, exon boundaries and an integrin β4 protein profile and allowing the program to predict exactly one gene.

For variant detection, GATK was used [39]. The GATK analysis was performed on the STAR alignments following the RNA-seq workflow recommended in the best-practices section of the webpage for the GATK software [53, 54]. Prior to the variant analysis, the PCR duplicates from the bam files were removed using Picard tools (http://picard.sourceforge.net/). Then, an indel realignment and a base quality score recalibration were performed using the *Ovis aries* vcf file downloaded from Ensembl as a reference. These steps were followed by the variant calling analysis performed using the “–T HaplotypeCaller” option of GATK. The biological impact predictions for detected variants were obtained using Ensembl Variant Effect Predictor (VEP: http://www.ensembl.org/tools.html). Once the variant calling analysis was performed, the variants located in the *ITGB4* region were selected. These variants were filtered to keep only those located in exons homozygous for the reference sequence in the control and homozygous and distinct from the reference in the case.

### Quantification of the ITGB4 mRNA via qRT-PCR

Total RNA was isolated from the skin using the RNeasy Fibrous Tissue Midi Kit (Qiagen) according to the manufacturer's instructions. The-high-capacity cDNA Reverse Transcription Kit (Applied Biosystems) was used to synthesize the DNA according to the manufacturer's instructions. The primers were designed using Primer3 [55, 56] resulting in two primer sets (S1 Table) spanning the region containing the 4-bp-deletion. One primer set produced a 334-bp product and the second produced a 182-bp product. The expression profiling of *ITGB4* was performed on a StepOne Real-Time PCR System (Applied Biosystems) with the Power SYBR Green PCR Master Mix according to the manufacturer's instructions. *ITGB4* transcript-specific expression was evaluated via relative quantification against the sheep glyceraldehyde-3-phosphate dehydrogenase (*GAPDH*) expression level (ΔCt) using one of the control homozygous replicates samples as a calibrator.

### Immunohistochemical studies

Samples of grossly normal skin (metacarpal region) were taken from three lambs showing gross JEB lesions. Control samples, taken from the same area, were also obtained from one lamb from the same flock that was euthanized because of diarrhoea and not affected by JEB. The samples were snap-frozen immediately after euthanasia in 2-methylbutane cooled in liquid nitrogen and stored at -70°C until used. Frozen sections (5 μm) were cut with a cryostat, collected on slides and fixed in acetone for 10 min. The samples underwent immunohistochemical examination with monoclonal antibodies against collagen VII, clone LH7.2, (100 dilution; Neomarkers), integrin α6, clone GoH3 (100 dilution; Abcam) and integrin β4, clone 450-10D (2000 dilution; [45]) was carried out, using Dako EnVision System-HRP (Dako North America Inc.) with 3,3’diaminobenzidine (Dako North America Inc.) as chromogen substrate.

### Confirmation and genotyping of the causal mutation

DNA was extracted from the blood samples collected from the 27 cases, the 6 mothers and 3 half-brothers of the affected animals, and the 10 unrelated controls. Primers (*ITGB4*ovine_ex33fw: 5’- CCAGCAGTCAGGGAGGTG and *ITGB4*ovine_ex33rv: 5’- CTCACCGCCGTTCAGCAG) were designed using Primer3 [55, 56]. The primers *ITGB4*ovine_ex33fw and *ITGB4*ovine_ex33rv amplify a 243-bp fragment of the gene sequence that contains the 4-bp deletion that was identified has the putative causal mutation. The products were amplified via PCR in a 30 μL reaction volume containing 100 ng of gDNA, 480 μM dNTPs, 1.5 μM MgCl2, buffer 10X diluted 1X (Applied Biosystems), the specific primers at 0.5 μM, 0.25 M betaine and 0.75 μl of AmpliTaq Gold polymerase (5 U/ml) (Applied Biosystems). The PCR conditions were 5 min at 96°C; 40 cycles of 30 sec at 96°C, 45 sec at 58°C, and 40 sec at 72°C; and then a final extension of 10 min at 72°C. The amplicons were purified using Illustra Exonuclease I and alkaline phosphatase (Illustra ExoProStar 1-Step, GE Healthcare Life Sciences) treatment and were dideoxy-sequenced in both directions using the Big Dye Terminator Cycle Sequencing Kit, v3.1 (Applied Biosystems) using the same primers used for the fragment amplification. After sequencing on an ABI Prism 3130 automatic sequencer (Applied Biosystems), the sequence data were analyzed using SeqScape v2.5 (Applied Biosystems).

## Supporting Information

S1 Fig Ovine *ITGB4* genomic sequence. Genomic sequences of sheep: ENSOARG00000009764 and KP025765 are aligned using the *EMBOSS Needle* software (http://www.ebi.ac.uk/Tools/psa/emboss_needle/nucleotide.html).
(PDF)

S2 Fig Examples of rapid genotyping methods for the detection of JEB carriers. Examples for fluorescent fragment length analysis and RFLP used in carrier detection are displayed.
(PDF)

S1 Table Primers used in quantification of the *ITGB4* mRNA via qRT-PCR. The length of the amplified product, the melting temperature (Tm) and position in Oar_v3.1 are also indicated.
(XLSX)
