# Supplementary material for: Combining GWAS and RNA-Seq Approaches for Detection of the Causal Mutation for Hereditary Junctional Epidermolysis Bullosa in Sheep
Source: PLoS One. 2015 May 8;10(5):e0126416. doi: 10.1371/journal.pone.0126416 (PMC4425408; doi:10.1371/journal.pone.0126416)
Supplement: S1 Fig — Genomic sequences of sheep: ENSOARG00000009764 and KP025765 are aligned using the EMBOSS Needle software (http://www.ebi.ac.uk/Tools/psa/emboss_needle/nucleotide.html). (PDF) [file pone.0126416.s001.pdf]

```
########################################
# Program: needle
# Rundate: Mon 15 Dec 2014 16:30:11
# Commandline: needle
#    -auto
#    -stdout
#    -asequence emboss_needle-I20141215-162649-0501-16776341-es.asequence
#    -bsequence emboss_needle-I20141215-162649-0501-16776341-es.bsequence
#    -datafile EDNAFULL
#    -gapopen 10.0
#    -gapextend 0.5
#    -endopen 10.0
#    -endextend 0.5
#    -aformat3 pair
#    -snucleotide1
#    -snucleotide2
# Align_format: pair
# Report_file: stdout
########################################

#=======================================
#
# Aligned_sequences: 2
# 1: ENSOARG00000009764
# 2: KP025765
# Matrix: EDNAFULL
# Gap_penalty: 10.0
# Extend_penalty: 0.5
#
# Length: 32985
# Identity:   27411/32985 (83.1%)
# Similarity: 27411/32985 (83.1%)
# Gaps:        5486/32985 (16.6%)
# Score: 135284.0
#
#
#=======================================

ENSOARG000000      1 --------------------------------------------------      0

KP025765           1 ggctcgcccgcgcgccgcatctggagccgccgagcggagcccagcgcggg     50

ENSOARG000000      1 --------------------------------------------------      0

KP025765          51 gagggtgcgagtccgccccaaggtaggtccagccattcccccgcactccg    100

ENSOARG000000      1 --------------------------------------------------      0

KP025765         101 cccgcccctccagggccccgagccgccgaggctgggcgcgctctaggagg    150

ENSOARG000000      1 --------------------------------------------------      0

KP025765         151 tttccgaggggagaagcggaaagggagtgcgcaccccagccccgggcagc    200

ENSOARG000000      1 --------------------------------------------------      0

KP025765         201 ccgacggggagctgattggtgggccggcggcttttgtttccccagctctt    250

ENSOARG000000      1 --------------------------------------------------      0

KP025765         251 cctttcctaaaggccggccgcccagggcaggtgccctgcctgcccgggtg    300

ENSOARG000000      1 --------------------------------------------------      0
```

```
KP025765         301 tgtccaggggacccccggcggaaagggaacaggcagagctggagaaggcg    350

ENSOARG000000      1 --------------------------------------------------      0

KP025765         351 cttggttgtcagacctctgggtggaggagggcagcagggagaggtctggg    400

ENSOARG000000      1 --------------------------------------------------      0

KP025765         401 cgtcgggtgggcgcttgggagaagtagtgggcaggggcccggtgggtggc    450

ENSOARG000000      1 --------------------------------------------------      0

KP025765         451 ctactgggctgggcacttctggggttcaaaaagaagcagccacctagagg    500

ENSOARG000000      1 --------------------------------------------------      0

KP025765         501 cagagaccctccgatttggctgggtggtgccaccgtgcagctggggtctg    550

ENSOARG000000      1 --------------------------------------------------      0

KP025765         551 gggactccttgattctcctctcctcctggctgggcacacgtctgccttgc    600

ENSOARG000000      1 --------------------------------------------------      0

KP025765         601 tcccaaagtcagctcaggacacccttcctccaggaagccctcctgacctc    650

ENSOARG000000      1 --------------------------------------------------      0

KP025765         651 atggcccaccactgtgacctcctccagggccagcccccctgtccccaccct    700

ENSOARG000000      1 --------------------------------------------------      0

KP025765         701 ctcagccccgggtggccctctccggttgtaggtggggacctctagatggc    750

ENSOARG000000      1 --------------------------------------------------      0

KP025765         751 tcctcctgggctccttcttctccccagtggcctggccacaccctgagccc    800

ENSOARG000000      1 --------------------------------------------------      0

KP025765         801 aaggctggacggggttgtggctgcctcagaccacagctggatgtgaagtc    850

ENSOARG000000      1 --------------------------------------------------      0

KP025765         851 tgcaggtctctgtcccagggggctgagcgggtaaaggaaaacaggaggga    900

ENSOARG000000      1 --------------------------------------------------      0

KP025765         901 cagcggggaaggggtgtccgtctgtctccttggccttgaatctgcgggtg    950

ENSOARG000000      1 --------------------------------------------------      0

KP025765         951 atagagcagcagaggggccagaggatagggaagcaggagggaagagagct   1000

ENSOARG000000      1 --------------------------------------------------      0

KP025765        1001 gtggagggcgggtgcagacgtgccaagcgggggtcccatggggtaggtat   1050

ENSOARG000000      1 --------------------------------------------------      0

KP025765        1051 caggagaacgctgagcccacattatgtgggccatagcgtgtgtccaagag   1100

ENSOARG000000      1 --------------------------------------------------      0

KP025765        1101 tatgtatgtccctgagcctgccagactgggtgtgccgctatgtgataaca   1150
```

```
ENSOARG000000      1 --------------------------------------------------      0

KP025765        1151 gtgggtggcaagcactcacttccgggacccggctgctacataaaaaggcc   1200

ENSOARG000000      1 --------------------------------------------------      0

KP025765        1201 ccgcgcctggtgggacccagagtagcctggagggttcgtgttccaacggc   1250

ENSOARG000000      1 --------------------------------------------------      0

KP025765        1251 tcttcccatgccgccacagtcacgtctcccaggaagcccacagtcctctt   1300

ENSOARG000000      1 --------------------------------------------------      0

KP025765        1301 atgtgacatcttttcacccaccaatgactactttgaaaactttttaggga   1350

ENSOARG000000      1 --------------------------------------------------      0

KP025765        1351 ggccccttggtggtccagggcttcccaggtgccaaagtggtaaggagtcc   1400

ENSOARG000000      1 --------------------------------------------------      0

KP025765        1401 gcctgccaatgcaggagacacgggtttgatccctgggtcaggaagatccc   1450

ENSOARG000000      1 --------------------------------------------------      0

KP025765        1451 ctggaagagaagatagcaacccactccagtattcttgcctggaaaactcc   1500

ENSOARG000000      1 --------------------------------------------------      0

KP025765        1501 catagacagcagggcctggcggactccagtctgtggggtagcaaagagtc   1550

ENSOARG000000      1 --------------------------------------------------      0

KP025765        1551 ggacatgcctgagcatgcatgcagtggtggtccagtggataggaatcggt   1600

ENSOARG000000      1 --------------------------------------------------      0

KP025765        1601 gctctcactgtctgggcttggggtcaatccctggtcagggaaccaaggtc   1650

ENSOARG000000      1 --------------------------------------------------      0

KP025765        1651 ccacaagtcactcggtatggccaaaaacaaaaaagctttttaggcagttc   1700

ENSOARG000000      1 --------------------------------------------------      0

KP025765        1701 cctggtggccaggtggttaagactccatgctcccaacacaggggtcacag   1750

ENSOARG000000      1 --------------------------------------------------      0

KP025765        1751 gttcatttcctggttggggaactaaaatttctcatgctgcaagattcagc   1800

ENSOARG000000      1 --------------------------------------------------      0

KP025765        1801 cttagaaaaaaaaaaaaaagtttaaaaacacaagttgagaaaaacacaac   1850

ENSOARG000000      1 --------------------------------------------------      0

KP025765        1851 actcctgtgtgcatcgctgctattttagagtctctgaatataatgtgaag   1900

ENSOARG000000      1 --------------------------------------------------      0

KP025765        1901 gttaaatggggcgggggtgctgctgctgcactgccccgtctcgtctggac   1950
```

```
ENSOARG000000      1 --------------------------------------------------      0

KP025765        1951 aaaggggagcaggcagtcattcccccactggttcctgtgcagattaaatg   2000

ENSOARG000000      1 --------------------------------------------------      0

KP025765        2001 gcgattatgtacgtgacatgctgggcacttgcccattgtgtgcccagcct   2050

ENSOARG000000      1 --------------------------------------------------      0

KP025765        2051 gggggcacaggagagtgagtaagagccgggttgggcagtgcctgtacctg   2100

ENSOARG000000      1 --------------------------------------------------      0

KP025765        2101 catgtataatggtgccccagggaagcccggggaagcagccagtattgtgt   2150

ENSOARG000000      1 --------------------------------------------------      0

KP025765        2151 agcctgtgggtgtggtgcctgtgccccaaggctgcaggaaaaccatttcc   2200

ENSOARG000000      1 --------------------------------------------------      0

KP025765        2201 ccccaagaagcacccctcctcggctggcttggggcacccttaccccaggc   2250

ENSOARG000000      1 --------------------------------------------------      0

KP025765        2251 ccatcttagaggcgggctcctccccagctgactgcctgtggatgaggtgg   2300

ENSOARG000000      1 --------------------------------------------------      0

KP025765        2301 agctcctggtcgccacctcggagagacggaagcatgccctcctgcagccc   2350

ENSOARG000000      1 --------------------------------------------------      0

KP025765        2351 ctcgggtctccccagagcctggggtgtgcctgccccacaagccaaggcct   2400

ENSOARG000000      1 --------------------------------------------------      0

KP025765        2401 gagcgaggactcaaggctgagggccagtaggcatggcctagtcgcgggct   2450

ENSOARG000000      1 --------------------------------------------------      0

KP025765        2451 gctgggtgactggctctggagaaggaggccgggcggtcactcttgccctc   2500

ENSOARG000000      1 --------------------------------------------------      0

KP025765        2501 agctctgccaggccaggcgcctctggccagctcttctcttcagcaggctg   2550

ENSOARG000000      1 --------------------------------------------------      0

KP025765        2551 tccatcttgaaacgttctcaggaatgtcactgcctgcccccgaaggtcac   2600

ENSOARG000000      1 --------------------------------------------------      0

KP025765        2601 caatgactcgagatggtttaaaagttgaaccaaaggggccttccctgggt   2650

ENSOARG000000      1 --------------------------------------------------      0

KP025765        2651 cagtgataaaagaatctgcctgccagtgcaggagacacgggttcaatccc   2700

ENSOARG000000      1 --------------------------------------------------      0

KP025765        2701 tgatccaggaagatcccacacgccgaggaggagctaagcccgtgtgccac   2750

ENSOARG000000      1 --------------------------------------------------      0
```

```
KP025765         2751 accactgagactgtgccctagagcccaggagctgcaagtagagaacccac   2800

ENSOARG000000       1 --------------------------------------------------      0

KP025765         2801 cgcagggagaaccctgctcaccgcaattgaggagaaggcctagcacagcc   2850

ENSOARG000000       1 --------------------------------------------------      0

KP025765         2851 aaaagcaatgaataaataaatcgttttaaaaaaaacagtgactcaaaggc   2900

ENSOARG000000       1 --------------------------------------------------      0

KP025765         2901 agatggggtgtcctggggtggctggcagtgtgtgtgtgtatgagtgggct   2950

ENSOARG000000       1 --------------------------------------------------      0

KP025765         2951 gcaggggcttttaatccccctccaggctaactttctaaagggtgatctgc   3000

ENSOARG000000       1 --------------------------AGGCACAGGTGCGGGGACCCCCTC     24
                                                ||||||||||||||||||||||||
KP025765         3001 tcagaatcccccaactcaactcgggcaggcacaggtgcggggaccccctc   3050

ENSOARG000000      25 CTCTGATGGGCCAGGCAGCCATTACAGAACTTAGCTGAGTAGCAAATCAT     74
                      ||||||||||||||||||||||||||||||||||||||||||||||||||
KP025765         3051 ctctgatgggccaggcagccattacagaacttagctgagtagcaaatcat   3100

ENSOARG000000      75 GTCCTCCCTTACCTAGGCCCCCAGTACTGCAGAGGGTCCCCCTCCTTCTA    124
                      ||||||||||||||||||||||||||||||.|||||||||||||||||||
KP025765         3101 gtcctcccttacctaggcccccagtactgcggagggtccccctccttcta   3150

ENSOARG000000     125 GTCTGTATCCCATCACCGGAGGGCAAGTAAGCGGGCCAGGGCCCTCTAGG    174
                      ||||||||||||||||||||||||||||||||||||||||||||||||||
KP025765         3151 gtctgtatcccatcaccggagggcaagtaagcgggccagggccctctagg   3200

ENSOARG000000     175 GTGGCCCTGTCCCTTCCAAGCCTGACCCAACCCAGTGGGGTCTGAAAGGC    224
                      ||||||||||||||||||||||||||||||||||||||||||||||||||
KP025765         3201 gtggccctgtcccttccaagcctgacccaacccagtggggtctgaaaggc   3250

ENSOARG000000     225 GAGGGTGGAAAAAAATCCCTGGTCTTCCCTCCAGGGAGCCCAGTACAAGA    274
                      ||||||||||||||||||||||||||||||||||||||||||||||||||
KP025765         3251 gagggtggaaaaaaatccctggtcttccctccagggagcccagtacaaga   3300

ENSOARG000000     275 GATCTAGCGGTGGGACAAGCTCAGAGCCCACAGTCCTCAGTGGTGAGAGG    324
                      ||||||||||||||||||||||||||||||||||||||||||||||||||
KP025765         3301 gatctagcggtgggacaagctcagagcccacagtcctcagtggtgagagg   3350

ENSOARG000000     325 GTGGGGGCCTCCCTGGCAGGGCGTAGGCCTACAGGGCATCTGGACACTCT    374
                      ||||||||||||||||||||||||||||||||||||||||||||||||||
KP025765         3351 gtgggggcctccctggcagggcgtaggcctacagggcatctggacactct   3400

ENSOARG000000     375 GGGGATGCCAAGGAAGGGGGCAGAGGAAAAGAGGGATCAGGGGTCCTGGG    424
                      ||||||||||||||||||||||||||||||||||||||||||||||||||
KP025765         3401 ggggatgccaaggaagggggcagaggaaaagagggatcaggggtcctggg   3450

ENSOARG000000     425 CCCTGCCACTTGTAGGCCAGGTGGCCTCGGAACATCACTAAAATTCTCAG    474
                      ||||||||||||||||||||||||||||||||||||||||||||||||||
KP025765         3451 ccctgccacttgtaggccaggtggcctcggaacatcactaaaattctcag   3500

ENSOARG000000     475 AGCCTCAGTTTGTACAGCTGTGCAATGGGAACGGTGGAGCTCTGATGAGG    524
                      ||||||||||||||||||||||||||||||||||||||||||||||||||
KP025765         3501 agcctcagtttgtacagctgtgcaatgggaacggtggagctctgatgagg   3550

ENSOARG000000     525 ACCCAGTGAGCTGACACGTGGACCAGGGTTGTCAGGCATCATGTGTCAAT    574
                      ||||||||||||||||||||||||||||||||||||||||||||||||||
```

```
KP025765          3551 acccagtgagctgacacgtggaccagggttgtcaggcatcatgtgtcaat   3600

ENSOARG000000      575 TGTGGGTGTTTTACAGGAAGAGGAGGATGGCAGGGCCACATCCCAGCCCG    624
                       ||||||||||||||||||||||||||||||||||||||||||||||||||
KP025765          3601 tgtgggtgttttacaggaagaggaggatggcagggccacatcccagcccg   3650

ENSOARG000000      625 TGGACCAGGCTGCTGCTGGCAGCCCTGCTGAGCGTCAGCTTCCCCGGGGA    674
                       ||||||||||||||||||||||||||||||||||||||||||||||||||
KP025765          3651 tggaccaggctgctgctggcagccctgctgagcgtcagcttccccgggga   3700

ENSOARG000000      675 CATGGGTGAGTCGGACACGCCCCTGCAGCACCCTCCTTGGTGGGGCCTCA    724
                       |||||||||||.||||||||||||||||||||||||||||||||||||||
KP025765          3701 catgggtgagttggacacgcccctgcagcaccctccttggtggggcctca   3750

ENSOARG000000      725 GCTTTCAAGGCCCTGCCCTTAAGGTATCAGGGGTGTGGGGGGAGGCTGTC    774
                       ||||||||||||||||||||||||||||||||||||||||||||||||||
KP025765          3751 gctttcaaggccctgcccttaaggtatcaggggtgtggggggaggctgtc   3800

ENSOARG000000      775 ACTCAGTGTCTGGCCCAGACCCTCACTCAGCCATTCAGCAAACAGCCACC    824
                       ||||||||||||||||||||||||||||||||||||||||||||||||||
KP025765          3801 actcagtgtctggcccagaccctcactcagccattcagcaaacagccacc   3850

ENSOARG000000      825 ACCCACTTCAAGGGTTGTGGAGCCATGATCCTGAGCTGCATGGCCCAAAC    874
                       ||||||||||||||||||||||||||||||||||||||||||||||||||
KP025765          3851 acccacttcaagggttgtggagccatgatcctgagctgcatggcccaaac   3900

ENSOARG000000      875 ACTTAGGGAAGCGCAAGGAGACTTGGTCTGAAGGAGGGAAAAGACATCCA    924
                       ||||||||||||||||||||||||||||||||||||||||||||||||||
KP025765          3901 acttagggaagcgcaaggagacttggtctgaaggagggaaaagacatcca   3950

ENSOARG000000      925 CTCAGAGAAGGCAGGCGCTTGCAGGGTGTGCTAAGGCCTGGACAGCGGAG    974
                       ||||||||||||||||||||||||||||||||||||||||||||||||||
KP025765          3951 ctcagagaaggcaggcgcttgcagggtgtgctaaggcctggacagcggag   4000

ENSOARG000000      975 TTGAAAGATTTTATGGGAATTTGAAGGAGAGGGAGGTGATCGACACAGGC   1024
                       ||||||||||||||||||||||||||||||||||||||||||||||||||
KP025765          4001 ttgaaagattttatgggaatttgaaggagagggaggtgatcgacacaggc   4050

ENSOARG000000     1025 TCTCTGAGTGTCTGCAGTCCGCAAAGGGCTCCTACTGATGGAATAGTGCC   1074
                       |||||||||||||||||||||||||||||||||||||||||||||||.||
KP025765          4051 tctctgagtgtctgcagtccgcaaagggctcctactgatggaatagtacc   4100

ENSOARG000000     1075 TGTCAGTGGACGCCATGGCTGGCAGCTCAAAGCAGTCCGTGGTGGGAACA   1124
                       ||||||||||||||||||||||||||||||||||||||||||||||||||
KP025765          4101 tgtcagtggacgccatggctggcagctcaaagcagtccgtggtgggaaca   4150

ENSOARG000000     1125 TTTATACTTTGGAAATCGGTAAATGCTGCAAGTCAGGGCTTCCCTGCCCT   1174
                       ||||||||||||||||||||||||||||||||||||||||||||||||||
KP025765          4151 tttatactttggaaatcggtaaatgctgcaagtcagggcttccctgccct   4200

ENSOARG000000     1175 CACCGGAGACACTGCTAAACATTGACAGCACTCCTTTTCCTGAAAGTCCA   1224
                       ||||||||||||||||||||||||||||||||||||||||||||||||||
KP025765          4201 caccggagacactgctaaacattgacagcactccttttcctgaaagtcca   4250

ENSOARG000000     1225 GGAGCCCTGCCCACTAGCAAGGGCGGGTCCTCACTGTGATTTTAACTGAA   1274
                       ||||||||||||||||||||||||||||||||||||||||||||||||||
KP025765          4251 ggagccctgcccactagcaagggcgggtcctcactgtgattttaactgaa   4300

ENSOARG000000     1275 CAAAACCAGACGGTATCCTAACCTTTGGGGAAACTGAAGTTTCACCTCAT   1324
                       ||||||||||||||||||||||||||||||||||||||||||||||||||
KP025765          4301 caaaaccagacggtatcctaacctttggggaaactgaagtttcacctcat   4350

ENSOARG000000     1325 TTATTTATTAAAAACTTACCATGTGCAGGGCATAGGCTCAGCACTAGAGA   1374
                       ||||||||||||||||||||||||||||||||||||||||||||||||||
KP025765          4351 ttatttattaaaaacttaccatgtgcagggcataggctcagcactagaga   4400
```

```
ENSOARG000000   1375 CGCTGATAAAAGAAACACAGTCCCGGAGGAGCAGGCAAGGGGGCTGGTTA   1424
                     ||||||||||||||||||||||||||||||||||||||||||||||||||
KP025765        4401 cgctgataaaagaaacacagtcccggaggagcaggcaagggggctggtta   4450

ENSOARG000000   1425 GGGTGTCCCCGCCAGCAAATGGGCCAGCGCCTCCATCTCAGCCCCTACCC   1474
                     ||||||||||||||||||||||||||||||||||||||||||||||||||
KP025765        4451 gggtgtccccgccagcaaatgggccagcgcctccatctcagcccctaccc   4500

ENSOARG000000   1475 TCCTTCCCTCCAGACTTGGGTGGAAAATCCCAGTTTGAGACCTGGCTCTT   1524
                     ||||||||||||||||||||||||||||||||||||||||||||||||||
KP025765        4501 tccttccctccagacttgggtggaaaatcccagtttgagacctggctctt   4550

ENSOARG000000   1525 CAGGTTGCTAACCACATAGCCACTGGCCTCTGTTTCCCCCTCTGTATCTT   1574
                     ||||||||||||||||||||||||||||||||||||||||||||||||||
KP025765        4551 caggttgctaaccacatagccactggcctctgtttccccctctgtatctt   4600

ENSOARG000000   1575 GGAAGAGAGTCCCTACCTCTTTCAAATGAGGATGCTTAAGGGGAGGGCTT   1624
                     ||||||||||||||||||||||||||||||||||||||||||||||||||
KP025765        4601 ggaagagagtccctacctctttcaaatgaggatgcttaaggggagggctt   4650

ENSOARG000000   1625 GGTCCTGGGGGAGACCTATCTCAGTGGGCCCACCCCGCTCTGTTCCGCAG   1674
                     ||||||||||||||||||||||||||||||||||||||||||||||||||
KP025765        4651 ggtcctgggggagacctatctcagtgggcccaccccgctctgttccgcag   4700

ENSOARG000000   1675 CGAACCGCTGCAAGAAGGCCCAGGTGAAGAGCTGCACCGAGTGCATCCGC   1724
                     ||||||||||||||||||||||||||||||||||||||||||||||||||
KP025765        4701 cgaaccgctgcaagaaggcccaggtgaagagctgcaccgagtgcatccgc   4750

ENSOARG000000   1725 GTGGACAAGGACTGCGCCTACTGCACGGACGAGGTGTGTTGCTGCCCAGC   1774
                     ||||||||||||||||||||||||||||||||||||||||||||||||||
KP025765        4751 gtggacaaggactgcgcctactgcacggacgaggtgtgttgctgcccagc   4800

ENSOARG000000   1775 CGGACCGCCTCCCCAGCCTGTGCACCCACCCCTGGGCTCTGGCCGAGTCC   1824
                     ||||||||||||||||||||||||||||||||||||||||||||||||||
KP025765        4801 cggaccgcctccccagcctgtgcacccacccctgggctctggccgagtcc   4850

ENSOARG000000   1825 ACATTTGCCCCACCCTCGGCCCAGCCTTGGGCCAGCCGGGCGTGGGACAA   1874
                     ||||||||||||||||||||||||||||||||||||||||||||||||||
KP025765        4851 acatttgccccaccctcggcccagccttgggccagccgggcgtgggacaa   4900

ENSOARG000000   1875 TGGGCAAGGGTTCAGGAAAATCGAGGGGTGTAAATCTGGGGTTAGGAGAG   1924
                     ||||||||||||||||||||||||||||||||||||||||||||||||||
KP025765        4901 tgggcaagggttcaggaaaatcgaggggtgtaaatctggggttaggagag   4950

ENSOARG000000   1925 CAGCGGGGACAGGCACGGCCCCCCTGACCCCGTCTCCTTCTGGCCAGGTG   1974
                     ||||||||||||||||||||||||||||||||||||||||||||||||||
KP025765        4951 cagcggggacaggcacggcccccctgaccccgtctccttctggccaggtg   5000

ENSOARG000000   1975 TTCAAGGAACGTCGCTGCAACACCCAGGCTGAGCTGCTGGCTGCCGGCTG   2024
                     ||||||||||||||||||||||||||||||||||||||||||||||||||
KP025765        5001 ttcaaggaacgtcgctgcaacacccaggctgagctgctggctgccggctg   5050

ENSOARG000000   2025 CCGGCTGCAGAGTGTGGTGGTCATGGAGAGCAGCTACGAGATCATGGAGG   2074
                     ||||||||||||||||||||||||||||||||||||||||||||||||||
KP025765        5051 ccggctgcagagtgtggtggtcatggagagcagctacgagatcatggagg   5100

ENSOARG000000   2075 TGCCCGGGGTTGGGGGTAGGGGGAGGCAAGCTCAAGGCCAGCTGCTTATG   2124
                     ||||||||||||||||||||||||||||||||||||||||||||||||||
KP025765        5101 tgcccggggttgggggtagggggaggcaagctcaaggccagctgcttatg   5150

ENSOARG000000   2125 GGGTGTTATGGGGTCCCCTGGCTGAGCGGTCTCTCCATTAGATCTGGAGA   2174
                     ||||||||||||||||||||||||||||||||||||||||||||||||||
KP025765        5151 gggtgttatggggtcccctggctgagcggtctctccattagatctggaga   5200
```

```
ENSOARG000000   2175 GGCCCTCCAGCCAGCTGTCCTCCTCCCTTGGGCACAGGAAAGGCAGATCG   2224
                     ||||||||||||||||||||||||||||||||||||||||||||||||||
KP025765        5201 ggccctccagccagctgtcctcctcccttgggcacaggaaaggcagatcg   5250

ENSOARG000000   2225 ACACCACGCTGCGCCGCAGCCAAGTGTCCCCTCAGGCACTGCGGGTGCGG   2274
                     ||||||||||||||||||||||||||||||||||||||||||||||||||
KP025765        5251 acaccacgctgcgccgcagccaagtgtcccctcaggcactgcgggtgcgg   5300

ENSOARG000000   2275 CTGCGGCCGGGCGAGGAGCGGCACTTCGAGCTGCAGGTGTTTGAGCCCCT   2324
                     ||||||||||||||||||||||||||||||||||||||||||||||||||
KP025765        5301 ctgcggccgggcgaggagcggcacttcgagctgcaggtgtttgagcccct   5350

ENSOARG000000   2325 GGAGAGCCCCATGGACCTGTATATCCTCATGGACTTCTCCAACTCCATGT   2374
                     ||||||||||||||||||||||||||||||||||||||||||||||||||
KP025765        5351 ggagagccccatggacctgtatatcctcatggacttctccaactccatgt   5400

ENSOARG000000   2375 CTGATGATCTGGACAACCTCAAGAAGATGGGGCAGGATCTGGGTATGGAG   2424
                     ||||||||||||||||||||||||||||||||||||||||||||||||||
KP025765        5401 ctgatgatctggacaacctcaagaagatggggcaggatctgggtatggag   5450

ENSOARG000000   2425 AGGAAGACTGCGGGGCATGGAGAGGGTGCTCCCCCACCCCCAGCTGCTCA   2474
                     ||||||||||||||||||||||||||||||||||||||||||||||||||
KP025765        5451 aggaagactgcggggcatggagagggtgctcccccacccccagctgctca   5500

ENSOARG000000   2475 GGAGGCCAGGGCTCAGGCTAGCTATACCCACTCCTCAAGAAAGGACAGGA   2524
                     ||||||||||||||||||||||||||||||||||||||||||||||||||
KP025765        5501 ggaggccagggctcaggctagctatacccactcctcaagaaaggacagga   5550

ENSOARG000000   2525 CTTCTGAGCACCCCAAGAACTCCTCCCCATTCTCAGAGGAGAGTCCTGGC   2574
                     ||||||||||||||||||||||||||||||||||||||||||||||||||
KP025765        5551 cttctgagcaccccaagaactcctccccattctcagaggagagtcctggc   5600

ENSOARG000000   2575 CAGACCCCCAGTAACCACTCAGCAGTACAGAGGAGGGCCCCACTGGGTGG   2624
                     ||||||||||||||||||||||||||||||||||||||||||||||||||
KP025765        5601 cagacccccagtaaccactcagcagtacagaggagggccccactgggtgg   5650

ENSOARG000000   2625 GCCCCAGACCCCCTCTGGTCCGTTTTGGGTTTTTTGGCTGGACCGTGTCA   2674
                     ||||||||||||||||||||||||||||||||||||||||||||||||||
KP025765        5651 gccccagaccccctctggtccgttttgggttttttggctggaccgtgtca   5700

ENSOARG000000   2675 CGTGGCTTGTGGGATCTTAATACCCTGACCAGGGGTCAAACCCGTGCCTC   2724
                     ||||||||||||||||||||||||||||||||||||||||||||||||||
KP025765        5701 cgtggcttgtgggatcttaataccctgaccaggggtcaaacccgtgcctc   5750

ENSOARG000000   2725 TGTAGTGGCAACCCAGAGTTCTAGCCATTGGACTGCCAGGAAATGCCCTC   2774
                     ||||||||||||||||||||||||||||||||||||||||||||||||||
KP025765        5751 tgtagtggcaacccagagttctagccattggactgccaggaaatgccctc   5800

ENSOARG000000   2775 CTCTGGTCCTGACAAGGAGGGTCCCGGCTGCTACGAGGCTGAGGTTAAGT   2824
                     ||||||||||||||||||||||||||||||||||||||||||||||||||
KP025765        5801 ctctggtcctgacaaggagggtcccggctgctacgaggctgaggttaagt   5850

ENSOARG000000   2825 CTGAGCTCAACAGATGTCTGGTCCAGCTCCCCACCCGCCCCCTCCAACTT   2874
                     ||||||||||||||||||||||||||||||||||||||||||||||||||
KP025765        5851 ctgagctcaacagatgtctggtccagctccccacccgccccctccaactt   5900

ENSOARG000000   2875 GCTGCATGTGTGACTCCGGGTCCCCTGAGTCCCATTCCCTTCATCTGTAA   2924
                     ||||||||||||||||||||||||||||||||||||||||||||||||||
KP025765        5901 gctgcatgtgtgactccgggtcccctgagtcccattcccttcatctgtaa   5950

ENSOARG000000   2925 ATAATGACAGGGCTGCTGTGTGCACCAACTGATGCCAAACATGCAAAGCG   2974
                     ||||||||||||||||||||||||||||||||||||||||||||||||||
KP025765        5951 ataatgacagggctgctgtgtgcaccaactgatgccaaacatgcaaagcg   6000

ENSOARG000000   2975 TTGACGTTAAGTTATTAAGTTATTCTTCCTCTTCCTCCTTCCCAAACCAG   3024
```

```
                          ||||||||||||||||||||||||||||||||||||||||||||||||||
KP025765          6001 ttgacgttaagttattaagttattcttcctcttcctccttcccaaaccag   6050

ENSOARG000000     3025 CCCCCAACACTTATGTGGATGTGGGGTTCTTGGGGCTCAGCTGCCCTTTC   3074
                       ||||||||||||||||||||||||||||||||||||||||||||||||||
KP025765          6051 cccccaacacttatgtggatgtggggttcttggggctcagctgccctttc   6100

ENSOARG000000     3075 TTTTTGTTTTTAAAAAGTTTATTTATTTTTAATTGAAGGATATTGCTTCA   3124
                       ||||||||||||||||||||||||||||||||||||||||||||||||.|
KP025765          6101 tttttgtttttaaaaagtttatttatttttaattgaaggatattgcttta   6150

ENSOARG000000     3125 CAACATTGCATTGGTTTCCGCCATACACCAACATGAATCAGCCGTAGGTG   3174
                       ||||||||||||||||||||||||||||||||||||||||||||||||||
KP025765          6151 caacattgcattggtttccgccatacaccaacatgaatcagccgtaggtg   6200

ENSOARG000000     3175 TACATATGACCCCCTCCCTCTTGAAGCCCCCTCCACCTCCCACCCATCCC   3224
                       ||||||||||||||||||||||||||||||||||||||||||||||||||
KP025765          6201 tacatatgaccccctccctcttgaagccccctccacctcccacccatccc   6250

ENSOARG000000     3225 ACCCTTCGAGGCTGTCCCAGAGCTGCCGGAGTTGAGGCCCCTGAGCCACA   3274
                       ||||||||||||||||||||||||||||||||||||||||||||||||||
KP025765          6251 acccttcgaggctgtcccagagctgccggagttgaggcccctgagccaca   6300

ENSOARG000000     3275 CAGCGAATCCCCCGGCTACTATTTTACACATGGCAGCGTGTCTGCTTCTG   3324
                       ||||||||||||||||||||||||||||||||||||||||||||||||||
KP025765          6301 cagcgaatcccccggctactattttacacatggcagcgtgtctgcttctg   6350

ENSOARG000000     3325 TGCTACTCTCCGTCAATCCCACCCACAGCTGCCCCCGTCTTTCCTACTGA   3374
                       ||||||||||||||||||||||||||||||||||||||||||||||||||
KP025765          6351 tgctactctccgtcaatcccacccacagctgcccccgtctttcctactga   6400

ENSOARG000000     3375 TGCCCAGCTCAGGTCCTAAGGCAGCTCACCAGCGACTACACTATAGGATT   3424
                       ||||||||||||||||||||||||||||||||||||||||||||||||||
KP025765          6401 tgcccagctcaggtcctaaggcagctcaccagcgactacactataggatt   6450

ENSOARG000000     3425 TGGCAAGTTCGTGGACAAAGTCAGCGTCCCTCAGACGGACATGAGGCCTG   3474
                       ||||||||||||||||||||||||||||||||||||||||||||||||||
KP025765          6451 tggcaagttcgtggacaaagtcagcgtccctcagacggacatgaggcctg   6500

ENSOARG000000     3475 AGAAGTGAGTGATCTACCAAGTAGACGGGGGTCCTGGCCAGGTGGCCCAC   3524
                       ||||||||||||||||||||||||||||||||||||||||||||||||||
KP025765          6501 agaagtgagtgatctaccaagtagacgggggtcctggccaggtggcccac   6550

ENSOARG000000     3525 CTCCTCTCCACCCAGGCACTTTGAAAATCTTGGGGTTACACTGAGCCAAG   3574
                       ||||||||||||||||||||||||||||||||||||||||||||||||||
KP025765          6551 ctcctctccacccaggcactttgaaaatcttggggttacactgagccaag   6600

ENSOARG000000     3575 GGGTGGTCCGCAAGGAGGGTGGGCGCGGGGCATGGGGGTCTTCTTCCCTG   3624
                       ||||||||||||||||||||||||||||||||||||||||||||||||||
KP025765          6601 gggtggtccgcaaggagggtgggcgcggggcatgggggtcttcttccctg   6650

ENSOARG000000     3625 ACTTTCTCTCTCCGTGATGCTCCCCTTCCCACCTTCCCAGGCTGAAGGAA   3674
                       ||||||||||||||||||||||||||||||||||||||||||||||||||
KP025765          6651 actttctctctccgtgatgctccccttcccaccttcccaggctgaaggaa   6700

ENSOARG000000     3675 CCCTGGCCCAACAGTGATCCCCCCTTCTCCTTCAAGAATGTCATCAGTCT   3724
                       |||||||||||||||||||||||||||||||||||||||||||||||.||
KP025765          6701 ccctggcccaacagtgatccccccttctccttcaagaatgtcatcagcct   6750

ENSOARG000000     3725 GACAGAAGACGTGGAGGAGTTCCGGAACAAGCTGAAGGGGGAGCGCATCT   3774
                       |||.||||||||||||||||||||||||||||||||||||||||||||||
KP025765          6751 gacggaagacgtggaggagttccggaacaagctgaagggggagcgcatct   6800

ENSOARG000000     3775 CAGGCAATCTGGATGCCCCCGAAGGAGGTTTTGATGCCATCCTGCAGACA   3824
                       |||||||.||||||||||||||||||||||||||||||||||||||||||
```

```
KP025765         6801 caggcaacctggatgcccccgaaggaggttttgatgccatcctgcagaca   6850

ENSOARG000000    3825 GCCGTGTGCACCGTGAGTGCGGGGAGGCTCCTTCCGTTCTAGACCAGGGC   3874
                      ||||||||||||||||||||||||||||||||||||||||||||||||||
KP025765         6851 gccgtgtgcaccgtgagtgcggggaggctccttccgttctagaccagggc   6900

ENSOARG000000    3875 TGTGTCAAGAGCTGCCCCCTGGCCTGCTCTGGTGCCAAGCTGGGGCCCCC   3924
                      ||||||||||||||||||||||||||||||||||||||||||||||||||
KP025765         6901 tgtgtcaagagctgccccctggcctgctctggtgccaagctggggccccc   6950

ENSOARG000000    3925 ATGCTCTCAGACAAGCTCAGACCCTCTGGGGGACCCTCAATTCCAATCCA   3974
                      ||||||||||||||||||||||||||||||||||||||||||||||||||
KP025765         6951 atgctctcagacaagctcagaccctctgggggaccctcaattccaatcca   7000

ENSOARG000000    3975 AGTTTAGAGGTGTTGGGGCCTGAGCAGTCTTACCCTGCTTTTGTATTGGT   4024
                      ||||||||||||||||||||||||||||||||||||||||||||||||||
KP025765         7001 agtttagaggtgttggggcctgagcagtcttaccctgcttttgtattggt   7050

ENSOARG000000    4025 CACCAAGGTGGACCTGAGCCTCGGCGTTCTGGAATCTTCAGTGCCCCGAG   4074
                      ||||||||||||||||||||||||||||||||||||||||||||||||||
KP025765         7051 caccaaggtggacctgagcctcggcgttctggaatcttcagtgccccgag   7100

ENSOARG000000    4075 CCCCAAGCTCCCTGCTAATACCCATCTCCAGCTGTGCTAGAAGAGGGCAC   4124
                      ||||||||||||||||||||||||||||||||||||||||||||||||||
KP025765         7101 ccccaagctccctgctaatacccatctccagctgtgctagaagagggcac   7150

ENSOARG000000    4125 TTTTTACGTGCACATCAATTTTTTCATTTAAAAAATAATGCACTGATGTT   4174
                      ||||||||||||||||||||||||||||||||||||||||||||||||||
KP025765         7151 tttttacgtgcacatcaattttttcatttaaaaaataatgcactgatgtt   7200

ENSOARG000000    4175 ATAGACAATTTGGAAAACAAAGAACAGAAATTGCCCATAATTCCACCACC   4224
                      |||||||||||||||||||||||||||||||||||||||||||.||||||
KP025765         7201 atagacaatttggaaaacaaagaacagaaattgcccataattctaccacc   7250

ENSOARG000000    4225 ATCACAGAGACAGCTGCCTGATACCCCGGCAGCGTGTACTGTTCCCTTGT   4274
                      ||||||||||||||||||||||||||||||||||||||||||||||||||
KP025765         7251 atcacagagacagctgcctgataccccggcagcgtgtactgttcccttgt   7300

ENSOARG000000    4275 GTAGTCGAAATCAGTGCACGTTATATTCTGCCCTTCTTTCCCCCCAACCT   4324
                      ||||||||||||||||||||||||||||||||||||||||||||||||||
KP025765         7301 gtagtcgaaatcagtgcacgttatattctgcccttctttccccccaacct   7350

ENSOARG000000    4325 ATTGCTTTTATAAACATGGTTTGCAAAACCACCCTATATATTTCTCAAAC   4374
                      ||||||||||||||||||||||||||||||||||||||||||||||||||
KP025765         7351 attgcttttataaacatggtttgcaaaaccaccctatatatttctcaaac   7400

ENSOARG000000    4375 AAATGGCCATGCGCTGCTGTCCCTGTTTTAAGTCTCTTGTTGAACGTTGG   4424
                      ||||||||||||||||||||||||||||||||||||||||||||||||||
KP025765         7401 aaatggccatgcgctgctgtccctgttttaagtctcttgttgaacgttgg   7450

ENSOARG000000    4425 CGTGGTGTCCGTTTTTCTATGCAGTTGAGAGATGCTTGCAGAGTGATTGA   4474
                      ||||||||||||||||||||||||||||||||||||||||||||||||||
KP025765         7451 cgtggtgtccgtttttctatgcagttgagagatgcttgcagagtgattga   7500

ENSOARG000000    4475 GACCTTGAGGGGACAGCTGGCTGAAACTGGCTCCCATCTTGCGTGGTGCC   4524
                      ||||||||||||||||||||||||||||||||||||||||||||||||||
KP025765         7501 gaccttgaggggacagctggctgaaactggctcccatcttgcgtggtgcc   7550

ENSOARG000000    4525 CGCTGTGGGCACCATCCCTGCGTCCATTTCCTGCTCCCCATGGCTGGTTT   4574
                      ||||||||||||||||||||||||||||||||||||||||||||||||||
KP025765         7551 cgctgtgggcaccatccctgcgtccatttcctgctccccatggctggttt   7600

ENSOARG000000    4575 CTGTGCCCCCTCCCCACCCCGGTCATCCTCCCGGCGCTGTTTGTCATGAG   4624
                      ||||||||||||||||||||||||||||||||||||||||||||||||||
KP025765         7601 ctgtgccccctccccaccccggtcatcctcccggcgctgtttgtcatgag   7650
```

```
ENSOARG000000   4625 CGTTGCTCACAGAGGCTGTGAATCCCGAGTCCTTGCTGAGCACCGCGTTG   4674
                     ||||||||||||||||||||||||||||||||||||||||||||||||||
KP025765        7651 cgttgctcacagaggctgtgaatcccgagtccttgctgagcaccgcgttg   7700

ENSOARG000000   4675 GGACCCGCCTTCGCTGTGATGGTGCGTGGCGCTGTGACGTTGCCGTCAGC   4724
                     ||||||||||||||||||||||||||||||||||||||||||||||||||
KP025765        7701 ggacccgccttcgctgtgatggtgcgtggcgctgtgacgttgccgtcagc   7750

ENSOARG000000   4725 AGGCTGCCTGCTCCACGGGCACCCACTCCTTGCTCTCCCCAGAGGGACAT   4774
                     ||||||||||||||||||||||||.|||||||||||||||||||||||||
KP025765        7751 aggctgcctgctccacgggcacccccctccttgctctccccagagggacat   7800

ENSOARG000000   4775 CGGCTGGCGCCCTGACAG--------------------------------   4792
                     ||||||||||||||||||
KP025765        7801 cggctggcgccctgacagcacccacttgctggtgttctccaccgagtcgg   7850

ENSOARG000000   4793 --------------------------------------------------   4792

KP025765        7851 ccttccactacgaggccgacggggccaacgtgctggccggcatcatgagg   7900

ENSOARG000000   4793 --------------------------------------------------   4792

KP025765        7901 cgtaacgacgaggagtgccacctggaccccacgggcacctacactcagta   7950

ENSOARG000000   4793 --AGATGCAGGACTACCCGTCGGTGCCCACCCTGGTGCGCCTGCTCGGCC   4840
                       ||||||||||||||||||||||||||||||||||||||||||||||||
KP025765        7951 caagatgcaggactacccgtcggtgcccaccctggtgcgcctgctcggcc   8000

ENSOARG000000   4841 AGCACAACATCATCCCCATCTTCGCCGTCACCAACTACTCCTATAGCTAC   4890
                     ||||||||||||||||||||||||||||||||||||||||||||||||||
KP025765        8001 agcacaacatcatccccatcttcgccgtcaccaactactcctatagctac   8050

ENSOARG000000   4891 TACGAGGTGCGGGGCTGGGCCTCCCTGGGCTCGGGGGAGGGACCCACGGA   4940
                     ||||||||||||||||||||||||||||||||||||||||||||||||||
KP025765        8051 tacgaggtgcggggctgggcctccctgggctcgggggagggacccacgga   8100

ENSOARG000000   4941 ACTTCTCAGACACACCCCCCACCCTCCTTTCCTTCCTTCCGGAAACTTCA   4990
                     ||||||||||||||||||||||||||||||||||||||||||||||||||
KP025765        8101 acttctcagacacacccccccaccctcctttccttccttccggaaacttca   8150

ENSOARG000000   4991 GATGGGCCCTGGTGGGAAGAGGCGGTCATGTGCCCATCCTCTGACCCCAC   5040
                     ||||||||||||||||||||||||||||||||||||||||||||||||||
KP025765        8151 gatgggccctggtgggaagaggcggtcatgtgcccatcctctgaccccac   8200

ENSOARG000000   5041 TCCCCAGCAGGCAGGAGCTGAGAGCGTCCCCCACCCGCCCCCCAAACCCC   5090
                     |||||||||||||||||||||||||||||||||||||     ||||
KP025765        8201 tccccagcaggcaggagctgagagcgtcccccacccg-----caaa----   8241

ENSOARG000000   5091 CCCCCACCAAAGGGCAGGTGAGCCCCAGAGCTCCCGTCTGGATCCGGAGC   5140
                                  ||.||||||||||||||||||||||||||||||||||
KP025765        8242 -------------gcgggtgagccccagagctcccgtctggatccggagc   8278

ENSOARG000000   5141 GATGGCCCCCGTCAGAGGCTGAGCCCCGGGCCCGGCTTTCTCTCCCGCAG   5190
                     ||||||||||||||||||||||||||||||||||||||||||||||||||
KP025765        8279 gatggcccccgtcagaggctgagccccgggcccggctttctctcccgcag   8328

ENSOARG000000   5191 AGGCTGTCGTCCTATTTCCCCGTCTCCTCACTGGGGGTGCTGCAGGAGGA   5240
                     ||||||||||||||||||||||||||||||||||||||||||||||||||
KP025765        8329 aggctgtcgtcctatttccccgtctcctcactgggggtgctgcaggagga   8378

ENSOARG000000   5241 CTCGTCCAACATTGTCGATCTGCTGCAGGAAGCCTTCAACGTGAGGGCCA   5290
                     ||||||||||||||||||||||||||||||||||||||||||||||||||
KP025765        8379 ctcgtccaacattgtcgatctgctgcaggaagccttcaacgtgagggcca   8428
```

```
ENSOARG000000     5291 GTGCGGGCTGCGGGC-----------------------------------     5305
                       |||||||||||||||
KP025765          8429 gtgcgggctgcgggcccgagggtggcgggggtgggcgggagagatggccg   8478

ENSOARG000000     5306 -----CAGAGGATGGGGAAACTGGATGGGAGGGCGGAAGGGCCTCCTGCG   5350
                            |||||||||||||||||||||||||||||||||||||||||||||
KP025765          8479 gaagacagaggatggggaaactggatgggagggcggaagggcctcctgcg   8528

ENSOARG000000     5351 GTCCCTGAGGAGCCCCCCGGGCTGGCCCCAGGCTCCTGCAGGCTCTGCTC   5400
                       ||||||||||||||||||||||||||||||||||||||||||||||||||
KP025765          8529 gtccctgaggagccccccgggctggccccaggctcctgcaggctctgctc   8578

ENSOARG000000     5401 CCATGAGCCCCCGGCACCCAGGCCTCCGTGCAGGTAAGGCTGCAGCTCAG   5450
                       ||||||||||||||||||||||||||||||||||||||||||||||||||
KP025765          8579 ccatgagcccccggcacccaggcctccgtgcaggtaaggctgcagctcag   8628

ENSOARG000000     5451 GCAGCCAGGCCGCAGCGGGCGGGTCTGGGCCCCACCTCCCAGGTCATCGA   5500
                       ||||||||||||||||||||.|||||||||||||||||||||||||||||
KP025765          8629 gcagccaggccgcagcgggcaggtctgggccccacctcccaggtcatcga   8678

ENSOARG000000     5501 TAACCTGCCCCCTTTCAGCGCATCCGCTCCAACCTGGACATTCGGGCCCT   5550
                       ||||||||||||||||||||||||||||||||||||||||||||||||||
KP025765          8679 taacctgccccctttcagcgcatccgctccaacctggacattcgggccct   8728

ENSOARG000000     5551 GGACAGTCCTCGAGGCCTGCGGACAGAGGTCACCTCTAGGATGTTCCAGA   5600
                       ||||||||||||||||||||||||||||||||||||||||||||||||||
KP025765          8729 ggacagtcctcgaggcctgcggacagaggtcacctctaggatgttccaga   8778

ENSOARG000000     5601 AGACGGACACGGGGTCCTTTTTCATCCGGCGAGGGGAAGTGGTATGCCTC   5650
                       ||||||||||||||||||||||||||||||||||||||||||||||||||
KP025765          8779 agacggacacggggtcctttttcatccggcgaggggaagtggtatgcctc   8828

ENSOARG000000     5651 CATGGGGGCCCCGAGAGACGGGAGGATGGGGACAGCACCCTGTGGGGTGG   5700
                       ||||||||||||||||||.|||||||||||||||||||||||||||||||
KP025765          8829 catgggggccccgagagaggggaggatggggacagcaccctgtggggtgg   8878

ENSOARG000000     5701 GGTCTACCCAAACAGAGATCCAGAGCGTGGCCTCCCGAGTCAGACAGATC   5750
                       ||||||||||||||||||||||||||||||||||||||||||||||||||
KP025765          8879 ggtctacccaaacagagatccagagcgtggcctcccgagtcagacagatc   8928

ENSOARG000000     5751 TGGGTTTGAAGCCCCCTGGAGCAGCCTCTTGGCTGAGCTCTGCCCTGCCC   5800
                       ||||||||||||||||||||||||||||||||||||||||||||||||||
KP025765          8929 tgggtttgaagccccctggagcagcctcttggctgagctctgccctgccc   8978

ENSOARG000000     5801 TTGGTTGGTGCTCAGCTGTAAAGCGGAGATCAGTCAGCATGGAGCCCAGG   5850
                       ||||||||||||||||||||||||||||||||||||||||||||||||||
KP025765          8979 ttggttggtgctcagctgtaaagcggagatcagtcagcatggagcccagg   9028

ENSOARG000000     5851 TTGCTCAGCTGAACGAGCGGGCCGAAGGCAGCTCTGCTTACTGTCGGGCT   5900
                       ||||||||||||||||||||||||||||||||||||||||||||||||||
KP025765          9029 ttgctcagctgaacgagcgggccgaaggcagctctgcttactgtcgggct   9078

ENSOARG000000     5901 CTTGTTATCCTGACTCTGCCTGGAGGAAGCGAGTTGGCAGCCCTGAGGAA   5950
                       ||||||||||||||||||||||||||||||||||||||||||||||||||
KP025765          9079 cttgttatcctgactctgcctggaggaagcgagttggcagccctgaggaa   9128

ENSOARG000000     5951 GGACGGGCAAGGAGCTGGGTGGCAGGGGCCAGACACATTTCTGAAGCTTT   6000
                       ||||||||||||||||||||||||||||||||||||||||||||||||||
KP025765          9129 ggacgggcaaggagctgggtggcaggggccagacacatttctgaagcttt   9178

ENSOARG000000     6001 AGGGCTGAGAGCTTTCAGGTAGAACTGCCATCCCAGCTGGTCCAGCTGGG   6050
                       ||||||||||||||||||||||||||||||||||||||||||||||||||
KP025765          9179 agggctgagagctttcaggtagaactgccatcccagctggtccagctggg   9228

ENSOARG000000     6051 CCTGCCTCGGCTAAAAGCGGGGTCCTTGCAGGGCACATACCAAGTGCAGC   6100
```

```
                        ||||||||||||||||||||||||||||||||||||||||||||||||||
KP025765           9229 cctgcctcggctaaaagcggggtccttgcagggcacataccaagtgcagc   9278

ENSOARG000000      6101 TGCGGGCCATCGAGGACTTGGATGGGGCTCATGTGTGCCAGCTGCCGGAG   6150
                        ||||||||||||||||||||||||||||||||||||||||||||||||||
KP025765           9279 tgcgggccatcgaggacttggatggggctcatgtgtgccagctgccggag   9328

ENSOARG000000      6151 GCAGACCAGAAGGGCACCATCCATCTGAAGCCATCCTTCTCCAACGGCCT   6200
                        ||||||||||||||||||||||||||||||||||||||||||||||||||
KP025765           9329 gcagaccagaagggcaccatccatctgaagccatccttctccaacggcct   9378

ENSOARG000000      6201 CCGGATGGATGTGGGCGTCATCTGTGACGTGTGCTCCTGTGAGCTGGTAT   6250
                        ||||||||||||||||||||||||||||||||||||||||||||||||||
KP025765           9379 ccggatggatgtgggcgtcatctgtgacgtgtgctcctgtgagctggtat   9428

ENSOARG000000      6251 GACACAGCCCTGCCCAGGGGGCGCGGGCAGGGAAAGGCTAAGCTGACCAC   6300
                        ||||||||||||||||||||||||||||||||||||||||||||||||||
KP025765           9429 gacacagccctgcccagggggcgcgggcagggaaaggctaagctgaccac   9478

ENSOARG000000      6301 CCGGGGACCCGAGTAACAAAGCAGAGCTCCCTTTTCGGGGTGCTGGCAAG   6350
                        ||||||||||||||||||||||||||||||||||||||||||||||||||
KP025765           9479 ccggggacccgagtaacaaagcagagctcccttttcggggtgctggcaag   9528

ENSOARG000000      6351 CATCTCCTGCCCTTCCCCCACCCACCTCACCACGATTTCATTCCAGCAAA   6400
                        ||||||||||||||||||||||||||||||||||||||||||||||||||
KP025765           9529 catctcctgcccttcccccacccacctcaccacgatttcattccagcaaa   9578

ENSOARG000000      6401 AAGAGGAGCGGTCATCTCGCTGCAGCTTCCACGGGGACTTCATGTGCGGA   6450
                        ||||||||||||||||||||||||||||||||||||||||||||||||||
KP025765           9579 aagaggagcggtcatctcgctgcagcttccacggggacttcatgtgcgga   9628

ENSOARG000000      6451 CACTGTGTGTGCCATGAGGGCTGGTAAGTGTGGAGGGGGTGAAGCCGGCA   6500
                        ||||||||||||||||||||||||||||||||||||||||||||||||||
KP025765           9629 cactgtgtgtgccatgagggctggtaagtgtggagggggtgaagccggca   9678

ENSOARG000000      6501 CCCAGGATGCCAGCAGCTCCCAGTGAAGGGGCCTGCAAGCGCTTGACACT   6550
                        ||||||||||||||||||||||||||||||||||||||||||||||||||
KP025765           9679 cccaggatgccagcagctcccagtgaaggggcctgcaagcgcttgacact   9728

ENSOARG000000      6551 GGGCATACCATGGAGGCAGGCTGAGGAGAGGGTGGCAGCCGGGCATAGAA   6600
                        ||||||||||||||||||||||||||||||||||||||||||||||||||
KP025765           9729 gggcataccatggaggcaggctgaggagagggtggcagccgggcatagaa   9778

ENSOARG000000      6601 AAGGCTGAGAAAGGGGCACATCCAGTTGGTGGATTAAATTCAAAGCACTT   6650
                        ||||||||||||||||||||||||||||||||||||||||||||.|||||
KP025765           9779 aaggctgagaaaggggcacatccagttggtggattaaattcaaaacactt   9828

ENSOARG000000      6651 TGAAAAACATGGGTTTGGGAATTCCCTGATGGTCCAGTGGTTAGGACTTG   6700
                        ||||||||||||||||||||||||||||||||||||||||||||||||||
KP025765           9829 tgaaaaacatgggtttgggaattccctgatggtccagtggttaggacttg   9878

ENSOARG000000      6701 GTGTTCTCATGGCTGGAGCTGTGGGTTCATTCCCTGATCAGGAAACTTAA   6750
                        ||||||||||||||||||||||||||||||||||||||||||||||||||
KP025765           9879 gtgttctcatggctggagctgtgggttcattccctgatcaggaaacttaa   9928

ENSOARG000000      6751 GATCCTGCAAGCTATCCAGTGCAACAACCCCACCCCCTCCTCCCCCACCC   6800
                        ||||.||||||||||||||||||||||||||| |||||||||||||||||
KP025765           9929 gatcttgcaagctatccagtgcaacaacccca-cccctcctcccccaccc   9977

ENSOARG000000      6801 CCACAAAAAAAAAAACAAAAACCCTGGATAATTCTTGAAAAAATACCTTG   6850
                        ||.| |||||||||||||||||||||||||||||||||||||||||||||
KP025765           9978 ccgc-aaaaaaaaaacaaaaaccctggataattcttgaaaaaataccttg  10026

ENSOARG000000      6851 GTATATTGGATTATCTCTGGAAAGTCATTTAAGAATCAGGACGGTGGCCA   6900
                        ||||||||||||||||||||||||||||||||||||||||||||||||||
```

```
KP025765         10027 gtatattggattatctctggaaagtcatttaagaatcaggacggtggcca  10076

ENSOARG000000     6901 TCAGGGTCTTGTGGGTCAGGCAGAGGGGGAAGCTGACTTTGCCCTCCATC   6950
                       ||||||||||||||||||||||||||||||||||||||||||||||||||
KP025765         10077 tcagggtcttgtgggtcaggcagagggggaagctgactttgccctccatc  10126

ENSOARG000000     6951 CCTTTTGAACCTTTAGTATCTGAGCCATCCTGGAGAAGGAAATGGCTACC   7000
                       ||||||||||||||||||||||||||||||||||||||||||||||||||
KP025765         10127 ccttttgaacctttagtatctgagccatcctggagaaggaaatggctacc  10176

ENSOARG000000     7001 CGCTCCAGTATTCTTGCCTGGAAAAATCCCATGGACAAAGGAGCCTGGTG   7050
                       |.||||||||||||||||||||||||||||||||||||||||||||||||
KP025765         10177 cactccagtattcttgcctggaaaaatcccatggacaaaggagcctggtg  10226

ENSOARG000000     7051 GGCTACAGTCCATGGAGGTGCAAAGAGTCGGACACGACTGAGCACACAGC   7100
                       ||||||||||||||||||||||||||||||||||||||||||||||||||
KP025765         10227 ggctacagtccatggaggtgcaaagagtcggacacgactgagcacacagc  10276

ENSOARG000000     7101 ACATTTGAGCCATGGGCCCCTATTCCCTGAGCAGAAGTGAGTCATGGATG   7150
                       ||||||||||||||||||||||||||||||||||||||||||||||||||
KP025765         10277 acatttgagccatgggcccctattccctgagcagaagtgagtcatggatg  10326

ENSOARG000000     7151 AGTGATAGTAAGAGTAAGGGCTTTTCAAGCTTCAAGACACTCAACAGATT   7200
                       ||||||||||||||||||||||||||||||||||||||||||||||||||
KP025765         10327 agtgatagtaagagtaagggcttttcaagcttcaagacactcaacagatt  10376

ENSOARG000000     7201 TGTCGTGACTTGAGGGGACAGAGGGAGGGTCTCAGGGAACTGAGGTTTGA   7250
                       ||||||||||||||||||||||||||||||||||||||||||||||||||
KP025765         10377 tgtcgtgacttgaggggacagagggagggtctcagggaactgaggtttga  10426

ENSOARG000000     7251 GTTCAGTTCAACCACTGCTGTCTGCTTAACTTAGACCCATTTAGAGCCTC   7300
                       ||||||||||||||||||||||||||||||||||||||||||||||||||
KP025765         10427 gttcagttcaaccactgctgtctgcttaacttagacccatttagagcctc  10476

ENSOARG000000     7301 AGTTTCCTCATCTGTAAAATGGGATGGTGAAGGCCCAGCCTTCTTGCTGG   7350
                       ||||||||||||||||||||||||||||||||||||||||||||||||||
KP025765         10477 agtttcctcatctgtaaaatgggatggtgaaggcccagccttcttgctgg  10526

ENSOARG000000     7351 GCAGTGGGGAAGTGACTGGCTCCTCCTGCAGGAGCGGCAAGACCTGTAAC   7400
                       ||||||||||||||||||||||||||||||||||||||||||||||||||
KP025765         10527 gcagtggggaagtgactggctcctcctgcaggagcggcaagacctgtaac  10576

ENSOARG000000     7401 TGCTCCACCGGCTCCCAGAGCGACCTGCAGCCCTGCCGGCGGGAGGGCGA   7450
                       ||||||||||||||||||||||||||||||||||||||||||||||||||
KP025765         10577 tgctccaccggctcccagagcgacctgcagccctgccggcgggagggcga  10626

ENSOARG000000     7451 GGACAAGGTGTGCTCCGGGCGGGGCGAATGCCAGTGTGGCCACTGTGTGT   7500
                       ||||||||||||||||||||||||||||||||||||||||||||||||||
KP025765         10627 ggacaaggtgtgctccgggcggggcgaatgccagtgtggccactgtgtgt  10676

ENSOARG000000     7501 GCTACGGCGAAGGCCGCTACGAGGGTCAGTTCTGTGAATATGACAACTTC   7550
                       ||||||||||||||||||||||||||||||||||||||||||||||||||
KP025765         10677 gctacggcgaaggccgctacgagggtcagttctgtgaatatgacaacttc  10726

ENSOARG000000     7551 CAGTGTCCCCGCACCTCTGGGTTCCTCTGCAATGGTAAGCTGCTGACCTG   7600
                       ||||||||||||||||||||||||||||||||||||||||||||||||||
KP025765         10727 cagtgtccccgcacctctgggttcctctgcaatggtaagctgctgacctg  10776

ENSOARG000000     7601 AGTGGGGTGGCACGGGGCTTTGGGGGTGGCCTGGGTGCCCAGCCCACACC   7650
                       ||||||||||||||||||||||||||||||||||||||||||||||||||
KP025765         10777 agtggggtggcacggggctttgggggtggcctgggtgcccagcccacacc  10826

ENSOARG000000     7651 CTGCCCTGCTGGCTCTCCCATCAGAGCCCAAGTAGGGAGAGCCACACCAG   7700
                       ||||||.|||||||||||||||||||||||||||||||||||||||||||
KP025765         10827 ctgccccgctggctctcccatcagagcccaagtagggagagccacaccag  10876
```

```
ENSOARG000000   7701 GAGTCAGAATCCCCACGTCCAGGCCTAGCCCTCCTCCAGATTCCCTGAGT   7750
                     ||||||||||||||||||||||||||||||||||||||||||||||||||
KP025765       10877 gagtcagaatccccacgtccaggcctagccctcctccagattccctgagt  10926

ENSOARG000000   7751 GCCGCCCCCAAGTCCAGCCCCAACCTGGACCTCTCTCCTCCAAAAACTTG   7800
                     ||||||||||||||||||||||||||||||||||||||||||||||||||
KP025765       10927 gccgcccccaagtccagccccaacctggacctctctcctccaaaaacttg  10976

ENSOARG000000   7801 ATGCCCAAGGCCCCTACCAGCATGACATTACCCTTATGTTTAAGACTCTG   7850
                     ||||||||||||||||||||||||||||||||||||||||||||||||||
KP025765       10977 atgcccaaggcccctaccagcatgacattacccttatgtttaagactctg  11026

ENSOARG000000   7851 GCTTCCCTGAGGTCCTCCAGTCCATGCCCACCTTCTTTTCCACTCTCCCA   7900
                     |||.||||||||||||||||||||||||||||||||||||||||||||||
KP025765       11027 gctcccctgaggtcctccagtccatgcccaccttcttttccactctccca  11076

ENSOARG000000   7901 TCCCACTAGGGTGGGTACTACCCAGCTCGGTCTCAGGGATTGGTGCTATA   7950
                     |||||||| |||||||||||||||||||||||||||||||||||||||||
KP025765       11077 tcccacta-ggtgggtactacccagctcggtctcagggattggtgctata  11125

ENSOARG000000   7951 AGGCATGGTGATCTTTCCTCTCTTGGAAGGTGCTACATCTCTGGCAGGCA   8000
                     ||||||||||||||||||||||||||||||||||||||||||||||||||
KP025765       11126 aggcatggtgatctttcctctcttggaaggtgctacatctctggcaggca  11175

ENSOARG000000   8001 GGGCCTGGGGTAATGCATCCCACAGGGCCCAACACAGTGTGGGTCCAGGC   8050
                     ||||||||||||||||||||||||||||||||||||||||||||||||||
KP025765       11176 gggcctggggtaatgcatcccacagggcccaacacagtgtgggtccaggc  11225

ENSOARG000000   8051 CAGGGGCCTATAGGCTGGCCAGAGCTTCTGGAACTGGGCAAGGGCCACAG   8100
                     ||||||||||||||||||||||||||||||||||||||||||||||||||
KP025765       11226 caggggcctataggctggccagagcttctggaactgggcaagggccacag  11275

ENSOARG000000   8101 AACCCATAGGGGCCACAGGGAAGGACTGACTGGGTAAATGGAGGAGGTTG   8150
                     ||||||||||||||||||||||||||||||||||||||||||||||||||
KP025765       11276 aacccataggggccacagggaaggactgactgggtaaatggaggaggttg  11325

ENSOARG000000   8151 AATGAAAATCCACTCACCAAATACTTATTAAATCCCAACTGTATACTTGC   8200
                     ||||||||||||||||||||||||||||||||||||||||||||||||||
KP025765       11326 aatgaaaatccactcaccaaatacttattaaatcccaactgtatacttgc  11375

ENSOARG000000   8201 CGCTGGGGGTCTACTCTTTCCTTCATTCTTGCTTTTATCTGCCTTCTCTT   8250
                     ||||||||||||||||||||||||||||||||||||||||||||||||||
KP025765       11376 cgctgggggtctactctttccttcattcttgcttttatctgccttctctt  11425

ENSOARG000000   8251 CTCTTTACCTGCCTACCCCACAGTGTACTCAGAGAAATGAAGGGAGAACC   8300
                     ||||||||||||||||||||||||||||||||||||||||||||||||||
KP025765       11426 ctctttacctgcctaccccacagtgtactcagagaaatgaagggagaacc  11475

ENSOARG000000   8301 CAGGATGTCTGGAGAAAGACAGGTATTTAGCACAGGTGGAGCCATGAGAT   8350
                     ||||||||||||||||||||||||||||||||||||||||||||||||||
KP025765       11476 caggatgtctggagaaagacaggtatttagcacaggtggagccatgagat  11525

ENSOARG000000   8351 AGGAAGGGAGGGAGATGCTGCTGGAAAGGAGGCAGGGCCAGAGTGTGAGG   8400
                     ||||||||||||||||||||||||||||||||||||||||||||||||||
KP025765       11526 aggaagggagggagatgctgctggaaaggaggcagggccagagtgtgagg  11575

ENSOARG000000   8401 GGTTTGGATTCCAGGCCAGGATGTACCAGTTTTAGCCTAGTCCACACTTT   8450
                     ||||||||||||||||||||||||||||||||||||||||||||||||||
KP025765       11576 ggtttggattccaggccaggatgtaccagttttagcctagtccacacttt  11625

ENSOARG000000   8451 TTCTTAAAGGGCCAGACATTAAATGTTTTCAGTTTTGTAAGCCAAACCAT   8500
                     ||||||||||||||||||||||||||||||||||||||||||||||||||
KP025765       11626 ttcttaaagggccagacattaaatgttttcagttttgtaagccaaaccat  11675
```

```
ENSOARG000000   8501 TTCTATCACTACTACTCAGCTCTGCCGTTGTAGCAGAAAGCAGCTATAAG   8550
                     ||||||||||||||||||||||||||||||||||||||||||||||||||
KP025765       11676 ttctatcactactactcagctctgccgttgtagcagaaagcagctataag  11725

ENSOARG000000   8551 CAATACGGAAGCAAACGAGTGTGGCTCTGTTCCAGTAACACTTTTGCAAA   8600
                     ||||||||||||||||||||||||||||||||||||.|||||||||||||
KP025765       11726 caatacggaagcaaacgagtgtggctctgttccagtcacacttttgcaaa  11775

ENSOARG000000   8601 AGCAGATGGTGAGCTGGATTTGGCCTGCAGGCTGTTAGTCAACCCCTATT   8650
                     |||||.|||||||||||||||||||||||||||||||.||||||||||||
KP025765       11776 agcaggtggtgagctggatttggcctgcaggctgttattcaacccctatt  11825

ENSOARG000000   8651 GTAGCAATGATATTGGACTTTATCCTTTGGGCAAAGGGAATCATTGGAGG   8700
                     ||||||||||||||||||||||||||||||||||||||||||||||||||
KP025765       11826 gtagcaatgatattggactttatcctttgggcaaagggaatcattggagg  11875

ENSOARG000000   8701 TTTTAATTTCATTTTCTCTTTATATGTTAACTTTGTATCCAGCCACCTTG   8750
                     ||||||||||||||||||||||||||||||||||||||||||||||||||
KP025765       11876 ttttaatttcattttctctttatatgttaactttgtatccagccaccttg  11925

ENSOARG000000   8751 CTCAACTAACTTATGAAACCTAGTAACTTATCTATAGATTCTTCTTGCTA   8800
                     ||||||||||||||||||||||||||||||||||||||||||||||||||
KP025765       11926 ctcaactaacttatgaaacctagtaacttatctatagattcttcttgcta  11975

ENSOARG000000   8801 TTTGGGGACATTTCTGATACTGGCAAATGACAGTTTTTTGTTTCTTACTT   8850
                     ||||||||||||||||||||||||||||||||||||||||||||||||||
KP025765       11976 tttggggacatttctgatactggcaaatgacagtttttttgtttcttactt  12025

ENSOARG000000   8851 TCCAATAGGTATGTTTTATTTCATTTTCTTACCTTATTGCATTGGCCAGG   8900
                     ||||||||||||||||||||||.|||||||||||||||||||||||||||
KP025765       12026 tccaataggtatgttttatttcgttttcttaccttattgcattggccagg  12075

ENSOARG000000   8901 ACTCATCAGAGGTTCTTAAACAGGGGAGACATGTGTCTGGGTTTGTGTTT   8950
                     ||||||||||||||||||||||||||||||||||||||||||||||||||
KP025765       12076 actcatcagaggttcttaaacaggggagacatgtgtctgggtttgtgttt  12125

ENSOARG000000   8951 TAGGGTGATCACTCCATCTGCAGAGTGGAAGGTAGAGGGCAGAATAGAGC   9000
                     |||||||||||||||||||||||||||||||||||.||||||||||||||
KP025765       12126 tagggtgatcactccatctgcagagtggaaggtagggggcagaatagagc  12175

ENSOARG000000   9001 TGGAAAAATGGAGAGAAGGGAACTCCAGGAAAGAGAGGAAGAGATGACTC   9050
                     ||||||||||||||||||||||||||||||||||||||||||||||||||
KP025765       12176 tggaaaaatggagagaagggaactccaggaaagagaggaagagatgactc  12225

ENSOARG000000   9051 TGCCTGTGGCTGGAGATAGCCTAGCTCCCAAGGCAGCTCCGCCGGGTGGG   9100
                     ||||||||||||||||||||||||||||||||||||||||||||||||||
KP025765       12226 tgcctgtggctggagatagcctagctcccaaggcagctccgccgggtggg  12275

ENSOARG000000   9101 CAGGCTGGACTGCAGGTGCAAATGTGCCGCTCCTCTCTCGCCTTCCCTTC   9150
                     ||||||||||||||||||||||||||||||||||||||||||||||||||
KP025765       12276 caggctggactgcaggtgcaaatgtgccgctcctctctcgccttcccttc  12325

ENSOARG000000   9151 TTTGCAGACCGGGGACGCTGCTCCATGGGCCAGTGCGTGTGTGAGCCTGG   9200
                     ||||||||||||||||||||||||||||||||||||||||||||||||||
KP025765       12326 tttgcagaccggggacgctgctccatgggccagtgcgtgtgtgagcctgg  12375

ENSOARG000000   9201 CTGGACAGGCCTGAGCTGTGACTGTCCTCTCAGCAACGCCACCTGCATCG   9250
                     ||||||||||||||||||||||||||||||||||||||||||||||||||
KP025765       12376 ctggacaggcctgagctgtgactgtcctctcagcaacgccacctgcatcg  12425

ENSOARG000000   9251 ACAGTAACGGGGTAGGCCTGGGCATGCGGCAGGCAGTGGGGGACAGGGGG   9300
                     ||||||||||||||||||||||||||||||||||||||||||||||||||
KP025765       12426 acagtaacggggtaggcctgggcatgcggcaggcagtgggggacaggggg  12475

ENSOARG000000   9301 CGGGCCCCCCTCTCAGACAGGGATGGGGGCGCAGCTGGCTCACGGGGGCC   9350
```

```
                            ||||||||||||||||||||||||||||||||||||||||||||||||||
KP025765           12476 cgggcccccctctcagacagggatgggggcgcagctggctcacgggggcc  12525

ENSOARG000000       9351 CCCTTTTACCTCAGGGCCTCTGTAACGGACGTGGCTACTGCGAGTGCGGC   9400
                         ||||||||||||||||||||||||||||||||||||||||||||||||||
KP025765           12526 ccctttttacctcagggcctctgtaacggacgtggctactgcgagtgcggc  12575

ENSOARG000000       9401 CGTTGCCACTGCAACCAACAGTCACTCTACACGGACACCGTCTGCGAGAT   9450
                         ||||||||||||||||||||||||||||||||||||||||||||||||||
KP025765           12576 cgttgccactgcaaccaacagtcactctacacggacaccgtctgcgagat  12625

ENSOARG000000       9451 CAACTACTCGGCGGTGAGGCCGGACTCGCTGGGTGTGGGTGCGGGGACCC   9500
                         ||||||||||||||||||||||||||||||||||||||||||||||||||
KP025765           12626 caactactcggcggtgaggccggactcgctgggtgtgggtgcggggaccc  12675

ENSOARG000000       9501 ATTGCCATGGGGGCTGCCCAAGGGGGTGAGGTTGCGGGAGCGGGCCTGGC   9550
                         ||||||||||||||||||||||||||||||||||||||||||||||||||
KP025765           12676 attgccatgggggctgcccaagggggtgaggttgcgggagcgggcctggc  12725

ENSOARG000000       9551 AGGGGCCACCCAGCACAGTGCCCGTCTGCCCCTCCTCCTCGCCCACCCAG   9600
                         ||||||||||||||||||||||||||||||||||||||||||||||||||
KP025765           12726 aggggccacccagcacagtgcccgtctgcccctcctcctcgcccacccag  12775

ENSOARG000000       9601 ATCCGCCTGGGCCTCTGTGAGGACCTGCGCTCCTGCGTGCAGTGCCAGGC   9650
                         ||||||||||||||||||||||||||||||||||||||||||||||||||
KP025765           12776 atccgcctgggcctctgtgaggacctgcgctcctgcgtgcagtgccaggc  12825

ENSOARG000000       9651 CTGGGGCACTGGTGAAAAGAAGGGGCGCACGTGCGAGGAGTGCAGCTTCA   9700
                         ||||||||||||||||||||||||||||||||||||||||||||||||||
KP025765           12826 ctggggcactggtgaaaagaaggggcgcacgtgcgaggagtgcagcttca  12875

ENSOARG000000       9701 AGGTCAAGATGGTGGATGAGCTTAAGAAAGGTACAGGGCAGGCAAGAGAA   9750
                         ||||||||||||||||||||||||||||||||||||||||||||||||||
KP025765           12876 aggtcaagatggtggatgagcttaagaaaggtacagggcaggcaagagaa  12925

ENSOARG000000       9751 CTAGGCAAGGGGAGACCTCTGAGAGGTGGGGGTGTCGCTCATCGGGGCGG   9800
                         ||||||||||||||||||||||||||||||||||||||||||||||||||
KP025765           12926 ctaggcaaggggagacctctgagaggtgggggtgtcgctcatcggggcgg  12975

ENSOARG000000       9801 GGCTGTAGAGCTGGGTAGGGGGCTCATAGTCCAAAGTGAGGACAGGCAGT   9850
                         ||||||||||||||||||||||||||||||||||||||||||||||||||
KP025765           12976 ggctgtagagctgggtagggggctcatagtccaaagtgaggacaggcagt  13025

ENSOARG000000       9851 CCGCAAAGAGGGGTTCCAGGCCTGGGGAGTGGTCACAGAATCCAGACCGG   9900
                         ||||||||||||||||||||||||||||||||||||||||||||||||||
KP025765           13026 ccgcaaagaggggttccaggcctggggagtggtcacagaatccagaccgg  13075

ENSOARG000000       9901 GCACACAGACCCAGGGAGGGAACGCACAGTCTGGCCAGGGCACCCAGTCC   9950
                         ||||||||||||||||||||||||||||||||||||||||||||||||||
KP025765           13076 gcacacagacccagggagggaacgcacagtctggccagggcacccagtcc  13125

ENSOARG000000       9951 TGGGGAGGGGGCCATGATCATAGCAAAGGTGCAAAAATGGGCCCTGGGGC  10000
                         ||||||||||||||||||||||||||||||||||||||||||||||||||
KP025765           13126 tggggagggggccatgatcatagcaaaggtgcaaaaatgggccctggggc  13175

ENSOARG000000      10001 ACACAGCCTCCTTTCCTCTGGTCAGAAAAATGTTCTAGAAGCCCAGCTCC  10050
                         ||||||||||||||||||||||||||||||||||||||||||||||||||
KP025765           13176 acacagcctcctttcctctggtcagaaaaatgttctagaagcccagctcc  13225

ENSOARG000000      10051 TGAGTGATGCCCAGGACAGGCACAGATCTGCAGGCTTTGA-GGT-CTGGG  10098
                         |||||||||||||||||||||||||||||||||||||||| ||| |||||
KP025765           13226 tgagtgatgcccaggacaggcacagatctgcaggctttgacggtcctggg  13275

ENSOARG000000      10099 CAGGGCAGCCCCCACTTTACCCCCCAACCCCA-CCCCCCATGTGCACAGT  10147
                         |||||||||||||||||||||||||||||||| |||||||||||||||||
```

```
KP025765          13276 cagggcagcccccactttacccccaaccccacccccccatgtgcacagt  13325

ENSOARG000000     10148 CTGCTGGGAACTCACATTCTTCCCCAGGGAGAGGCCTCCTCTCCTCCATG  10197
                        ||||.|||||||||||||||||||||||||||||||||||||||||||||
KP025765          13326 ctgccgggaactcacattcttccccagggagaggcctcctctcctccatg  13375

ENSOARG000000     10198 CCAACCCCGTGATGGGCGAGGTGGGCCCGAGAGCAAGGCCGTGGTGAGGA  10247
                        ||||||||||||||||||||||||||||||||||||||||||||||||||
KP025765          13376 ccaaccccgtgatgggcgaggtgggcccgagagcaaggccgtggtgagga  13425

ENSOARG000000     10248 GCGGACCCCTCCCCCGGCCCTGAGGTGCTCCGGGCTGGGGTCGGGTGCCC  10297
                        |||||||||||.||.|||||||||||||||||||||||.|||.|||||||
KP025765          13426 gcggacccctcgccaggccctgaggtgctccgggctgaggttgggtgccc  13475

ENSOARG000000     10298 TGCTGACCGTCTGTGTCTGCAGCGGAGGAGGTGGTGGAGCACTGCTCCTT  10347
                        ||||||||||||||||||||||||||||||||||||||||||||||||||
KP025765          13476 tgctgaccgtctgtgtctgcagcggaggaggtggtggagcactgctcctt  13525

ENSOARG000000     10348 CCGGGATGAGGATGACGACTGTACCTACAGTTACACCGTGGAGGGTGACA  10397
                        ||||||||||||||||||||||||||||||||||||||||||||||||||
KP025765          13526 ccgggatgaggatgacgactgtacctacagttacaccgtggagggtgaca  13575

ENSOARG000000     10398 GCGCCCCCGGTCCCAACAGCACCGTCCTGGTGCAGAGGAGGAAGGGTGAG  10447
                        |||||||.||.|||||||||||||||||||||||||||||||||||||||
KP025765          13576 gcgcccctgggcccaacagcaccgtcctggtgcagaggaggaagggtgag  13625

ENSOARG000000     10448 CCGGCGGGCTGGCAGGGATGGCCTGGGTGGCCTGTCAGGCCTGCTTCAG  10497
                        ||||||||||||||||||||||.|||||||||||||||||||||||||||
KP025765          13626 ccggcgggctggcagggatgggcatgggtggcctgtcaggcctgcttcag  13675

ENSOARG000000     10498 GGGGATGGCACTGGCTCCCTCCTCCTCCTCTTTCCTCCCTAGAATGCCCC  10547
                        ||||||||||||||||||||||||||||||||||||||||||||||||||
KP025765          13676 ggggatggcactggctccctcctcctcctctttcctccctagaatgcccc  13725

ENSOARG000000     10548 CCGGGCACCTTCTGGTGGCTCATCCCCCTGCTCATCTTCCTCCTTCTGTT  10597
                        ||||||||||||||||||||||||||||||||||||||||||||||||||
KP025765          13726 ccgggcaccttctggtggctcatcccccctgctcatcttcctccttctgtt  13775

ENSOARG000000     10598 CCCGGTCCTGCTGCTGCTGCTCTGCTGGAAGTACTGTGCCTGCTGCAAGG  10647
                        ||||||||||||||||||||||||||||||||||||||||||||||||||
KP025765          13776 cccggtcctgctgctgctgctctgctggaagtactgtgcctgctgcaagg  13825

ENSOARG000000     10648 TGGGGGCTTCCCCGGTCCCGACCCCACCGGGGTCCCTGTAGAGGGCAGGG  10697
                        ||||||||||||||||||||||||||||||||||||||||||||||||||
KP025765          13826 tgggggcttccccggtcccgaccccaccggggtccctgtagagggcaggg  13875

ENSOARG000000     10698 GCCAAGTGAACCCACACATTCCTCCCAGCTCTGGTGCCATCCCCACCCCA  10747
                        ||||||||||||||||||||||||||||||||||||||||||||||||||
KP025765          13876 gccaagtgaacccacacattcctcccagctctggtgccatccccacccca  13925

ENSOARG000000     10748 GCCCCTCGCCTCTGCCAGCCACCAGTGGCCGAATTCCTGGGAGTCCCTGG  10797
                        ||||||||||||||||||||||||||||||||||||||||||||||||||
KP025765          13926 gcccctcgcctctgccagccaccagtggccgaattcctgggagtccctgg  13975

ENSOARG000000     10798 CCCAGGGGTCAGCCG--------------CAACAGCCAACAGGAAGGAAG  10833
                        |||.|||||||||||              |||||||||||||||||||||
KP025765          13976 cccgggggtcagccgcatgcaaagggtcacaacagccaacaggaaggaag  14025

ENSOARG000000     10834 GGCTCTGCCCACCAGGAGCTGGCCATCTCCCTGGGAGAAAAGACTGGAGA  10883
                        ||||||||||||||||||||||||||||||||||||||||||||||||||
KP025765          14026 ggctctgcccaccaggagctggccatctccctgggagaaaagactggaga  14075

ENSOARG000000     10884 CAGGAGAGTGCAGAGTATGGCCCGGCGTGGAATACTGAAGGGGGCAGAGG  10933
                        ||||||||||||||||||||||||||||||||||||||||||||||||||
KP025765          14076 caggagagtgcagagtatggcccggcgtggaatactgaagggggcagagg  14125
```

```
ENSOARG000000  10934 CGCCCATACAGCAGCCCCCAGGGTCGTACTCAAGTGTGTTACAGGAAAAA  10983
                     ||||||||||||||||||||||||||||||||||||||||||||||||||
KP025765       14126 cgcccatacagcagcccccagggtcgtactcaagtgtgttacaggaaaaa  14175

ENSOARG000000  10984 GCGTTCTGTGATCAAATACTTTTGAGAAAGCAGTTTCTCCTCTGCAGGGC  11033
                     |||||||||||||||||||||||||||||.|.||||||||||||||||||
KP025765       14176 gcgttctgtgatcaaatacttttgagaaaacggtttctcctctgcagggc  14225

ENSOARG000000  11034 TTCTCGGAGCCTTCGAGCTAGTTGTTTGCATAATGAAACTACATGGGGAA  11083
                     |||||||||||||.||||||||||||||||||||||||||||||||.|||
KP025765       14226 ttctcggagcctttgagctagttgtttgcataatgaaactacatggagaa  14275

ENSOARG000000  11084 GGAATTCAGTGCTTCCCGAAGGGGTTTGATCACAGAACCTTTTACTTTCC  11133
                     ||||||||||||||||||||||||||||||||||||||||||||||||||
KP025765       14276 ggaattcagtgcttcccgaaggggtttgatcacagaaccttttactttcc  14325

ENSOARG000000  11134 GGAGTAGATAGTGGGACCAGAGTCCCAGGGCGTGCATTTTGGGTACCCTC  11183
                     ||||||||||||||||||||||||||||||||||||||||||||||||||
KP025765       14326 ggagtagatagtgggaccagagtcccagggcgtgcattttgggtaccctc  14375

ENSOARG000000  11184 TGCCCCTGTTCGCCTCTGTGCCCACACCCAGGCCCCTGTAACTGCTTGGA  11233
                     ||||||||||||||||||||||||||||||||||||||||||||||||||
KP025765       14376 tgcccctgttcgcctctgtgcccacacccaggcccctgtaactgcttgga  14425

ENSOARG000000  11234 TATCCTGATGTCTGGGGAAGGCACTGCTGACCCCTCTGGACCACTGGCGT  11283
                     ||||||||||||||||||||||||||||||||||||||||||||||||||
KP025765       14426 tatcctgatgtctggggaaggcactgctgacccctctggaccactggcgt  14475

ENSOARG000000  11284 GGGTGGGAGGCAGTAGCCGGAGGGACTTCCCTGGGCCAGTATGGCCGTCT  11333
                     ||||||||||||||||||||||||||||||||||||||||.|||||||||
KP025765       14476 gggtgggaggcagtagccggagggacttccctgggccagtgtggccgtct  14525

ENSOARG000000  11334 CAGCTCTTATCCCCACCCTCCTTGCCTCCTAGGCCTGTCTGGCCCTTCTC  11383
                     ||||||||||||||||||||||||||||||||||||||||||||||||||
KP025765       14526 cagctcttatccccaccctccttgcctcctaggcctgtctggcccttctc  14575

ENSOARG000000  11384 CCTTGCTGCAACCAAGGTACCGGCCTGGCCTCGGCGGGGGTGGTGGGGCT  11433
                     ||||||||||||||||||||||||||||||||||||||||||||||||||
KP025765       14576 ccttgctgcaaccaaggtaccggcctggcctcggcgggggtggtggggct  14625

ENSOARG000000  11434 CTGACAGCTCCTCCTTTGGCGGGGGTGGAGGGGAAGCAAGGCGCCAAGTC  11483
                     ||||||||||||||||||||||||||||||||||||||||||||||||||
KP025765       14626 ctgacagctcctcctttggcgggggtggaggggaagcaaggcgccaagtc  14675

ENSOARG000000  11484 TGGGACAGAGGCCGGGGACCGTGGCGGGGGTCCCACCTGTGGGGTGTGGT  11533
                     ||||||||||||||||||||||||.|||||||||||||||||||||||||
KP025765       14676 tgggacagaggccggggaccgtggtgggggtcccacctgtggggtgtggt  14725

ENSOARG000000  11534 GGGGGAGGGTCTAGAAAGCAGCCCCTCAGCCCCGTACTGAGGCCCAGGTC  11583
                     ||||||||||||||||||||||||||||||||||||||||||||||||||
KP025765       14726 gggggagggtctagaaagcagcccctcagccccgtactgaggcccaggtc  14775

ENSOARG000000  11584 CCACAGGTCACATGGTGGGCTTCAAGGAAGACCACTACATGCTGCGAGAG  11633
                     ||||||||||||||||||||||||||||||||||||||||||||||||||
KP025765       14776 ccacaggtcacatggtgggcttcaaggaagaccactacatgctgcgagag  14825

ENSOARG000000  11634 AACCTGATGGCCTCGGACCACCTGGACACGCCCTTGCTGCGCAGTGGGAA  11683
                     ||||||||||||||||||||||||||||||||||||||||||||.|||||
KP025765       14826 aacctgatggcctcggaccacctggacacgcccttgctgcgcagcgggaa  14875

ENSOARG000000  11684 CCTCAAGGGGCGCGACACAGTCCGATGGAAGATCACCAACAATGTGCAGC  11733
                     ||||||||||||||||||||||||||||||||||||||||||||||||||
KP025765       14876 cctcaaggggcgcgacacagtccgatggaagatcaccaacaatgtgcagc  14925
```

```
ENSOARG000000  11734 GGCCGGGCTTCGCCTCGCACGCTGCTGGCATCAACCCCTCGGAGCTGGGT  11783
                     ||||||||||||||||||||||||||||||||||||||||||||||||||
KP025765       14926 ggccgggcttcgcctcgcacgctgctggcatcaacccctcggagctgggt  14975

ENSOARG000000  11784 GAGGGCCGGGGCTGGGCACCGCGGCTCCTGGCGCTGCCCTTGGGGCCCGG  11833
                     ||||||||||||||||||||||||||||||||||||||||||||||||||
KP025765       14976 gagggccggggctgggcaccgcggctcctggcgctgcccttggggcccgg  15025

ENSOARG000000  11834 GGCACGTTCACCACTGCTCGCTGCTCCGGGATCCAACATTCCTCTTTTCT  11883
                     ||||||||||||||||||||||||||||||||||||||||||||||||||
KP025765       15026 ggcacgttcaccactgctcgctgctccgggatccaacattcctcttttct  15075

ENSOARG000000  11884 CTGGGTATCAGGTGCAGGCACATGCCTGTTACCTCCTACCCTTCCTGAGG  11933
                     |||||||||.||||||||||||||||||||||||||||||||||||||||
KP025765       15076 ctgggtatcgggtgcaggcacatgcctgttacctcctacccttcctgagg  15125

ENSOARG000000  11934 GGTACATGCCCCCAGTGCCCCCCAGCCTGAGCACGGCTTCTGGAGGGGAC  11983
                     ||||||||||||||||||||||||||||||||||||||||||||||||||
KP025765       15126 ggtacatgcccccagtgccccccagcctgagcacggcttctggaggggac  15175

ENSOARG000000  11984 TTCTGCAGTGCCCACCATGGTGGGACTGACGATCCCCTCTTCTCGCCTGC  12033
                     ||||||||||||||||||||||||||||||||||||||||||||||||||
KP025765       15176 ttctgcagtgcccaccatggtgggactgacgatcccctcttctcgcctgc  15225

ENSOARG000000  12034 CCTAGTGCCCTACGGACTGTCCCTGCG----GCCCCCCTCTGCACCGAAA  12079
                     |||||||||||||||||||||||||||    ||||.||||||||||||.|
KP025765       15226 cctagtgccctacggactgtccctgcggcttgcccgcctctgcaccgaga  15275

ENSOARG000000  12080 ACCGGCTGAAGCCTGGCACACGAGAGTGTGACCAGCTGCGCCAGGAGGTG  12129
                     |||.||||||||||||||||||||||||||||||||||||||||||||||
KP025765       15276 acctgctgaagcctggcacacgagagtgtgaccagctgcgccaggaggtg  15325

ENSOARG000000  12130 GAAGAGAATGTAAGGAGCTGGTCCCCGGCAGCGGGTGGCTCTGGGCCGGG  12179
                     ||||||||||||||||||||||||||||||||||||||||||||||.|||
KP025765       15326 gaagagaatgtaaggagctggtccccggcagcgggtggctctgggctggg  15375

ENSOARG000000  12180 TGTGAGACGGGTGGTG----------------------------------  12195
                     ||||||||||||||||
KP025765       15376 tgtgagacgggtggtgggagggcagagggaagctgccgtggcgcctggat  15425

ENSOARG000000  12196 ----------------------------------------TGGCCGGGGG  12205
                                                             ||||||||||
KP025765       15426 gtaaccgggtgggagagaagggcttcctcggacatgtgcatggccggggg  15475

ENSOARG000000  12206 GTCACCCTGGGATCTGCCTCCTGTGAATGCTTCCAGAAGGGTAGAGCCAC  12255
                     ||||||||||||||||||||||||||||||||||||||||||||||||||
KP025765       15476 gtcaccctgggatctgcctcctgtgaatgcttccagaagggtagagccac  15525

ENSOARG000000  12256 TGCTCATGCCTGTGGCCATGCCCCCAGCCACGCCCAGGAGGTCCTCTGGG  12305
                     ||||||||||||||||||||||||||||||||||||||||||||||||||
KP025765       15526 tgctcatgcctgtggccatgcccccagccacgcccaggaggtcctctggg  15575

ENSOARG000000  12306 TGCAGGGGCTCCCCCAGGGCCCCAGCCTCACAGCCCTCCCTTGCCCGGCA  12355
                     ||||||||||||||||||||||||||||||||||||||||||||||||||
KP025765       15576 tgcaggggctcccccagggccccagcctcacagccctcccttgcccggca  15625

ENSOARG000000  12356 GCTGAACGAGGTGTACCGACAAATCTCAGGCGCTCACAGCCTTCAGCAGA  12405
                     ||||||.|||||||||||||||||||||||||||||||||||||||||||
KP025765       15626 gctgaatgaggtgtaccgacaaatctcaggcgctcacagccttcagcaga  15675

ENSOARG000000  12406 CCAAGTTCCGGTGGGTCCTGGGCGCCCGGGGTGGGGTTAGGGGTGGGGCA  12455
                     ||||||||||||||||||||||||||||||||||||||||||||||||||
KP025765       15676 ccaagttccggtgggtcctgggcgcccggggtggggttaggggtggggca  15725

ENSOARG000000  12456 TTGCTCCCATAGCTGACGCCAGGGGATTTCTCCCAAGCAGACAATCCCTG  12505
```

```
                           ||||||||||||||||||||||||||||||||||||||||||||||||||
KP025765          15726 ttgctcccatagctgacgccaggggatttctcccaagcagacaatccctg  15775

ENSOARG000000     12506 CCCCCTAGGGACCTTCTGGCTCACGGTCTTCACTTTGTCCCCCACAGGCA  12555
                        ||||||||||||||||||||||||||||||||||||||||||||||||||
KP025765          15776 ccccctagggaccttctggctcacggtcttcactttgtcccccacaggca  15825

ENSOARG000000     12556 GCAGCCCAACGCTGGGAAAAAGTGAGTTGAAGACACATCGGTGGGCAGTA  12605
                        ||||||||||||||||||||||||||||||||||||||||||||||||||
KP025765          15826 gcagcccaacgctgggaaaaagtgagttgaagacacatcggtgggcagta  15875

ENSOARG000000     12606 CCAGCCCCAAGTCCCACCTCCCCCATCCCCACCTCCCCCATCCCCACCTC  12655
                        |||||||||||||||||||||||||||||.||.|         ||..|||
KP025765          15876 ccagccccaagtcccacctcccccatcccaactt---------ccgtctc  15916

ENSOARG000000     12656 CCCCATCCCAACTTCCGCC-------------------TCCA-----GTC  12681
                        |..||||      ||||||                   ||||     |||
KP025765          15917 catcatc------tccgccccatgggagaggagggctgtccaggtctgtc  15960

ENSOARG000000     12682 CTGGGGTAGGACCTTCACTAAACACATACCCTTATGAAGCCCTCACCGAG  12731
                        |||||||||||||||||||||||||||||||.||||||||||||||||||
KP025765          15961 ctggggtaggaccttcactaaacacatacccgtatgaagccctcaccgag  16010

ENSOARG000000     12732 AGCCAGCCCCTGCATGAGAACAAGGAACCCACAGGGAAACGAGCCAACCC  12781
                        ||||||||||||.|||||||||||||||||||||||||||||||||||||
KP025765          16011 agccagcccctgtatgagaacaaggaacccacagggaaacgagccaaccc  16060

ENSOARG000000     12782 AGTCCCTGGCCTCACACAGGCAATAGCCCCCACCCAGTACCAGGGATGGC  12831
                        |||||||||||||||||.||||||||||||||||||||||||||||.|||
KP025765          16061 agtccctggcctcacacgggcaatagcccccacccagtaccagggacggc  16110

ENSOARG000000     12832 CGGTGCCCAGAGCCAGACCTCCTCTGAGGCCAAAAGCCTTGATCCTGGAG  12881
                        ||||||||||||||||||||||||||||||||||||||||||||||||||
KP025765          16111 cggtgcccagagccagacctcctctgaggccaaaagccttgatcctggag  16160

ENSOARG000000     12882 GCACCGCTGGTTACTAACCAGATGGTCCAGTGGGTGACACGCTTCTTTCC  12931
                        |||||||||||||||||||||||||||||||||.||||||||||||||||
KP025765          16161 gcaccgctggttactaaccagatggtccagtggatgacacgcttctttcc  16210

ENSOARG000000     12932 CAGTTCCCACCCCGAGTCCCAGACCCCCCAGGGAAGAGTGGGGTGGGGTT  12981
                        ||||||||||||||||||||||||||||||||||||||||||||||||||
KP025765          16211 cagttcccaccccgagtcccagaccccccagggaagagtggggtggggtt  16260

ENSOARG000000     12982 CAATACCAAGAAGACAAAACCCAGCTCTTACCGGGCACAGCGCTGTCAGT  13031
                        ||||||||||||||||||||||||||||||||||||||||||||||||||
KP025765          16261 caataccaagaagacaaaacccagctcttaccgggcacagcgctgtcagt  16310

ENSOARG000000     13032 AAGAACCATTTAGACTGGCACTTTGAGTGCTGACCACCGGCCAGACCTTG  13081
                        ||||||||||||||||||||||||||||||||||||||||||||||||||
KP025765          16311 aagaaccatttagactggcactttgagtgctgaccaccggccagaccttg  16360

ENSOARG000000     13082 TTTTAAGGACTCGTGTGTGTGGTTCTTACTCAAGTGGGTATCAGTGCTAT  13131
                        ||||||||||.|||||||||||||||||||||||||||||||||||||||
KP025765          16361 ttttaaggacgcgtgtgtgtggttcttactcaagtgggtatcagtgctat  16410

ENSOARG000000     13132 GCCCTTTGTATAGTTGAGGAAACTGAGGATCAGAGAAGTTAAGTAACTTA  13181
                        ||||||||||||||||||||||||||||||||||||||||||||||||||
KP025765          16411 gccctttgtatagttgaggaaactgaggatcagagaagttaagtaactta  16460

ENSOARG000000     13182 CCCAAGGTTACCTAGCCATTAAGATAGAACCAGGGGAATTCCCTGGTGGT  13231
                        ||||||||||||||||||||||||||||||||||||||||||||||||||
KP025765          16461 cccaaggttacctagccattaagatagaaccaggggaattccctggtggt  16510

ENSOARG000000     13232 TCAGCAGTTAAAGCTTCCTAGGTGGCTCAGTGGTAAAGAACCTGCCTAGT  13281
                        ||||||||||||||||||||||||||||||||||||||||||||||||||
```

```
KP025765          16511 tcagcagttaaagcttcctaggtggctcagtggtaaagaacctgcctagt  16560

ENSOARG000000     13282 GCAAAAGACAAAAGACACGGGTTCAGTCCCTGGGTCTGGAAGATTTCCTG  13331
                        ||||||||||||||||||||||||||||||||||||||||||||||||||
KP025765          16561 gcaaaagacaaaagacacgggttcagtccctgggtctggaagatttcctg  16610

ENSOARG000000     13332 GAGTAGGAATGGCAACCCACTCCAGTATTCTTGTATGGAAAATTCCATGG  13381
                        ||||||||||||||||||||||||||||||||||||||||||||||||||
KP025765          16611 gagtaggaatggcaacccactccagtattcttgtatggaaaattccatgg  16660

ENSOARG000000     13382 ACGGAGGAACCAGGTGGGCTACGGTCCATGGGGTCACAAAGAGTCGGACA  13431
                        ||||||||||||||||||||||||||||||||||||||||||||||||||
KP025765          16661 acggaggaaccaggtgggctacggtccatggggtcacaaagagtcggaca  16710

ENSOARG000000     13432 CGATTGAGCTCAACACAGGAGCAACAGTTAAGACTCAGGCTTTGGCTGCC  13481
                        ||||||||||||||||||||||||||||||||||||||||||||||||||
KP025765          16711 cgattgagctcaacacaggagcaacagttaagactcaggctttggctgcc  16760

ENSOARG000000     13482 AGGGACCAGGTTCAATTTCCATATGGTTGGGTGAAAAAAAAAAATTTAAA  13531
                        ||||||||||||||||||||||||||||||||||||||||||.|||||||
KP025765          16761 agggaccaggttcaatttccatatggttgggtgaaaaaaaaacatttaaa  16810

ENSOARG000000     13532 GATGAAGAGCCAGGATTGCAGCCAGGCAGTCTGGCCTGTCCCCTTGGCAT  13581
                        ||||||||||||||||||||||||||||||||||||||||||||||||||
KP025765          16811 gatgaagagccaggattgcagccaggcagtctggcctgtccccttggcat  16860

ENSOARG000000     13582 CTTTGCTGCCCCGCCTCCATGTGTGCGTCATCAGCCCCTCTGGGAGAGGT  13631
                        ||||||||||||||||||||||||||||||||||||||||||||||||||
KP025765          16861 ctttgctgccccgcctccatgtgtgcgtcatcagcccctctgggagaggt  16910

ENSOARG000000     13632 GGGCAGGGAGAGGAGCCTCCTCTCACAGATAAGGACCCTGAGGCTGCAGA  13681
                        ||||||||||||||||||||||||||||||||||||||||||||||||||
KP025765          16911 gggcagggagaggagcctcctctcacagataaggaccctgaggctgcaga  16960

ENSOARG000000     13682 GGGCGGTGGCGGGGCCAGGCCGGTCTCTGCATCTGTCTGGCTCTGAAAAA  13731
                        ||||||||||||||||||||||||||||||||||||||||||||||||||
KP025765          16961 gggcggtggcggggccaggccggtctctgcatctgtctggctctgaaaaa  17010

ENSOARG000000     13732 AACGCTTTTTGTTGCCTGGAATCTTGTGCCCCAATGCAGAGAGGACAGGC  13781
                        ||||||||||||||||||||||||||||||||||||||||||||||||||
KP025765          17011 aacgctttttgttgcctggaatcttgtgccccaatgcagagaggacaggc  17060

ENSOARG000000     13782 AGGAGGGGCAGACAGGGACGCGTGGCAGCCTCCCAGATGGCCTGACACTC  13831
                        ||||||||||||||||||||||||||||||||||||||||||||||||||
KP025765          17061 aggaggggcagacagggacgcgtggcagcctcccagatggcctgacactc  17110

ENSOARG000000     13832 CCCACCCACCCCGCCCCCAGGCAGGACCACACCATTGTGGACACGGTGCT  13881
                        ||||||||||||||||||||||||||||||||||||||||||||||||||
KP025765          17111 cccacccaccccgcccccaggcaggaccacaccattgtggacacggtgct  17160

ENSOARG000000     13882 GATGGCTCCCCGCTCAGCCAAGCAGGCCCTGTTGAAGTTGACAGAGAAGC  13931
                        ||||||||||||||||||||||||||||||||||||||||||||||||||
KP025765          17161 gatggctccccgctcagccaagcaggccctgttgaagttgacagagaagc  17210

ENSOARG000000     13932 ACGTGGAGCAGGGGGCCTTCCATGAGCTCAAGGTGGCCCCCGGCTATTAC  13981
                        ||||||||||||||||||||||||||||||||||||||||||||||||||
KP025765          17211 acgtggagcagggggccttccatgagctcaaggtggcccccggctattac  17260

ENSOARG000000     13982 ACACTCACTGCAGACCAGGGTAGGTGGGCAGGCTCCCTGTGCCCCTCCCT  14031
                        ||||||||||||||||||||||||||||||||||||||||||||||||||
KP025765          17261 acactcactgcagaccagggtaggtgggcaggctccctgtgcccctccct  17310

ENSOARG000000     14032 CTCACCTCCCACCCCCGTGTGGCCCCCTGACCCTCGTCTGATGGCTCCCT  14081
                        |||||||||||||||||||.|||||||||||||.||||||||||||||||
KP025765          17311 ctcacctcccacccccgtgcggccccctgacccccgtctgatggctccct  17360
```

```
ENSOARG000000  14082 ACCAGACGCCCGAGGCATGGTGGAGTTCCAGGAGGGCGTGGAGCTGGTGG  14131
                     ||||||||||||||||||||||||||||||||||||||||||||||||||
KP025765       17361 accagacgcccgaggcatggtggagttccaggagggcgtggagctggtgg  17410

ENSOARG000000  14132 ACGTGCGGGTGCCTCTGTTTATCCGGCCCGAGGATGACGACGAGAAGCAG  14181
                     ||||||||||||||||||||||||||||||||||||||||||||||||||
KP025765       17411 acgtgcgggtgcctctgtttatccggcccgaggatgacgacgagaagcag  17460

ENSOARG000000  14182 CTGCTGGTGGAGGCCATTGATGTGCCCGTGGGCACCGCCACCCTCGGACG  14231
                     ||||||||||||||||||||||||||||||||||||||||||||||||||
KP025765       17461 ctgctggtggaggccattgatgtgcccgtgggcaccgccaccctcggacg  17510

ENSOARG000000  14232 CCGCCTGGTCAATATCACCATCATCAAGGAGCAAGGTCGGTCAGGATGGG  14281
                     ||||||||||||||||||||||||||||||||||||||||||||||||||
KP025765       17511 ccgcctggtcaatatcaccatcatcaaggagcaaggtcggtcaggatggg  17560

ENSOARG000000  14282 GGCCACCGGGGACCCAAAGGGGACTCTGGGACCCTTGTCTCTCCTCACAT  14331
                     ||||||||||||||||||||||||||||||||||||||||||||||||||
KP025765       17561 ggccaccggggacccaaaggggactctgggacccttgtctctcctcacat  17610

ENSOARG000000  14332 CCAAGTGGAAGTGGGACCTGGCCCTGTGGCCTAGGCAAGCAACTCAACCT  14381
                     ||||||||||||||||||||||||||||||||||||||||||||||||||
KP025765       17611 ccaagtggaagtgggacctggccctgtggcctaggcaagcaactcaacct  17660

ENSOARG000000  14382 CTGAGCCTTGGTTTCCCCATCTGCAAAATGGGTATTAGGCTCTTGTGAGG  14431
                     ||||||||||||||||||||||||||||||||||||||||||||||||||
KP025765       17661 ctgagccttggtttccccatctgcaaaatgggtattaggctcttgtgagg  17710

ENSOARG000000  14432 GGGGTGTGGCTATACCTCTCTCATAAGTGTGCAGTGGGGGGCTTCTCTTA  14481
                     ||||||||||||||||||||||||||||||||||||||||||||||||||
KP025765       17711 ggggtgtggctatacctctctcataagtgtgcagtggggggcttctctta  17760

ENSOARG000000  14482 TCATCACCAGAGGGTACTGAGTTTTCCTGGAGCCGTCCCACGACCCTCTC  14531
                     ||||||||||||||||||||||||||||||||||||||||||||||||||
KP025765       17761 tcatcaccagagggtactgagttttcctggagccgtcccacgaccctctc  17810

ENSOARG000000  14532 CTTGTCCTGTGGCCTTTGAACTTGAGACTGAATGGGTCCCCAGGATGCCA  14581
                     ||||||||||||||||||||||||||||||||||||||||||||||||||
KP025765       17811 cttgtcctgtggcctttgaacttgagactgaatgggtccccaggatgcca  17860

ENSOARG000000  14582 GGACATCAGAGCAAGAAGGGACCTGGGGCTTCTTCTGCCCAGGCCTACCT  14631
                     ||||||||||||||||||||||||||||||||||||||||||||||||||
KP025765       17861 ggacatcagagcaagaagggacctggggcttcttctgcccaggcctacct  17910

ENSOARG000000  14632 AGAAGGGCGGGCAAGGCGTCACACCACCGTCACTTACCTTGCCTTCCATG  14681
                     |||||||||||||||||||||||||||||||||||||||||||||||.||
KP025765       17911 agaagggcgggcaaggcgtcacaccaccgtcacttaccttgccttccctg  17960

ENSOARG000000  14682 CTAGTTCCTCGGGGAAACTGGACTTGTTGATAACCATCTTTCCGGTGTCC  14731
                     ||||||||||||||||||||||||||||||||||||||||||||||||||
KP025765       17961 ctagttcctcggggaaactggacttgttgataaccatctttccggtgtcc  18010

ENSOARG000000  14732 AGAGGGTCTGTTCTGGTCGCTGACCCCTCGTCCCCACTTGCTGCAGGACT  14781
                     ||||||||||||||||||||||||||||||||||||||||||||||||||
KP025765       18011 agagggtctgttctggtcgctgacccctcgtccccacttgctgcaggact  18060

ENSOARG000000  14782 TAGGCCTGCTCTGCCTCGGCCCTCGGTCTCAGCCCTCATCTCTGGTCTTG  14831
                     ||||||||||||||||||||||||||||||||||||||||||||||||||
KP025765       18061 taggcctgctctgcctcggccctcggtctcagccctcatctctggtcttg  18110

ENSOARG000000  14832 CTGCTGCCTGGCCTCCCTTCTCTGCCCTGACCCTTCCAGGATGTCAGCTG  14881
                     ||||||||||||||||||||||||||||||||||||||||||||||||||
KP025765       18111 ctgctgcctggcctcccttctctgccctgacccttccaggatgtcagctg  18160
```

```
ENSOARG000000  14882 GACACTGTTTCAGCCTTGTCAACTTTTCCTGGAAGACTTTTCCAGCCCCT  14931
                     ||||||||||||||||||||||||||||||||||||||||||||||||||
KP025765       18161 gacactgtttcagccttgtcaacttttcctggaagacttttccagcccct  18210

ENSOARG000000  14932 ATGTCCACCCTCCCCAGAGAGCAGAGGGGTGAGGAGTGATAAGAAGATTC  14981
                     ||||||||||||||||||||||||||||||||||||||||||||||||||
KP025765       18211 atgtccaccctccccagagagcagaggggtgaggagtgataagaagattc  18260

ENSOARG000000  14982 CTGAAGTCTGAGCTGGGCTGGGCCAGAGGGAGGGCAGGGGCTGAAAGGGG  15031
                     ||||||||||||||||||||||||||||||||||||||||||||||||||
KP025765       18261 ctgaagtctgagctgggctgggccagagggagggcaggggctgaaagggg  18310

ENSOARG000000  15032 GACTGTGGTCAAGATCCCTGAACAGCGCCTGGCTCCTTGCAGCCAGCGGG  15081
                     ||||||||||||||||||||||||||||||||||||||||||||||||||
KP025765       18311 gactgtggtcaagatccctgaacagcgcctggctccttgcagccagcggg  18360

ENSOARG000000  15082 ATCGTGTCCTTTGAGCAGCCCGAGTATTTGGTCAGCAGCGGGGAGCACGT  15131
                     ||||||||||||||||||||||||||||||||||||||||||||||||||
KP025765       18361 atcgtgtcctttgagcagcccgagtatttggtcagcagcggggagcacgt  18410

ENSOARG000000  15132 TGCCCGTATCCCTGTGGTCCGGCGCATCCTGGACAGCGGCAAGTCACAGG  15181
                     ||||||||||||||||||||||||||||||||||||||||||||||||||
KP025765       18411 tgcccgtatccctgtggtccggcgcatcctggacagcggcaagtcacagg  18460

ENSOARG000000  15182 TCTCCTACCGCACGCAGGACAACACCGCCAAGGGCAACCGGGTGAGTCCT  15231
                     ||||||||||||||||||||||||||||||||||||||||||||||||||
KP025765       18461 tctcctaccgcacgcaggacaacaccgccaagggcaaccgggtgagtcct  18510

ENSOARG000000  15232 TGCCACGAGGTCAGGCACATCCCAAGGGCCCAGTGGGGGCCGGACACAGC  15281
                     ||||||||||||||||||||||||||||||||||||||||||||||||||
KP025765       18511 tgccacgaggtcaggcacatcccaagggcccagtgggggccggacacagc  18560

ENSOARG000000  15282 GGCATCCTGGGTCTCTGGATGCTGGTGGTGCCACAGGCTGGCCTCCCAGG  15331
                     .|||||||||||||||||||||||||||||||||||||||||||||||||
KP025765       18561 agcatcctgggtctctggatgctggtggtgccacaggctggcctcccagg  18610

ENSOARG000000  15332 GACACGTTCCCTCTGCCACCCTGCAGCAGGGGCATCAGTTCAGTTCAGTC  15381
                     ||||||||||||||||||||||||||||||||||||||||||||||||||
KP025765       18611 gacacgttccctctgccaccctgcagcaggggcatcagttcagttcagtc  18660

ENSOARG000000  15382 GCTCAGTCATGTCCAACTCCTTGCGACCCCATGGACTGCAGCACCCCAGA  15431
                     ||||||||||||||||||||||||||||||||||||||||||||||||||
KP025765       18661 gctcagtcatgtccaactccttgcgaccccatggactgcagcaccccaga  18710

ENSOARG000000  15432 CCACCCTGTCCATCATCAACTCCTGGCGCTTACTCAAACTCATGTCCATT  15481
                     ||||||||||||||||||||||||||||||||||||||||||||||||||
KP025765       18711 ccaccctgtccatcatcaactcctggcgcttactcaaactcatgtccatt  18760

ENSOARG000000  15482 GAGTTGGTGATGCCATCCAGCCATCTCATCCTCTGTGTCCCCTTCTCCTC  15531
                     ||||||||||||||||||||||||||||||||||||||||||||||||||
KP025765       18761 gagttggtgatgccatccagccatctcatcctctgtgtccccttctcctc  18810

ENSOARG000000  15532 CCGCCTACAGTCTTTCCCAGCATCAGGGTCTTTTCAAATGAGTTGGGAAG  15581
                     ||||||||||||||||||||||||||||||||||||||||||||||||||
KP025765       18811 ccgcctacagtctttcccagcatcagggtcttttcaaatgagttgggaag  18860

ENSOARG000000  15582 ATCTCCTGGAGAAGGAAGTGGCAACCTACTCCTATTCTTGCCTAGAAAAC  15631
                     ||||||||||||||||||||||||||||||||||||||||||||||||||
KP025765       18861 atctcctggagaaggaagtggcaacctactcctattcttgcctagaaaac  18910

ENSOARG000000  15632 CCCATAGACAGAGGAGCCTAGTGGGCTACAGTCCATGGGGTGGCAAAAAG  15681
                     ||||||||||||||||||||||||||||||||||||||||||||||||||
KP025765       18911 cccatagacagaggagcctagtgggctacagtccatggggtggcaaaaag  18960

ENSOARG000000  15682 TTGGACAGGACTAAGCAATTTCACTTTCACCAAAAAGGCCAATGATGCCC  15731
```

```
                       ||||||||||||||||||||||||||||||||||||||||||||||||||
KP025765         18961 ttggacaggactaagcaatttcactttcaccaaaaaggccaatgatgccc  19010

ENSOARG000000    15732 TGAATAAGGCCTCCTCATGTCCAGACCATCCGAGTCCCCCTTGCAACATT  15781
                       |||||||||||||||||||||||||||||||||.||||||||||||||||
KP025765         19011 tgaataaggcctcctcatgtccagaccatccgtgtccccttgcaacatt  19060

ENSOARG000000    15782 CCAGGACCCACCCAGATACTTCAAAGAAATAGACTGCCCTCTCTGCCATG  15831
                       ||||||||||||||||||||||||||||||||||||||||||||||||||
KP025765         19061 ccaggacccacccagatacttcaaagaaatagactgccctctctgccatg  19110

ENSOARG000000    15832 GCCCAGAGACTCTGGCCTTCTTGTCCCCAGGCAGGCTCTCTGTCGGCCAG  15881
                       |||||||||||||||||||||||||||||||||||||.||||||||||||
KP025765         19111 gcccagagactctggccttcttgtccccaggcaggccctctgtcggccag  19160

ENSOARG000000    15882 GCCACAGGACCGATCCTTCAGGGCAGGGAGGGCCTCGTGGTGGCCTGGTG  15931
                       ||||||||||||||||||||||||||||||||||||||||||||||||||
KP025765         19161 gccacaggaccgatccttcagggcagggagggcctcgtggtggcctggtg  19210

ENSOARG000000    15932 TCAACTTTTTCGCAACTTAATCACTCTCAGACAACAGCCATGTCTGTGCT  15981
                       ||||||||||||||||||||||||||||||||||||||||||||||||||
KP025765         19211 tcaactttttcgcaacttaatcactctcagacaacagccatgtctgtgct  19260

ENSOARG000000    15982 TAGTTTGGCTTATACGTCTGTTTTTCTAAATTAATTACCAACGTTTTGTG  16031
                       ||||||||||||||||||||||||||||||||||||||||||||||||||
KP025765         19261 tagtttggcttatacgtctgtttttctaaattaattaccaacgttttgtg  19310

ENSOARG000000    16032 TATTTTGATTTTTTTGGCCATGCTGCGTGACTTGTGGGATCTTAGTCCCC  16081
                       ||||||||||||||||||||||||||||||||||||||||||||||||||
KP025765         19311 tattttgatttttttggccatgctgcgtgacttgtgggatcttagtcccc  19360

ENSOARG000000    16082 CAACCAGGGATCAAACCTGGACCCAGGGA-CTGGCAATGAAATTGAAGAG  16130
                       ||||||||||||||||||||||||||||| ||||||||||||||||||||
KP025765         19361 caaccagggatcaaacctggacccagggacctggcaatgaaattgaagag  19410

ENSOARG000000    16131 TCCTAACCACTGGACTTGGAGAAGGCAATGGCATCCCACTCCAGCACTCT  16180
                       ||||||||||||||||||||||||||||||||||||||||||||||||||
KP025765         19411 tcctaaccactggacttggagaaggcaatggcatcccactccagcactct  19460

ENSOARG000000    16181 TGCCTGGAAAATCCCATGGATGGAGGGGCCTGGTGGGCTGCAGTCCATGG  16230
                       ||||||||||||||||||||||||||||||||||||||||||||||||||
KP025765         19461 tgcctggaaaatcccatggatggaggggcctggtgggctgcagtccatgg  19510

ENSOARG000000    16231 GGTCTCAAACAGTCGGACACGGCTGAGCGACTTCACTTTCACTTTTCACT  16280
                       ||||||||||||||||||||||||||||||||||||||||||||||||||
KP025765         19511 ggtctcaaacagtcggacacggctgagcgacttcactttcacttttcact  19560

ENSOARG000000    16281 TTCATGCATTGGAGAAGGAACTGGCAACCCACTCCAGTGTTCTTGCCTGG  16330
                       ||||||||||||||||||||||||||||||||||||||||||||||||||
KP025765         19561 ttcatgcattggagaaggaactggcaacccactccagtgttcttgcctgg  19610

ENSOARG000000    16331 AGGATCCCAGGGACGGGGGAGCCTGGTGCGCTGCCGTCTATGGAGTCACA  16380
                       ||||||||||||||||||||||||||||||||||||||||||||||||||
KP025765         19611 aggatcccagggacgggggagcctggtgcgctgccgtctatggagtcaca  19660

ENSOARG000000    16381 CAGAGTCGGACACGACTGAAGCGACTTAGCAACCACTGGACTGCCAGGGA  16430
                       ||||||||||||||||||||||||||||||||||||||||||||||||||
KP025765         19661 cagagtcggacacgactgaagcgacttagcaaccactggactgccaggga  19710

ENSOARG000000    16431 ATTCCCTATTACCATTACCAATTTTTTTAAATTAATTTTTTTATTGGCAT  16480
                       ||||||||||||||||||||||||||||.|||||||||||||||||||||
KP025765         19711 attccctattaccattaccaattttttttaattaatttttttattggcat  19760

ENSOARG000000    16481 ACAGTTGATTTACCATATTGTGTTTCTGCTGTACAGCTAAGTGAATCAGT  16530
                       ||||||||||||||||||||||||||||||||||||||||||||||||||
```

```
KP025765         19761 acagttgatttaccatattgtgtttctgctgtacagctaagtgaatcagt  19810

ENSOARG000000    16531 TAAACATAGACATATATCGACTCTTTTTCAGATTCTGTTCCCATATAGAT  16580
                       ||||||||||||||||||||||||||||||||||||||||||||||||||
KP025765         19811 taaacatagacatatatcgactctttttcagattctgttcccatatagat  19860

ENSOARG000000    16581 CATTACAGAGTACTGAGTAGAGTTTCCTGTGCTATACCATAGGTTCTTAT  16630
                       ||||||||||||||||||||||||||||||||||||||||||||||||||
KP025765         19861 cattacagagtactgagtagagtttcctgtgctataccataggttcttat  19910

ENSOARG000000    16631 TAGTTATCTATTTTATAACTATTACCAACATTTTAAAACTAAAAATATTC  16680
                       ||||||||||||||||||||||||||||||||||||||||||||||||||
KP025765         19911 tagttatctattttataactattaccaacattttaaaactaaaaatattc  19960

ENSOARG000000    16681 ATATCAAAACTTCACTGGACTTTCCTCTTAGACCAGTAGTTAAGATGCTG  16730
                       |||||||||||||||||||||||||||||.||||||||||||||||||||
KP025765         19961 atatcaaaacttcactggactttcctcttggaccagtagttaagatgctg  20010

ENSOARG000000    16731 CGCTTTCACTGCAGGGGGCATGGGTTCGATCCTGGTTTGGGGAAGTTCCA  16780
                       ||||||||||||||||||||||||||||||||||||||||||||||||||
KP025765         20011 cgctttcactgcagggggcatgggttcgatcctggtttggggaagttcca  20060

ENSOARG000000    16781 TATGCCACAGAGTGTGCCCCAAAATTAAAAACGAAACAAAACTTCACTCA  16830
                       ||||||||||||||||||||||||||||||||||||||||||||||||||
KP025765         20061 tatgccacagagtgtgccccaaaattaaaaacgaaacaaaacttcactca  20110

ENSOARG000000    16831 AAAGTAATAAAGGTTAGTCGCACTGGCCTGCAGGCCCACTTGGCAATCAT  16880
                       ||||||||||||||||||||||||||||||||||||||||||||||||||
KP025765         20111 aaagtaataaaggttagtcgcactggcctgcaggcccacttggcaatcat  20160

ENSOARG000000    16881 GAGAGCAAGTGTGCGCACACAGCCTATTCTTTATTTCTTTTTATAGGTAG  16930
                       ||||||||||||||||||||||||||||||||||||||||||||||||||
KP025765         20161 gagagcaagtgtgcgcacacagcctattctttatttctttttataggtag  20210

ENSOARG000000    16931 GACTAGTTCTCATTACTGCTCTAATGCTCCCAGTAAAAATTCCAAAAAGG  16980
                       ||||||||||||||||||||||||||||||||||||||||||||||||||
KP025765         20211 gactagttctcattactgctctaatgctcccagtaaaaattccaaaaagg  20260

ENSOARG000000    16981 AATCTGTGCAACCATAACTTCACTGAAAGTGAAAAAGTGAAAGTGTTAAA  17030
                       ||||||||||||||||||||||||||||||||||||||||||||||||||
KP025765         20261 aatctgtgcaaccataacttcactgaaagtgaaaaagtgaaagtgttaaa  20310

ENSOARG000000    17031 TCGCTAAGTCATGTCTGACTCTTGGTGGCCCATGGACTGCAGCCTACCAG  17080
                       ||||||||||||||||||||||||||||||||||||||||||||||||||
KP025765         20311 tcgctaagtcatgtctgactcttggtggcccatggactgcagcctaccag  20360

ENSOARG000000    17081 TCTCCTCTGTCCATGGGATTTTTCCAGGCAAGAATACTGGAGTGGATTGC  17130
                       ||||||||||||||||||||||||||||||||||||||||||||||||||
KP025765         20361 tctcctctgtccatgggatttttccaggcaagaatactggagtggattgc  20410

ENSOARG000000    17131 CTTTCCTTTCTCCAGGGGATCTTCCCCACCCAGGGATCAAACCTGAGTCT  17180
                       ||||||||||||||||||||||||||||||||||||||||||||||||||
KP025765         20411 ctttcctttctccaggggatcttccccacccagggatcaaacctgagtct  20460

ENSOARG000000    17181 CCTGCATTGCGGGCAGATTCTTTACCATCTGAGCCACTAGGGAAGCCCTT  17230
                       ||||||||||||||||||||||||||||||||||||||||||||||||||
KP025765         20461 cctgcattgcgggcagattctttaccatctgagccactagggaagccctt  20510

ENSOARG000000    17231 TATAAAGATCCTTTGGTAGTGAACTATGTTTTTTAACATTTTTAAATATT  17280
                       ||||||||||||||||||||||||||||||||||||||||||.|||||||
KP025765         20511 tataaagatcctttggtagtgaactatgttttttaacattttaaaatatt  20560

ENSOARG000000    17281 TCTCAACTTTTTATTTTGAAAAACTTCAAACCTACAGCAGAATGGAAAGA  17330
                       ||||||||||||||||||||||||||||||||||||||||||||||||||
KP025765         20561 tctcaactttttattttgaaaaacttcaaacctacagcagaatggaaaga  20610
```

```
ENSOARG000000  17331 CTAATAAAATTAGCACCCATTTACTCTTGACATAGATTCACCCATTGTTG  17380
                     ||||||||||||||||||||||||||||||||||||||||||||||||||
KP025765       20611 ctaataaaattagcacccatttactcttgacatagattcacccattgttg  20660

ENSOARG000000  17381 ATAGTTGCTTTCTCTCTTTCTATACAGATGTATATATGTATGTGTATACA  17430
                     ||||||||||||||||||||||||||||||||||||||||||||||||||
KP025765       20661 atagttgctttctctctttctatacagatgtatatatgtatgtgtataca  20710

ENSOARG000000  17431 GATATATGCCTGGGGGTATAGCTCAGTGGTAGAGCGCATGCTTAGCATGC  17480
                     ||||||||||||||||||||||||||||||||||||||||||||||||||
KP025765       20711 gatatatgcctgggggtatagctcagtggtagagcgcatgcttagcatgc  20760

ENSOARG000000  17481 ATGAGGCCCTGGGTTCAATCCCCAGTACCTCCATCCTTACATTTTTCGGC  17530
                     ||||||||||||||||||||||||||||||||||||||||||||||||||
KP025765       20761 atgaggccctgggttcaatccccagtacctccatccttacatttttcggc  20810

ENSOARG000000  17531 TTCCCTGGTGGCTCGGACTGCTAGCAAAGAATCTGAATGTTAGTTTACAG  17580
                     ||||||||||||||||||||||||||||||||||||||||||||||||||
KP025765       20811 ttccctggtggctcggactgctagcaaagaatctgaatgttagtttacag  20860

ENSOARG000000  17581 TGCCACCTAGTGGAATCATGTGAAAAGGCTTCCCCCTACACACACTGCAA  17630
                     ||||||||||||||||||||||||||||||||||||||||||||||||||
KP025765       20861 tgccacctagtggaatcatgtgaaaaggcttcccccTacacacactgcaa  20910

ENSOARG000000  17631 TCCATCTGCCCCATATCAGCTCAGTTCAGTACAGTCGTGTCCGACTCTTT  17680
                     ||||||||||||||||||||||||||||||||||||||||||.|||||||
KP025765       20911 tccatctgccccatatcagctcagttcagtacagtcgtgtccaactcttt  20960

ENSOARG000000  17681 ACGACCCCATGAATCACAGCACGCCAGGCCTCCCTGTCCATCACCAACTC  17730
                     ||||||||||||||||||||||||||||||||||||||||||||||||||
KP025765       20961 acgaccccatgaatcacagcacgccaggcctccctgtccatcaccaactc  21010

ENSOARG000000  17731 CAGGAGTCCACTCAAAGTCACGTCCATCGAGTCAGTGATGCCATCCAGCC  17780
                     |.||||||||||||||||||||||||||||||||||||||||||||||||
KP025765       21011 ccggagtccactcaaagtcacgtccatcgagtcagtgatgccatccagcc  21060

ENSOARG000000  17781 ATCTCATCCTCTGTTGTCCCCTTCTCCTCCTGCCCCCAATCCCTCCCAGC  17830
                     ||||||||||||||||||||||||||||||||||||||||||||||||||
KP025765       21061 atctcatcctctgttgtccccttctcctcctgcccccaatccctcccagc  21110

ENSOARG000000  17831 ATCAGAGTATTTTCCAATGAGTCAACTCTTCACATGAGATGGCCAAAGTA  17880
                     ||||||||||||||||||||||||||||||||||||||||||||||||||
KP025765       21111 atcagagtattttccaatgagtcaactcttcacatgagatggccaaagta  21160

ENSOARG000000  17881 CTGGAGTTTCAACTTTAGCATCATTCCTTCCAAAGAAAACCCAGGGCTGA  17930
                     ||||||||||||||||||||||||||||||||||||||||||||||||||
KP025765       21161 ctggagtttcaactttagcatcattccttccaaagaaaacccagggctga  21210

ENSOARG000000  17931 TCTCCTTCAGAATGGACTGGTTGGATCTCCTTGCAGTCCAAGGGACTCTC  17980
                     ||||||||||||||||||||||||||||||||||||||||||||||||||
KP025765       21211 tctccttcagaatggactggttggatctccttgcagtccaagggactctc  21260

ENSOARG000000  17981 AAGAGTCTTCTCCAACATCACAGTTCAAAAGCATCAATTCTTTGGCGCTC  18030
                     ||||||||||||||||||||||||||||||||||||||||||||||||||
KP025765       21261 aagagtcttctccaacatcacagttcaaaagcatcaattctttggcgctc  21310

ENSOARG000000  18031 AGCCTTCTTCACAGTCCAACTCTCACATCCATACATGACCACTGGGAAAA  18080
                     ||||||||||||||||||||||||||||||||||||||||||||||||||
KP025765       21311 agccttcttcacagtccaactctcacatccatacatgaccactgggaaaa  21360

ENSOARG000000  18081 CCATAGCTTTGACTAGATGGACCTTTGTTGGCAAAGTAATGTCTCTGCTT  18130
                     ||||||||||||||||||||||||||||||||||||||||||||||||||
KP025765       21361 ccatagctttgactagatggacctttgttggcaaagtaatgtctctgctt  21410
```

```
ENSOARG000000     18131 TTCAATATGCTATCTAGGCTGGTCATAACTTTCCTTTCAAGGAGTGAGCG  18180
                        ||||||||||||||||||||||||||||||||||||||||||||||||||
KP025765          21411 ttcaatatgctatctaggctggtcataactttcctttcaaggagtgagcg  21460

ENSOARG000000     18181 TCTTTTAATTTCATGGCTGTAGTCACCATCTGCAGTGATTTTGGAGCCCC  18230
                        ||||||||||||||||||||||||||||||||||||||||||||||||||
KP025765          21461 tcttttaatttcatggctgtagtcaccatctgcagtgattttggagcccc  21510

ENSOARG000000     18231 AAAAAATAAAGTCTGACACTGTTTCCACTGTTTCCCCATCTATTTCCCAT  18280
                        ||||||||||||||||||||||||||||||||||||||||||||||||||
KP025765          21511 aaaaaataaagtctgacactgtttccactgtttccccatctatttcccat  21560

ENSOARG000000     18281 GAAGTGATGGGACCGGATGCCATGATCTTTGTTTTCTGACTTAATCTCAA  18330
                        ||||||||||||||||||||||||||||||||||||||||||||||||||
KP025765          21561 gaagtgatgggaccggatgccatgatctttgttttctgacttaatctcaa  21610

ENSOARG000000     18331 GATGTTCAGGAAAAAGCTCTAGGGCATTGAAAAATAAAAACTGGAGGTAA  18380
                        ||||||||||||||||||||||||||||||||||||||||||||||||||
KP025765          21611 gatgttcaggaaaaagctctagggcattgaaaaataaaaactggaggtaa  21660

ENSOARG000000     18381 TAGAAGCAACCCACCCAGGAAGCAGGGCCGGGGGGATGGTGGGATTCAGA  18430
                        ||||||||||||||||||||||||||||||||||||||||||||||||||
KP025765          21661 tagaagcaacccacccaggaagcagggccggggggatggtgggattcaga  21710

ENSOARG000000     18431 CTTCAGAGCCCCAGGGTGGGGAAGAGTTCCTCCCTCCGAGGTGAGGCCTG  18480
                        ||||||||||||||||||||||||||||||||||||||||||||||||||
KP025765          21711 cttcagagccccagggtggggaagagttcctccctccgaggtgaggcctg  21760

ENSOARG000000     18481 CAAATCTCAGCTCCCCTGGCTGAGTGCCTGCCTGTGTGGGACCCTCCTGT  18530
                        ||||||||||||||||||||||||||||||||||||||||||||||||||
KP025765          21761 caaatctcagctcccctggctgagtgcctgcctgtgtgggaccctcctgt  21810

ENSOARG000000     18531 ATCTGCATCCTTGTCTCTCTGTGCCTTGGTTCAAATCCCAGTTCCACCAC  18580
                        ||||||||||||||||||||||||||||||||||||||||||||||||||
KP025765          21811 atctgcatccttgtctctctgtgccttggttcaaatcccagttccaccac  21860

ENSOARG000000     18581 AACTACCTGTGTGAACTTGGGCATCTTTCTTTACCTCTGTGTGCCTCAGT  18630
                        ||||||||||||||||||||||||||||||||||||||||||||||||||
KP025765          21861 aactacctgtgtgaacttgggcatctttctttacctctgtgtgcctcagt  21910

ENSOARG000000     18631 TACCTGATCTGTAAAATGGGGACAGTAATATCTCTACCTCACTCTGCCCT  18680
                        ||||||||||||||||||||||||||||||||||||||||||||||||||
KP025765          21911 tacctgatctgtaaaatggggacagtaatatctctacctcactctgccct  21960

ENSOARG000000     18681 GAGGAAGAAACGAGCTAATATCTGTAAAGTACTTGATGCACAAGAGGTGC  18730
                        ||||||||||||||||||||||||||||||||||||||||||||||||||
KP025765          21961 gaggaagaaacgagctaatatctgtaaagtacttgatgcacaagaggtgc  22010

ENSOARG000000     18731 TGTATAAACAGGAGCCGTTCCGTGTCCATGTGGGACAGTCTGTGCGTGAG  18780
                        ||||||||||||||||||||||||||||||||||||||||||||||||||
KP025765          22011 tgtataaacaggagccgttccgtgtccatgtgggacagtctgtgcgtgag  22060

ENSOARG000000     18781 GGTCTCTGCATTGGCTGTGGGGTGGGGGTGGAGCGTGTAGCCTTGACTCG  18830
                        ||||||||||||||||||||||||||||||||||||||||||||||||||
KP025765          22061 ggtctctgcattggctgtggggtgggggtggagcgtgtagccttgactcg  22110

ENSOARG000000     18831 TCTCCCAGGCCTGCTCCAACTCTGACCCCTTCCCATTCCTAGGACTACAT  18880
                        ||||||||||||||||||||||||||||||||||||||||||||||||||
KP025765          22111 tctcccaggcctgctccaactctgaccccttcccattcctaggactacat  22160

ENSOARG000000     18881 CCCCATGGAGGGTGAGCTGCTGTTCCAACCTGGAGAGACCTGGAAGGAGC  18930
                        ||||||||||||||||||||||||||||||||||||||||||||||||||
KP025765          22161 ccccatggagggtgagctgctgttccaacctggagagacctggaaggagc  22210

ENSOARG000000     18931 TGCAGGTGAAGCTGCTGGAGCTGCAGGAGATGGACTCCCTCCTGCGGGGC  18980
```

```
                          ||||||||||||||||||||||||||||||||||||||||||||||||||
KP025765           22211 tgcaggtgaagctgctggagctgcaggagatggactccctcctgcggggc  22260

ENSOARG000000      18981 CCTCAGACCCGCCGCTTCTACATCCAACTCAGCAACCCCAAGTTTGGGGC  19030
                          ||||||||||||||||||||||||||||||||||||||||||||||||||
KP025765           22261 cctcagacccgccgcttctacatccaactcagcaaccccaagtttggggc  22310

ENSOARG000000      19031 CCGCCTGGGCCAGCCCCAGTCTGCCACTGTCATCATTGGGGACCGAGGTA  19080
                          ||||||||||||||||||||||||||||||||||||||||||||||||||
KP025765           22311 ccgcctgggccagccccagtctgccactgtcatcattggggaccgaggta  22360

ENSOARG000000      19081 GCCAGAGCCTGGGGTCAGGTTAAGCAGGTGGGAAGGGAGGGCTGGGGCTT  19130
                          ||||||||||||||||||||||||||||||||||||||||||||||||||
KP025765           22361 gccagagcctggggtcaggttaagcaggtgggaagggagggctggggctt  22410

ENSOARG000000      19131 CTTGAAAGTCTCAGCCGTAAAAATACAGAATGCTGAGTTAAATTTGAATC  19180
                          ||||||||||||||||||||||||||||||||||||||||||||||||||
KP025765           22411 cttgaaagtctcagccgtaaaaatacagaatgctgagttaaatttgaatc  22460

ENSOARG000000      19181 CCAGATAAATAACAACTCAACTTGTAGTATAAATATATCCCATATGCTAT  19230
                          ||||||||||||||||||||||||||||||||||||||||||||||||||
KP025765           22461 ccagataaataacaactcaacttgtagtataaatatatcccatatgctat  22510

ENSOARG000000      19231 CTGGGATATACTTATTCTTAAAAAGTATCTTTGTGTGAAATTCAAGTTTA  19280
                          ||||||||||||||||||||||||||||||||||||||||||||||||||
KP025765           22511 ctgggatatacttattcttaaaaagtatctttgtgtgaaattcaagttta  22560

ENSOARG000000      19281 ACTAGGCATCTTGTATTTATCTGACAACCTGCTCTCAATCAGAGAGACCA  19330
                          ||||||||||||||||||||||||||||||||||||||||||||||||||
KP025765           22561 actaggcatcttgtatttatctgacaacctgctctcaatcagagagacca  22610

ENSOARG000000      19331 GGGTCATGTGGCCACGGGCCTGCTGCTGGCTGGGAATCCCACCATTAAGC  19380
                          ||||||||||||||||||||||||||||||||||||||||||||||||||
KP025765           22611 gggtcatgtggccacgggcctgctgctggctgggaatcccaccattaagc  22660

ENSOARG000000      19381 CTTAGCCCTTTGACTTCAGCCTTAGTTCAAGCTAAAATGCAAGTGTTTCA  19430
                          ||||||||||||||||||||||||||||||||||||||||||||||||||
KP025765           22661 cttagccctttgacttcagccttagttcaagctaaaatgcaagtgtttca  22710

ENSOARG000000      19431 GAAGGGGTGAAACACTTCTTATGTGCCCTTAGCTTCCAGTATAGTGGGGC  19480
                          ||||||||||||||||||||||||||||||||||||||||||||||||||
KP025765           22711 gaaggggtgaaacacttcttatgtgcccttagcttccagtatagtggggc  22760

ENSOARG000000      19481 CTGTGAGCCCACCCTGGAGGAGAGGTTTGCCAAGAGGATGGGGGCGGAGG  19530
                          ||||||||||||||||||||||||||||||||||||||||||||||||||
KP025765           22761 ctgtgagcccaccctggaggagaggtttgccaagaggatgggggcggagg  22810

ENSOARG000000      19531 AGGAGTCTTGGGGTAGGGGAGAGAGGAAGGGCATTGGAGGAGAAAATGTT  19580
                          ||||||||||||||||||||||||||||||||||||||||||||||||||
KP025765           22811 aggagtcttggggtaggggagagaggaagggcattggaggagaaaatgtt  22860

ENSOARG000000      19581 CCCTGTGGGTCACGCCTTGGCTGCCAAAGGGACAAAGAGAGCAAGCCCTC  19630
                          ||||||||||||||||||||||||||||||||||||||||||||||||||
KP025765           22861 ccctgtgggtcacgccttggctgccaaagggacaaagagagcaagccctc  22910

ENSOARG000000      19631 ATCCCTCTGGAGACCCTCCTGGGTCCCCCTGAAGCAGGGGGAAGGTTCAG  19680
                          ||||||||||||||||||||||||||||||||||||||||||||||||||
KP025765           22911 atccctctggagaccctcctgggtccccctgaagcagggggaaggttcag  22960

ENSOARG000000      19681 TTCAGTTCAGTTCAGTTCACTCGCTCAGTCGTGTCAGACTCTTTGTGATC  19730
                          ||||||||||||||||||||||||||||||||||||||||||||||||||
KP025765           22961 ttcagttcagttcagttcactcgctcagtcgtgtcagactctttgtgatc  23010

ENSOARG000000      19731 CCATGGACCGCGGCCTCCCTGTTCATCACCAACTCTCGGAGTTTACTCAA  19780
                          ||||||||||||||||||||||||||||||||||||||||||||||||||
```

```
KP025765         23011 ccatggaccgcggcctccctgttcatcaccaactctcggagtttactcaa  23060

ENSOARG000000    19781 ACTCATGTCTATTGAGTTGGTGATGCCATCTAACCATCTCATCCTCTGTT  19830
                       ||||||||||||||||||||||||||||||||||||||||||||||||||
KP025765         23061 actcatgtctattgagttggtgatgccatctaaccatctcatcctctgtt  23110

ENSOARG000000    19831 GTCCCCTTCTCCTCCTGCCTTCAATATTTCCCAGCGTCAGGGTCTTTTCA  19880
                       ||||||||||||||||||||||||||||||||||||||||||||||||||
KP025765         23111 gtccccttctcctcctgccttcaatatttcccagcgtcagggtcttttca  23160

ENSOARG000000    19881 AATGAGTCAGTTCTTCGCATCAGGTCGCCAAAATATTGGAGTTTCTGCTT  19930
                       ||||||||||||||||||||||||||||||||||||||||||||||||||
KP025765         23161 aatgagtcagttcttcgcatcaggtcgccaaaatattggagtttctgctt  23210

ENSOARG000000    19931 CAACATCAGTCCTTCCAATGAACATTCAGGACTGATCTTTAGAATGGACT  19980
                       ||||||||||||||||||||||||||||||||||||||||||||||||||
KP025765         23211 caacatcagtccttccaatgaacattcaggactgatctttagaatggact  23260

ENSOARG000000    19981 GGTTGGATCTCCTTGCAGTCCAAGGGACTCTTCAAGAGTCTTCTCCAACA  20030
                       ||||||||||||||||||||||||||||||||||||||||||||||||||
KP025765         23261 ggttggatctccttgcagtccaagggactcttcaagagtcttctccaaca  23310

ENSOARG000000    20031 CCACAGTTCACAAGCGTCAATACTTTGGCCCTCAGCTTTCTTTATAGTCC  20080
                       ||||||||||||||||||||||||||||||||||||||||||||||||||
KP025765         23311 ccacagttcacaagcgtcaatactttggccctcagctttctttatagtcc  23360

ENSOARG000000    20081 AACTCTCATATCCATACATGTGATGTGATGTGAAGGGGAAGGAGTCTTGG  20130
                       ||||||||||||||||||||||||||||||||||||||||||||||||||
KP025765         23361 aactctcatatccatacatgtgatgtgatgtgaaggggaaggagtcttgg  23410

ENSOARG000000    20131 TGTTTAACTGTGCGTCTGGTGCCCCCTGGTGGTGCAGATGGGTTCCTGCC  20180
                       ||||||||||||||||||||||||||||||||||||||||||||||||||
KP025765         23411 tgtttaactgtgcgtctggtgccccctggtggtgcagatgggttcctgcc  23460

ENSOARG000000    20181 CCCTCAAAGCCAGAGAGGAGTCTCTGCTGACTGCCTTGCCCTGGCTTGAC  20230
                       ||||||||||||||||||||||||||||||||||||||||||||||||||
KP025765         23461 ccctcaaagccagagaggagtctctgctgactgccttgccctggcttgac  23510

ENSOARG000000    20231 CCTCTGCAGATGAGCTGGACCGGAACTTAATGAACCAGACTGTGTCATCA  20280
                       ||||||||||||||||||||||||||||||||||||||||||||||||||
KP025765         23511 cctctgcagatgagctggaccggaacttaatgaaccagactgtgtcatca  23560

ENSOARG000000    20281 CCCCCACCACCCCGGGGTGACCTGGGTGCCCCACAGAATCCCAATGCCAA  20330
                       ||||||||||||||||||||||||||||||||||||||||||||||||||
KP025765         23561 cccccaccaccccggggtgacctgggtgccccacagaatcccaatgccaa  23610

ENSOARG000000    20331 GGCTGCCGGGTCCCGGAAGATCCACTTCAACTGGTTGCCGCCTCCTGGCA  20380
                       ||||||||||||||||||||||||||||||||||||||||||||||||||
KP025765         23611 ggctgccgggtcccggaagatccacttcaactggttgccgcctcctggca  23660

ENSOARG000000    20381 AGCCAACAGGGTACCGGGTAAGGTGGGGGGCGTTGAGGGAGGTGGATGGG  20430
                       ||||||||||||||||||||||||||||||||||||||||||||||||||
KP025765         23661 agccaacagggtaccgggtaaggtggggggcgttgagggaggtggatggg  23710

ENSOARG000000    20431 AGGCCGGGCATACACTGTTGAGGTGAGCCCGCTGA---------------  20465
                       |||||||||||||||||||||||||||||||||||
KP025765         23711 aggccgggcatacactgttgaggtgagcccgctgaggccagagagggccc  23760

ENSOARG000000    20466 --------------------------------------------------  20465

KP025765         23761 cagggagaggagagggcgtgcttggcctcagtgggtggggaacccccatc  23810

ENSOARG000000    20466 -GCCGCTGAGGGAGGAAGAAGTCCTTGGAATGTGGCCCCCAGAACCCAGA  20514
                        |||||||||||||||||||||||||||||||||||||||||||||||||
KP025765         23811 ggccgctgagggaggaagaagtccttggaatgtggcccccagaacccaga  23860
```

```
ENSOARG000000  20515 AACACCTGTGCTCTGGGCCAGCCCTGCTGCGTGCGCCTGGCAGGGCACTC  20564
                     ||||||||||||||||||||||||||||||||||||||||||||||||||
KP025765       23861 aacacctgtgctctgggccagccctgctgcgtgcgcctggcagggcactc  23910

ENSOARG000000  20565 AATCCCTCCACGTCTGTGTGCCCACGGGACTGTAAGGGCAGGGCTCAGCG  20614
                     ||||||||||||||||||||||||||||||||||||||||||||||||||
KP025765       23911 aatccctccacgtctgtgtgcccacgggactgtaagggcagggctcagcg  23960

ENSOARG000000  20615 GAGGGACGGTGGGCTCTGCCAGCTGCTCCCCACTGATCAGCCTTGCCCTG  20664
                     ||||||||||||||||||||||||||||||||||||||||||||||||||
KP025765       23961 gagggacggtgggctctgccagctgctccccactgatcagccttgccctg  24010

ENSOARG000000  20665 GGCACAGGTGAAGTACTGGATCCAGGGCGACTCCGAGTCTGAAGCCCACC  20714
                     ||||||||||||||||||||||||||||||||||||||||||||||||||
KP025765       24011 ggcacaggtgaagtactggatccagggcgactccgagtctgaagcccacc  24060

ENSOARG000000  20715 TGCTCGACAGCAAGGTCCCCTCAGTGGAGCTCACCAACCTGTACCCATAT  20764
                     ||||||||||||||||||||||||||||||||||||||||||||||||||
KP025765       24061 tgctcgacagcaaggtcccctcagtggagctcaccaacctgtacccatat  24110

ENSOARG000000  20765 TGCGACTACGAGATGAAGGTGTGCGCCTACGGGGCGCAGGGCGAGGGCCC  20814
                     ||||||||||||||||||||||||||||||||||||||||||||||||||
KP025765       24111 tgcgactacgagatgaaggtgtgcgcctacggggcgcagggcgagggccc  24160

ENSOARG000000  20815 CTACAGCTCCCCGGTGTCCTGTCGCACCCACCAGGAAGGTGAGGCCTCGC  20864
                     ||||||||||||||||||||||||||||||||||||||||||||||||||
KP025765       24161 ctacagctccccggtgtcctgtcgcacccaccaggaaggtgaggcctcgc  24210

ENSOARG000000  20865 CACGTCCATCCCTGGCCACCCTGATGCCAGCCTGCCCTGCCCCGGCCCCG  20914
                     ||||||||||||||||||||||||||||||||||||||||||||||||||
KP025765       24211 cacgtccatccctggccaccctgatgccagcctgccctgccccggccccg  24260

ENSOARG000000  20915 CCCAGAACTCTGCCCTCTTGTCCGCTGTTGACAGCACTCTTCCTGCACCC  20964
                     ||||||||||||||||||||||||||||||||||||||||||||||||||
KP025765       24261 cccagaactctgccctcttgtccgctgttgacagcactcttcctgcaccc  24310

ENSOARG000000  20965 CCTCTGCCCCACCCAGTGCCCAGCGAGCCCGGGCGTCTGGCCTTCAACGT  21014
                     ||||||||||||||||||||||||||||||||||||||||||||||||||
KP025765       24311 cctctgccccacccagtgcccagcgagcccgggcgtctggccttcaacgt  24360

ENSOARG000000  21015 CGTCTCCTCCACTGTGACCCAGCTGAGCTGGGCTGAGCCAGCCGAGACCA  21064
                     ||||||||||||||||||||||||||||||||||||||||||||||||||
KP025765       24361 cgtctcctccactgtgacccagctgagctgggctgagccagccgagacca  24410

ENSOARG000000  21065 ACGGCGAGATCACAGCCTATGAGGTCTGCTACGGCCTGGTCAATGAGGAC  21114
                     ||||||||||||||||||||||||||||||||||||||||||||||||||
KP025765       24411 acggcgagatcacagcctatgaggtctgctacggcctggtcaatgaggac  24460

ENSOARG000000  21115 AACCGTAAGGGCCCGCCCTCGCCTTCTGTCTCCAGGGAGGGGAGGGGGAG  21164
                     ||||||||||||||||||||||||||||||||||||||||||||||||||
KP025765       24461 aaccgtaagggcccgccctcgccttctgtctccagggaggggagggggag  24510

ENSOARG000000  21165 TGTCTGGTTGCCTGGGGGCATCATTAGGTTTGTGGTCAGGTTTGGGCTGG  21214
                     ||||||||||||||||||||||||||||||||||||||||||||||||||
KP025765       24511 tgtctggttgcctgggggcatcattaggtttgtggtcaggtttgggctgg  24560

ENSOARG000000  21215 GTTAGTTGCATTCCAAAGCAGAGCCCAGAAGCTCCCAGGGCAGGTGAGGA  21264
                     ||||||||||||||||||||||||||||||||||||||||||||||||||
KP025765       24561 gttagttgcattccaaagcagagcccagaagctcccagggcaggtgagga  24610

ENSOARG000000  21265 TGGAAGGCCTGAGGTTATGTGTGTGCAGTGGGGTCACAGGTCCAGAGTGC  21314
                     ||||||||||||||||||||||||||||||||||||||||||||||||||
KP025765       24611 tggaaggcctgaggttatgtgtgtgcagtggggtcacaggtccagagtgc  24660
```

```
ENSOARG000000     21315 CTGCCCACGCAGGTTAACGAGAGTGGCTCAATTCTACATGGGTGTCTTCA  21364
                        ||||||||||||||||||||||||||||||||||||||||||||||||||
KP025765          24661 ctgcccacgcaggttaacgagagtggctcaattctacatgggtgtcttca  24710

ENSOARG000000     21365 GGTCAGGGTAGAGAGATTTAGCAAATAAAAAGCAGAGGACGCCCTGAACT  21414
                        ||||||||||||||||||||||||||||||||||||||||||||||||||
KP025765          24711 ggtcagggtagagagatttagcaaataaaaagcagaggacgccctgaact  24760

ENSOARG000000     21415 TCAAACAGTTTTTCTAGTACATTTCATGCAACGTGTTATCTGGCAGCCCT  21464
                        ||||||||||||||||||||||||||||||||||||||||||||||||||
KP025765          24761 tcaaacagtttttctagtacatttcatgcaacgtgttatctggcagccct  24810

ENSOARG000000     21465 AGGCCAGAGCACTAGGCAGAACTCTATTTTTCTCCTGTCCTGGAAGGGAG  21514
                        ||||||||||||||||||||||||||||||||||||||||||||||||||
KP025765          24811 aggccagagcactaggcagaactctatttttctcctgtcctggaagggag  24860

ENSOARG000000     21515 AGATGACCCCTGCACTTGTCAGGACGGAAAACTTTTCAGGGGAGCTCAGT  21564
                        ||||||||||||||||||||||||||||||||||||||||||||||||||
KP025765          24861 agatgacccctgcacttgtcaggacggaaaacttttcaggggagctcagt  24910

ENSOARG000000     21565 CCCCGACTCTTGCCTGGTACAGGGCCCTCCTCTCTGCATCCTCACATGTT  21614
                        ||||||||||||||||||||||||||||||||||||||||||||||||||
KP025765          24911 ccccgactcttgcctggtacagggccctcctctctgcatcctcacatgtt  24960

ENSOARG000000     21615 CAGTCCATCCCAGGGATGGAGAGCTTCCCACTCCCAGATACAACCTGTGG  21664
                        ||||||||||||||||||||||||||||||||||||||||||||||||||
KP025765          24961 cagtccatcccagggatggagagcttcccactcccagatacaacctgtgg  25010

ENSOARG000000     21665 ACCCTAAAGTGGGTCAGACTTAGCACTCCCAGGTGGCCCAACAGGGCAGA  21714
                        ||||||||||||||||||||||||||||||||||||||||||||||||||
KP025765          25011 accctaaagtgggtcagacttagcactcccaggtggcccaacagggcaga  25060

ENSOARG000000     21715 GGGCAGCAGGATTGTTCTGGGCCTCAGGCTCCTTGCTCTGTTCACCTGGC  21764
                        ||||||||||||||||||||||||||||||||||||||||||||||||||
KP025765          25061 gggcagcaggattgttctgggcctcaggctccttgctctgttcacctggc  25110

ENSOARG000000     21765 TGACCAAGCACAACCTTTTCAGCCGCTGCGTCACACCTTGGCTCCTGCTG  21814
                        ||||||||||||||||||||||||||||||||||||||||||||||||||
KP025765          25111 tgaccaagcacaaccttttcagccgctgcgtcacaccttggctcctgctg  25160

ENSOARG000000     21815 GCGACCCCTGATCCACGCCTGGACCACTGTCAGGAAGTCTTTTGTGGCCC  21864
                        ||||||||||||||||||||||||||||||||||||||||||||||||||
KP025765          25161 gcgacccctgatccacgcctggaccactgtcaggaagtcttttgtggccc  25210

ENSOARG000000     21865 GGTCTTTAATCTTAACCAGGCGTGACTGATGATTCCGGCGCACAGTCTTA  21914
                        ||||||||||||||||||||||||||||||||||||||||||||||||||
KP025765          25211 ggtctttaatcttaaccaggcgtgactgatgattccggcgcacagtctta  25260

ENSOARG000000     21915 GCTCTTGCCTCACAAACGTGTGAGGAAGCCCTCCTGACCCAAGGGCATCA  21964
                        ||||||||||||||||||||||||||||||||||||||||||||||||||
KP025765          25261 gctcttgcctcacaaacgtgtgaggaagccctcctgacccaagggcatca  25310

ENSOARG000000     21965 GCCCTTTGGGGTGGTCCCCTTCCCTTCTCCCCCTGGCAGACAGATGCGAC  22014
                        ||||||||||||||||||||||||||||||||||||||||||||||||||
KP025765          25311 gccctttggggtggtccccttcccttctccccctggcagacagatgcgac  25360

ENSOARG000000     22015 AGGGTGGACCCCACCTCAGCACTCAGAGGCCTCCCCCAGGCAGACAGTCC  22064
                        ||||||||||||||||||||||||||||||||||||||||||||||||||
KP025765          25361 agggtggaccccacctcagcactcagaggcctcccccaggcagacagtcc  25410

ENSOARG000000     22065 AACATCAGCCAGTTCTTGGAGCACAGCTATTCTCTTATCAGACAGCATCA  22114
                        ||||||||||||||||||||||||||||||||||||||||||||||||||
KP025765          25411 aacatcagccagttcttggagcacagctattctcttatcagacagcatca  25460

ENSOARG000000     22115 AACTTTGCAAGTGGGGTTTTCCCTGAGAGCCCTCCTTTGGACCCCTCGGA  22164
```

```
                             ||||||||||||||||||||||||||||||||||||||||||||||||||
KP025765           25461 aactttgcaagtggggttttccctgagagccctcctttggacccctcgga  25510

ENSOARG000000      22165 CACTGAGATGTCTTCTGATCATCCAGAGAGCCCCATGTTGTCAGGTTGGG  22214
                             ||||||||||||||||||||||||||||||||||||||||||||||||||
KP025765           25511 cactgagatgtcttctgatcatccagagagccccatgttgtcaggttggg  25560

ENSOARG000000      22215 GCCCCTAGGCACTGTAACTCACTCAGAGCAGCCTGGAGTCTCTTCCTTAC  22264
                             ||||||||||||||||||||||||||||||||||||||||||||||||||
KP025765           25561 gcccctaggcactgtaactcactcagagcagcctggagtctcttccttac  25610

ENSOARG000000      22265 CCCTCCCAGGGCTGCATGTCCTCCCTTCTGGGTGTGGGGGAGCTGCCTCA  22314
                             ||||||||||||||||||||||||||||||||||||||||||||||||||
KP025765           25611 ccctcccagggctgcatgtcctcccttctgggtgtgggggagctgcctca  25660

ENSOARG000000      22315 CCTCTGCTCCAGGCTAACTGGCTCTTGTGCAACCTCCATCCCACCCTGGC  22364
                             ||||||||||||||||||||||||||||||||||||||||||||||||||
KP025765           25661 cctctgctccaggctaactggctcttgtgcaacctccatcccaccctggc  25710

ENSOARG000000      22365 GCACTCGGCTCGTGCAGGCCTCGGCCTGGCCTCGGCTGCAGGATCAGGCC  22414
                             ||||||||||||||||||||||||||||||||||||||||||||||||||
KP025765           25711 gcactcggctcgtgcaggcctcggcctggcctcggctgcaggatcaggcc  25760

ENSOARG000000      22415 TGCCTTTCTCCTCCCATCCTTGCCCAGGTGGCCAGGGCAGCCTTGGCAAG  22464
                             ||||||||||||||||||||||||||||||||||||||||||||||||||
KP025765           25761 tgcctttctcctcccatccttgcccaggtggccagggcagccttggcaag  25810

ENSOARG000000      22465 TGCCCGAATATCTCCACTTTTAAAATAGTTTTTATTTATTGTTTTGGTTG  22514
                             ||||||||||||||||||||||||||||||||||||||||||||||||||
KP025765           25811 tgcccgaatatctccacttttaaaatagtttttatttattgttttggttg  25860

ENSOARG000000      22515 TGCCAGGTCTTTGTGGGGGCATGCGGGATGATCAGTCTTTATCATGGCGC  22564
                             ||||||||||||||||||||||||||||||||||||||||||||||||||
KP025765           25861 tgccaggtctttgtgggggcatgcgggatgatcagtctttatcatggcgc  25910

ENSOARG000000      22565 GCAGGATCTTTTCAGTTGTGGCCTGTGGGATCTGGGGCTTCCCTGGTGGC  22614
                             ||||||||||||||||||||||||||||||||||||||||||||||||||
KP025765           25911 gcaggatcttttcagttgtggcctgtgggatctggggcttccctggtggc  25960

ENSOARG000000      22615 TCAGTGGTAGAGAGTCCACCTGCCAATGCGGGAGATCTGGGTTCAGTCCC  22664
                             ||||||||||||||||||||||||||||||||||||||||||||||||||
KP025765           25961 tcagtggtagagagtccacctgccaatgcgggagatctgggttcagtccc  26010

ENSOARG000000      22665 TGGGTCGGGAAGATCCCCTGGAGGAAGAAATGGCAACCCACTCCCGTGTT  22714
                             ||||||||||||||||||||||||||||||||||||||||||||||||||
KP025765           26011 tgggtcgggaagatcccctggaggaagaaatggcaacccactcccgtgtt  26060

ENSOARG000000      22715 CTTGCTTGGAAAATCCCATGGACAGAACAGCCTTGAGGGATCCATGGGGT  22764
                             ||||||||||||||||||||||||||||||||||||||||||||||||||
KP025765           26061 cttgcttggaaaatcccatggacagaacagccttgagggatccatggggt  26110

ENSOARG000000      22765 CCCTCGAGAGTCCATGGGGTCGCAAAAGACTTGGACACAACTTGGCTGCT  22814
                             ||||||||||||||||||||||||||||||||||||||||||||||||||
KP025765           26111 ccctcgagagtccatggggtcgcaaaagacttggacacaacttggctgct  26160

ENSOARG000000      22815 CAGCAACAACATGTAGGATCTAGTTCTTGGACCAAGGATTGAACCCGGGT  22864
                             ||||||||||||||||||||||||||||||||||||||||||||||||||
KP025765           26161 cagcaacaacatgtaggatctagttcttggaccaaggattgaacccgggt  26210

ENSOARG000000      22865 CCCCTGCATTGGGAGAGCAAAGTCTTAGCCCCTGGACCACCAGGAAGTCC  22914
                             ||||||||||||||||||||||||||||||||||||||||||||||||||
KP025765           26211 cccctgcattgggagagcaaagtcttagcccctggaccaccaggaagtcc  26260

ENSOARG000000      22915 CCAAATGTCTCCACTTTGGATTTCCCAACAGAGCATTTGGGGTTTGGTTG  22964
                             ||||||||||||||||||||||||||||||||||||||||||||||||||
```

```
KP025765          26261 ccaaatgtctccactttggatttcccaacagagcatttggggtttggttg  26310

ENSOARG000000     22965 GGGACGGTGAGGCCCAGGTGGTGAGGGCACTCTTGGCTCTGGAGCCAGAC  23014
                        ||||||||||||||||||||||||||||||||||||||||||||||||||
KP025765          26311 gggacggtgaggcccaggtggtgagggcactcttggctctggagccagac  26360

ENSOARG000000     23015 TCGCACAGATGCACGTGAATGCAGGACCTTTACTAACGGGTCAAGGTGAC  23064
                        ||||||||||||||||||||||||||||||||||||||||||||||||||
KP025765          26361 tcgcacagatgcacgtgaatgcaggacctttactaacgggtcaaggtgac  26410

ENSOARG000000     23065 ACGCGGCTGACTTGCGTCTAGTGTGTCTCTGCCACTCAACTTAGCGATGA  23114
                        ||||||||||||||||||||||||||||||||||||||||||||||||||
KP025765          26411 acgcggctgacttgcgtctagtgtgtctctgccactcaacttagcgatga  26460

ENSOARG000000     23115 ACCCCGTGGAGCCCTGGTGACACTTCTCTAAGCCAGTCACACCCGTCAGG  23164
                        ||||||||||||||||||||||||||||||||||||||||||||||||||
KP025765          26461 accccgtggagccctggtgacacttctctaagccagtcacacccgtcagg  26510

ENSOARG000000     23165 GCTTGGCCCGTGGCTGGGCATCAACAGTTGCTCGCTCCTTGCCTTGTTCC  23214
                        ||||||||||||||||||||||||||||||||||||||||||||||||||
KP025765          26511 gcttggcccgtggctgggcatcaacagttgctcgctccttgccttgttcc  26560

ENSOARG000000     23215 CCTGACCCCTGGGTGCAGTTCAAGGGCTGCGCGATGGTCCTGGGGCATCC  23264
                        ||||||||||||||||||||||||||||||||||||||||||||||||||
KP025765          26561 cctgacccctgggtgcagttcaagggctgcgcgatggtcctggggcatcc  26610

ENSOARG000000     23265 GAGACCAGGACCAGGCTGGAAGCTAGACGATCCCCCTGAGGCNNNNNNNN  23314
                        ||||||||||||||||||||||||||||||||||||||||||
KP025765          26611 gagaccaggaccaggctggaagctagacgatccccctgaggc--------  26652

ENSOARG000000     23315 NNNNNNNNNNNNNNNNNNNNNNNNNNNNNNNNNNNNNNNNNNNNNNNNNN  23364

KP025765          26653 --------------------------------------------------  26652

ENSOARG000000     23365 NNNNNNNNNNNNNNNNNNNNNNNNNNNNNNNNNNNNNNNNNN--------  23406

KP025765          26653 ------------------------------------------actctccc  26660

ENSOARG000000     23407 --------------------------------------------------  23406

KP025765          26661 tgccccaggacccattgggcccatgaagaaggtgctggtggacggcccca  26710

ENSOARG000000     23407 --------------------------------------------------  23406

KP025765          26711 agaagcggacgctgctcatcgagaacctgcgcgagtcccagccataccgc  26760

ENSOARG000000     23407 --------------------------------------------------  23406

KP025765          26761 tacacagtcaaggctcgcaacggggcaggctgggggcctgagcgggaggc  26810

ENSOARG000000     23407 --------------------------------------------------  23406

KP025765          26811 catcatcaacctggccacccagcccaagcgacccatgtccagtgagcagg  26860

ENSOARG000000     23407 --------------------------------------------------  23406

KP025765          26861 gggcagggaggcacagggtggacagggacgggcaggggcctgagatctgg  26910

ENSOARG000000     23407 --------------------------------------------------  23406

KP025765          26911 gccctggctcacctccccccaccccgcagtccccatcatccctgacatc  26960

ENSOARG000000     23407 --------------------------------------------------  23406

KP025765          26961 cccatcgtggacgcccagagtggggaggactacgagagcttcctcatgta  27010
```

```
ENSOARG000000  23407 --------------------------------------------------  23406

KP025765       27011 cagcgatgacgtgctccgctctccggccggcagccagaggcccagcgtct  27060

ENSOARG000000  23407 --------------------------------------------------  23406

KP025765       27061 ctgatgacaccggtgagtgggccggggatcccggcgaggatggtgggggt  27110

ENSOARG000000  23407 --------------------------------------------------  23406

KP025765       27111 cctgagtagagggtggggctgtcgctggtgtccagagacagcaaaggggc  27160

ENSOARG000000  23407 --------------------------------------------------  23406

KP025765       27161 cagtgaccactgggcaagaagatcaagactgaaagtcccctctctgcaca  27210

ENSOARG000000  23407 --------------------------------------------------  23406

KP025765       27211 atcaacgatttgacgggcaccattctggctctgggtacaaaggacaaagc  27260

ENSOARG000000  23407 --------------------------------------------------  23406

KP025765       27261 cgacatggctcataggactggtgtgcacgtctctgtgtgaggacagacag  27310

ENSOARG000000  23407 --------------------------------------------------  23406

KP025765       27311 acagacatgggtttgggacacatgcacacgggctcgtgcacatgagactg  27360

ENSOARG000000  23407 --------------------------------------------------  23406

KP025765       27361 gaaacagctgtgagagtggaaggctcaccagctgtgccatggagtcagga  27410

ENSOARG000000  23407 --------------------------------------------------  23406

KP025765       27411 cacctcccagggacagggctacaggcgaggaggcaagcctggtccgggag  27460

ENSOARG000000  23407 --------------------------------------------------  23406

KP025765       27461 ccccagaatagcgattggcttgggggcccccagagcagtgctccttgcac  27510

ENSOARG000000  23407 --------------------------------------------------  23406

KP025765       27511 tggccctgctccacttccctcttggattcctgtacagtgtgatgcaaaga  27560

ENSOARG000000  23407 --------------------------------------------------  23406

KP025765       27561 gcccccgactgggactcctggggtctattttagctctggcttcactgccg  27610

ENSOARG000000  23407 --------------------------------------------------  23406

KP025765       27611 acggcttcccttgtagctcagttggtaaagagtctgcctgcaatgcagga  27660

ENSOARG000000  23407 --------------------------------------------------  23406

KP025765       27661 gacctgggttcaattcctgggttggaaagatcccctggagaaggaaatgg  27710

ENSOARG000000  23407 --------------------------------------------------  23406

KP025765       27711 caacccactccagtattctggcctggagaaccccatggacagaggagcct  27760

ENSOARG000000  23407 ------------TCCATGGGGTCTATGGGGCAAGAGTTGGACAGGACTTA  23444
                                 ||||||||||||||||||||||||||||||||||||||
KP025765       27761 ggcaggccacagtccatggggtctatggggcaagagttggacaggactta  27810
```

```
ENSOARG000000  23445 GTGACTAAACCACCACTGTGGGAATTCAGATGAATCACATACATTCTCTG  23494
                     ||||||||||||||||||||||||||||||||||||||||||||||||||
KP025765       27811 gtgactaaaccaccactgtgggaattcagatgaatcacatacattctctg  27860

ENSOARG000000  23495 CCTCAGTTTTCTGATCTGTGAAATGGGATGCCAATCATACTAGGCTGAGT  23544
                     ||||||||||||||||||||||||||||||||||||||||||||||||||
KP025765       27861 cctcagttttctgatctgtgaaatgggatgccaatcatactaggctgagt  27910

ENSOARG000000  23545 AAAAATCCAGCAGGATTTGTATTTCCTTTTTATATAATTTATTTTCGGCT  23594
                     ||||||||||||||||||||||||||||||||||||||||||||||||||
KP025765       27911 aaaaatccagcaggatttgtatttcctttttatataatttattttcggct  27960

ENSOARG000000  23595 GTGCTGGGTCTTCATTGCTATGCAGGCTTCTCATCCCGGTGGCTTCTCTT  23644
                     ||||||||||||||||||||||||||||||||||||||||||||||||||
KP025765       27961 gtgctgggtcttcattgctatgcaggcttctcatcccggtggcttctctt  28010

ENSOARG000000  23645 ACTGTGGAGCAAGACGGGCTCTAGGGCTTAGTAGTTGCAACTCCTGGGCT  23694
                     ||||||||||||||||||||||||||||||||||||||||||||||||||
KP025765       28011 actgtggagcaagacgggctctagggcttagtagttgcaactcctgggct  28060

ENSOARG000000  23695 CTAGAGCAGTTACCCCGCGGCATGCAGGATCTTCCCTGTTCAGTTCAGTT  23744
                     ||||||||||||||||||||||||||||||||||||||||||||||||||
KP025765       28061 ctagagcagttaccccgcggcatgcaggatcttccctgttcagttcagtt  28110

ENSOARG000000  23745 CAGTTTGGTTCCGTCACAGACCAGGGATCAAACCTGTTTTTCCCCCATGG  23794
                     ||||||||||||||||||||||||||||||||||||||||||||||||||
KP025765       28111 cagtttggttccgtcacagaccagggatcaaacctgtttttcccccatgg  28160

ENSOARG000000  23795 GGAGGTAGATTCTTTACCATTGAGCCATCAGGGAAGCCCCAGGATTTGTA  23844
                     ||||||||||||||||||||||||||||||||||||||||||||||||||
KP025765       28161 ggaggtagattctttaccattgagccatcagggaagccccaggatttgta  28210

ENSOARG000000  23845 TTTTCATCCTTCTTTACCTACATCAGGCTTCCATGGAGTCAAGCTAGGGT  23894
                     ||||||||||||||||||||||||||||||||||||||||||||||||||
KP025765       28211 ttttcatccttctttacctacatcaggcttccatggagtcaagctagggt  28260

ENSOARG000000  23895 TCAGGGGTCTTGAGGGTTTTGAGGGTCTGGAGTAGAGAGCAGCTGGTATG  23944
                     ||||||||||||||||||||||||||||||||||||||||||||||||||
KP025765       28261 tcaggggtcttgagggttttgagggtctggagtagagagcagctggtatg  28310

ENSOARG000000  23945 AATCAGGTTCCTCAGCTTGTCGGCACCACACAGATCTACTCCGCCCGCTG  23994
                     ||||||||||||||||||||||||||||||||||||||||||||||||||
KP025765       28311 aatcaggttcctcagcttgtcggcaccacacagatctactccgcccgctg  28360

ENSOARG000000  23995 GAACACGCTGGGAGGCAGGCTGGGGAGACATCACTCCCTCCCTCATCCAT  24044
                     ||||||||||||||||||||||||||||||||||||||||||||||||||
KP025765       28361 gaacacgctgggaggcaggctggggagacatcactccctccctcatccat  28410

ENSOARG000000  24045 ACGGGATGCAGGCTCAGAGCAGAGGCCTCACGGCTCGCGGCACAGCCAGG  24094
                     ||||||||||||||||||||||||||||||||||||||||||||||||||
KP025765       28411 acgggatgcaggctcagagcagaggcctcacggctcgcggcacagccagg  28460

ENSOARG000000  24095 GCTCTGGTCCAGAGGGTCTTCCCTCGCCAGCTGCGGCCCAGGCCGCCCTC  24144
                     ||||||||||||||||||||||||||||||||||||||||||||||||||
KP025765       28461 gctctggtccagagggtcttccctcgccagctgcggcccaggccgccctc  28510

ENSOARG000000  24145 TAACACCTC-----------------------------------------  24153
                     |||||||||
KP025765       28511 taacacctcggggctgagggtcccgcctgccgccgggctcccgcaccgcc  28560

ENSOARG000000  24154 --------------------------------------------------  24153

KP025765       28561 gcacacccagatcgcagtgccgggggaccaccggctttttctccgcgccc  28610

ENSOARG000000  24154 --------------------------------------------------  24153
```

```
KP025765         28611 ctggcgccccctggcggtgccaacgcggcccttcgttgttcctaaggctg  28660

ENSOARG000000    24154 --------------------------------------------------  24153

KP025765         28661 cggctggaagttcgagcccctgctgggggaggagctggacctgcggcgcg  28710

ENSOARG000000    24154 --------------------------------------------------  24153

KP025765         28711 tcacgtggcggctgcccccggagctcatcccgcgcctgtccgctggcagc  28760

ENSOARG000000    24154 --------------------------------------------------  24153

KP025765         28761 cggcgctcctccgactccgggcccccgggagacggcaccgccgatggggg  28810

ENSOARG000000    24154 --------------------------------------------------  24153

KP025765         28811 cgcgcggggcgccagcggggagggaggtgaccggcatgcccccacccgca  28860

ENSOARG000000    24154 --------------------------------------------------  24153

KP025765         28861 cccacctcggctccagccagagcccaccgcagccaggctgccttgcccgg  28910

ENSOARG000000    24154 --------------------------------------------------  24153

KP025765         28911 tgaccccagagccaggggacctccccagggcggggccgactgctggggct  28960

ENSOARG000000    24154 --------------------------------------------------  24153

KP025765         28961 gcaaggccctggcgaaggtcgggtccgtgggggcctcctgccaggccagg  29010

ENSOARG000000    24154 -------TGTTCCAGAAGCCTGCGGGCGTTGGGGGGGCTGTGGACTCCTC  24196
                              |||||||||||||||||||||||||||||||||||||||||||
KP025765         29011 ggacctgtgttccagaagcctgcgggcgttgggggggctgtggactcctc  29060

ENSOARG000000    24197 CAGGGGCCTCTGTCCCTGGTGGGCAGAGGGAGGCTGGGTGTTTTGATGCC  24246
                       ||||||||||||||||||||||||||||||||||||||||||||||||||
KP025765         29061 caggggcctctgtccctggtgggcagagggaggctgggtgttttgatgcc  29110

ENSOARG000000    24247 AGGGGGCCCCCGGTAGGTAGGTAACCCCTGGAACTCCCAGGGCTGCAGCA  24296
                       |||||| |||||||||||||||||||||||||||||||||||||||||||
KP025765         29111 aggggg-ccccggtaggtaggtaacccctggaactcccagggctgcagca  29159

ENSOARG000000    24297 GCCCGCCCCTCGGGGCTCCGGGGTCTGCGGCCGGGCCTGTGGGAGTGACC  24346
                       ||||||||||||||||||||||||||||||||||||||||||||||||||
KP025765         29160 gcccgcccctcggggctccggggtctgcggccgggcctgtgggagtgacc  29209

ENSOARG000000    24347 CTGCCCTGTCCCCGCAGAGCACCTGGTGAACGGGCGAATGGACTTTGCCT  24396
                       ||||||||||||||||||||||||||||||||||||||||||||||||||
KP025765         29210 ctgccctgtccccgcagagcacctggtgaacgggcgaatggactttgcct  29259

ENSOARG000000    24397 TCCCTGGCAGCGCCAACTCCCTGCACAGGATGACCGTGAGCACCGCCGCC  24446
                       ||||||||||||||||||||||||||||||||||||||||||||||||||
KP025765         29260 tccctggcagcgccaactccctgcacaggatgaccgtgagcaccgccgcc  29309

ENSOARG000000    24447 CACGGCGCCCACCTGAGCCCGCAGCTGTCCCACCGCATGCTGAGCACCTC  24496
                       ||||||||||||||||||||||||||||||||||||||||||||||||||
KP025765         29310 cacggcgcccacctgagcccgcagctgtcccaccgcatgctgagcacctc  29359

ENSOARG000000    24497 GTCCACCCTCACGCGGGACTACCACTCGCTGACGCGCACGGAGCACTCGC  24546
                       ||||||||||||||||||||||||||||||||||||||||||||||||||
KP025765         29360 gtccaccctcacgcgggactaccactcgctgacgcgcacggagcactcgc  29409

ENSOARG000000    24547 ACTCCGCCACGCTGCCCAGGGACTACTCCACCCTCACCTCCCTCTCCTCC  24596
                       ||||||||||||||||||||||||||||||||||||||||||||||||||
```

```
KP025765         29410 actccgccacgctgcccagggactactccaccctcacctccctctcctcc  29459

ENSOARG000000    24597 CAGAGTGAGTGCCGCCTCCTCCCTGGCCCTCCGGCCAGGCCTTCCCCCCT  24646
                       ||||||||||||||||||||||||||||||||||||||.||||||.|.||
KP025765         29460 cagagtgagtgccgcctcctccctggccctccggccagtccttcctctct  29509

ENSOARG000000    24647 TCCCCCCCCCTGCGGCGTGACCAGGGCCTGCTGTCCCACGCTGCCCCCCG  24696
                       |||..|.||..|.||||||| |||||.|||||||||||||||||||||||
KP025765         29510 tccagctcctggaggcgtga-caggggctgctgtcccacgctgccccccg  29558

ENSOARG000000    24697 TCCAACACGCACACCTGCCCCAGCTCACTCATGTTTTGTCCTGCCCTAGG  24746
                       ||||||||||||||||||||||||||||||||||||||||||||||||||
KP025765         29559 tccaacacgcacacctgccccagctcactcatgttttgtcctgccctagg  29608

ENSOARG000000    24747 CCTCCCTCCCATCTGGGAACACGGGAGGAGCAGGCTTCCATTGTCCTGGG  24796
                       ||||||||||||||||||||||||||||||||||||||||||||||||||
KP025765         29609 cctccctcccatctgggaacacgggaggagcaggcttccattgtcctggg  29658

ENSOARG000000    24797 CCCTGGGGTCCCGGAGTCGGGCTCAGATGAACGGGGCCCCCCGCTCCGAG  24846
                       ||||||||||||||||||||||||||||||||||||||||||||||||||
KP025765         29659 ccctggggtcccggagtcgggctcagatgaacggggccccccgctccgag  29708

ENSOARG000000    24847 GACTCAAGAGACTCTATAATCCTGGCTGGGCAGCCAGCAGCGCCCTCGTG  24896
                       ||||||||||||||||||||||||||||||||||||||||||||||||||
KP025765         29709 gactcaagagactctataatcctggctgggcagccagcagcgccctcgtg  29758

ENSOARG000000    24897 GGGCCCAGGTAGTACAGGGGTGGCCGTCCTGCGTCTCCATGGCCTTCCAG  24946
                       ||||||||||||||||||||||||||||||||||||||||||||||||||
KP025765         29759 gggcccaggtagtacaggggtggccgtcctgcgtctccatggccttccag  29808

ENSOARG000000    24947 CTCCATGTCACCATCCTCCCACCATCAGCACTGCCCACGCCTCCTCACCC  24996
                       ||||||||||||||||||||||||||||||||||||||||||||||||||
KP025765         29809 ctccatgtcaccatcctcccaccatcagcactgcccacgcctcctcaccc  29858

ENSOARG000000    24997 TCCATTCCATCCACATCCATGCTGGTGCCTAAAGCGGCTGTCCTCCTGCG  25046
                       ||||||||||||||||||||||||||||||||||||||||||||||||||
KP025765         29859 tccattccatccacatccatgctggtgcctaaagcggctgtcctcctgcg  29908

ENSOARG000000    25047 GTGCTGGGCTGGGGCTGGGGCAAGAGAGGGGCCAGCTGAGGGTGGGGGGC  25096
                       ||||||||||||||||||||||||||||||||||||||||||||||||||
KP025765         29909 gtgctgggctggggctggggcaagagaggggccagctgagggtggggggc  29958

ENSOARG000000    25097 CGGCCACTCAAGCGGAGCCTGCACCCACCTGAGTGTGGTGGGGAGGCACT  25146
                       ||||||||||||||||||||||||||||||||||||||||||||||||||
KP025765         29959 cggccactcaagcggagcctgcacccacctgagtgtggtggggaggcact  30008

ENSOARG000000    25147 GGGCGGCTTAGAGAGGGTGCTGCAGGGCTGAGTGGGCTGCCCGGCCAGCA  25196
                       ||||||||||||||||||||||||||||||||||||||||||||||||||
KP025765         30009 gggcggcttagagagggtgctgcagggctgagtgggctgcccggccagca  30058

ENSOARG000000    25197 GTCAGGGAGGTGCGGCTGAGAACCAGGGACTCCTGCCCAGACACCCCCGC  25246
                       ||||||||||||||||||||||||||||||||||||||||||||||||||
KP025765         30059 gtcagggaggtgcggctgagaaccagggactcctgcccagacacccccgc  30108

ENSOARG000000    25247 TGGGGGCTGACTGTGGCCTCCCCCATCTGCAGGCTCCCGCCTGGCTGCGG  25296
                       ||||||||||||||||||||||||||||||||||||||||||||||||||
KP025765         30109 tgggggctgactgtggcctcccccatctgcaggctcccgcctggctgcgg  30158

ENSOARG000000    25297 GCG---------------GCCGCCTGGTGTTCTCCGCCCTCGGACCCACG  25331
                       |||               .|||||||||||||||||||||||||||||||
KP025765         30159 gcgtgcccaacacgcccacccgcctggtgttctccgccctcggacccacg  30208

ENSOARG000000    25332 TCTCTGAAAGTGAGCTGGCAGGAGCCGCAGTGTGAGCGTGCGCTGCAGGG  25381
                       ||||||||||||||||||||||||||||||||||||||||||||||||||
KP025765         30209 tctctgaaagtgagctggcaggagccgcagtgtgagcgtgcgctgcaggg  30258
```

```
ENSOARG000000       25382 CTACAGTGTGGAGTACCAGCTGCTGAACGGCGGTGAGGCCTGGCTGCTGG  25431
                          ||||||||||||||||||||||||||||||||||||||||||||||||||
KP025765            30259 ctacagtgtggagtaccagctgctgaacggcggtgaggcctggctgctgg  30308

ENSOARG000000       25432 CGGGCTGACACACAGGGGCTCCGCGGGGC---------CCCCGCCCGAGC  25472
                          |||||||||||||||||||||    ||||         |||.| ||||||
KP025765            30309 cgggctgacacacaggggctc----gggctgtctggagcccag-ccgagc  30353

ENSOARG000000       25473 CGCTTGACCACTGATGCCCCCGCTGCCACCCCCTGCCTCTCTGAGAGGGC  25522
                          ||||||||||||||||||||||||||||||||||||||||||||||||||
KP025765            30354 cgcttgaccactgatgcccccgctgccaccccctgcctctctgagagggc  30403

ENSOARG000000       25523 CTTTCCCTTCCGAGCCCCCCACTGTCCCCTTCACTGCGTCCACTCTTCCG  25572
                          ||||||||||||||||||||||||||||||||||||||||||||||||||
KP025765            30404 ctttcccttccgagccccccactgtccccttcactgcgtccactcttccg  30453

ENSOARG000000       25573 CTGTATCCTCATCCGTCTCCCTGGGCCTCGCTCCGTATCACTTCCCCCGC  25622
                          ||||||||||||||||||||||||||||||||||||||||||||||||||
KP025765            30454 ctgtatcctcatccgtctccctgggcctcgctccgtatcacttcccccgc  30503

ENSOARG000000       25623 GAGGTTTTGCTTAGGGACCCAGGGCCTCTCTGCTGGGCCTATGAGAAGTG  25672
                          ||||||||||||||||||||||||||||||||||||||||||||||||||
KP025765            30504 gaggttttgcttagggacccagggcctctctgctgggcctatgagaagtg  30553

ENSOARG000000       25673 GCCCTTTTCTGGGCAGGCATGACCTGGGCCCATAAACAGCTATCATACAC  25722
                          ||||||||||||||||||||||||||||||||||||||||||||||||||
KP025765            30554 gcccttttctgggcaggcatgacctgggcccataaacagctatcatacac  30603

ENSOARG000000       25723 AGATGAGATTTCAGATTCCACTCCTTGTCTAGTGCACAACCTGAACCACC  25772
                          ||||||||||||||||||||||||||||||||||||||||||||||||||
KP025765            30604 agatgagatttcagattccactccttgtctagtgcacaacctgaaccacc  30653

ENSOARG000000       25773 AACCACACCACACAAGGCCGCCCCCACCTGTACCCCAACCACAGCC----  25818
                          ||||||||||||||||||||||||||||||||||||||||||||||    
KP025765            30654 aaccacaccacacaaggccgcccccacctgtaccccaaccacagccggtc  30703

ENSOARG000000       25819 --GGCGGGGGGAGGACCAGGTCTGGGCTGTGGCCTAGGGGAGGGGCGGGA  25866
                            |||.||||||||||||||||||||||||||||||||||||||||||||
KP025765            30704 ctggctgggggaggaccaggtctgggctgtggcctaggggaggggcggga  30753

ENSOARG000000       25867 TCAAGCCGGGGCTAGGGATGAGGAATGAGGCTGCTCTGCTGTCTGCCCCC  25916
                          ||||||||||||||||||||||||||||||||||||||||||||||||||
KP025765            30754 tcaagccggggctagggatgaggaatgaggctgctctgctgtctgccccc  30803

ENSOARG000000       25917 GTCGCCCACCCAGGTGAGCTGCATCGCCTCAACATCCCCAGCCCCAGCCA  25966
                          ||||||||||||||||||||||||||||||||||||||||||||||||||
KP025765            30804 gtcgcccacccaggtgagctgcatcgcctcaacatccccagccccagcca  30853

ENSOARG000000       25967 GACCTCGGTGGTGGTGGAGGACCTCCTGCCCAACCACTCGTATGTGTTCC  26016
                          ||||||||||||||||||||||||||||||||||||||||||||||||||
KP025765            30854 gacctcggtggtggtggaggacctcctgcccaaccactcgtatgtgttcc  30903

ENSOARG000000       26017 GCGTGCGGGCCCAGAGCCAGGAGGGCTGGGGCCCAGAGCGCGAGGGTGTC  26066
                          ||||||||||||||||||||||||||||||||||||||||||||||||||
KP025765            30904 gcgtgcgggcccagagccaggagggctggggcccagagcgcgagggtgtc  30953

ENSOARG000000       26067 ATCACCATTGAGTCCCAGGTGCACCCGCAGAGCCCGCTCTGCCCCCTGCC  26116
                          ||||||||||||||||||||||||||||||||||||||||||||||||||
KP025765            30954 atcaccattgagtcccaggtgcacccgcagagcccgctctgccccctgcc  31003

ENSOARG000000       26117 GGGTGAGTTGCCACCCCCACACCCGCGGGTGCCCCTGCCTGGCCCTCAGG  26166
                          ||||||||||||||||||||||||||||||||||||||||||||||||||
KP025765            31004 gggtgagttgccacccccacacccgcgggtgcccctgcctggccctcagg  31053
```

```
ENSOARG000000  26167 TACCCACCTGTCCCCTGACAAGCTTCCTCGACTGCCCCCAGGCTCCGCCT  26216
                     |||||||||||||||||||||||||||||||||||||||| |||||||||
KP025765       31054 tacccacctgtcccctgacaagcttcctcgactgccccca-gctccgcct  31102

ENSOARG000000  26217 TCACTTTGAGCACACCCAGTGCCCCGGGCCCACTGGTGTTCACTGCCCTG  26266
                     ||||||||||||||||||||||||||||||||||||||||||||||||||
KP025765       31103 tcactttgagcacacccagtgccccgggcccactggtgttcactgccctg  31152

ENSOARG000000  26267 AGCCCCGACTCACTGCAGCTGAGCTGGGAGCGGCCCCGCAGGCCGGATGG  26316
                     ||||||||||||||||||||||||||||||||||||||||||||||||||
KP025765       31153 agccccgactcactgcagctgagctgggagcggccccgcaggccggatgg  31202

ENSOARG000000  26317 CGACATTCTGGGCTACCTGGTGACGTGTGAGATGGCCCATGGAGGAGGTG  26366
                     ||||||||||||||||||||||||||||||||||||||||||||||||||
KP025765       31203 cgacattctgggctacctggtgacgtgtgagatggcccatggaggaggtg  31252

ENSOARG000000  26367 CTGCCCACCCCGGGTTGGGGGGCGGGGGGGGGGGGGGG--GGGGAGCCGC  26414
                     |||||||||||||||||||||||||||||||||.|.||  .|||||||||
KP025765       31253 ctgcccaccccgggttggggggcggggggggggtgtggacagggagccgc  31302

ENSOARG000000  26415 AGAGGCTGAAAGGCATCTTCCCTGCCCAGAGCCAGCCACCACGTTCCTGG  26464
                     ||||||||||||||||||||||||||||||||||||||||||||||||||
KP025765       31303 agaggctgaaaggcatcttccctgcccagagccagccaccacgttcctgg  31352

ENSOARG000000  26465 TTGACGGCGACAGCCCCGAGAGCCGGCTGACCGTGCCCGGCCTCAGCGAG  26514
                     ||||||||||||||||||||||||||||||||||||||||||||||||||
KP025765       31353 ttgacggcgacagccccgagagccggctgaccgtgcccggcctcagcgag  31402

ENSOARG000000  26515 AACGTGCCCTACAAGTTCAAGGTGCAGGCAAAGACCACCCAAGGCTTCGG  26564
                     ||||||||||||||||||||||||||||||||||||||||||||||||||
KP025765       31403 aacgtgccctacaagttcaaggtgcaggcaaagaccacccaaggcttcgg  31452

ENSOARG000000  26565 GCCGGAGCGTGAAGGAATCATCACCATCGAGTCCCAGGATGGAGGCAGGC  26614
                     ||||||||||||||||||||||||||||||||||||||||||||||||||
KP025765       31453 gccggagcgtgaaggaatcatcaccatcgagtcccaggatggaggcaggc  31502

ENSOARG000000  26615 TTCTTTGCCCCTTCCCCAGCCCTGCCCCTTCTCTGGCCACACCTTCTCCT  26664
                     ||||||||||||||||||||||||||||||||||||||||||||||||||
KP025765       31503 ttctttgccccttccccagccctgccccttctctggccacaccttctcct  31552

ENSOARG000000  26665 GGCACCATCCTCTCCCTGCGCCCCC--------------GGACCCTTCCC  26700
                     |||||||||||||..||||||||||              |||||||||||
KP025765       31553 ggcaccatcctctaactgcgcccccacccaccctctccaggacccttccc  31602

ENSOARG000000  26701 ACAGCTGGGCGGCCACTTGGGACTCTTCCAGCACCCAGCATCAGGGGAAT  26750
                     ||||||||||||||||||||||||||||||||||||||||||||||||||
KP025765       31603 acagctgggcggccacttgggactcttccagcacccagcatcaggggaat  31652

ENSOARG000000  26751 ACAGCAGCATCACCACCCACTCCAGCACCACTGAGCCCTTCCTACTGGGT  26800
                     ||||||||||||||||||||||||||||||||||||||||||||||||||
KP025765       31653 acagcagcatcaccacccactccagcaccactgagcccttcctactgggt  31702

ENSOARG000000  26801 GAGTTGCCCAGTGGGGCCCCCTGGCTCTTCCCAGGAAACACAGAGCTAAC  26850
                     ||||||||||||||||||||||||||||||||||||||||||||||||||
KP025765       31703 gagttgcccagtggggccccctggctcttcccaggaaacacagagctaac  31752

ENSOARG000000  26851 AGAGGAAGGGGTCAGGGC-GGGGGGGGGGGACAGGCAGGACCCAG-----  26894
                     |||||||||||||||||| |||||||||||||||.||||||||||     
KP025765       31753 agaggaaggggtcagggcggggggggggggacagtcaggacccaggggta  31802

ENSOARG000000  26895 --------------TCTTCCCCTGCAGATGGACTGACCCTGGGCTCCCAG  26930
                                   ||||||||||||||||||||||||||||||||||||
KP025765       31803 ggtccttcaacgcttcttcccctgcagatggactgaccctgggctcccag  31852

ENSOARG000000  26931 CACCTGGAAGCAAGTGGCTCCCTCACCCGGCATGTGACACAGGAGTTTGT  26980
```

```
                                 ||||||||||||||||||||||||||||||||||||||||||||||||||
KP025765            31853 cacctggaagcaagtggctccctcacccggcatgtgacacaggagtttgt  31902

ENSOARG000000       26981 GAGCCGGACGCTGACCACCAGTGGGACCCTCAGCACCCACGTGGACCAAC  27030
                                 ||||||||||||||||||||||||||||||||||||||||||||||||||
KP025765            31903 gagccggacgctgaccaccagtgggaccctcagcacccacgtggaccaac  31952

ENSOARG000000       27031 AGTTCTTCCAGACCTGAGCTCCCCCAACCCTACACACACACCCCGCTCCA  27080
                                 ||||||||||||||||||||||||||||||||||||||||||||||||||
KP025765            31953 agttcttccagacctgagctcccccaaccctacacacacaccccgctcca  32002

ENSOARG000000       27081 CCCCAACCCCAGGCGCTGCCTCAGCTACTCCATCCTTGGACTCCTGGTGG  27130
                                 ||||||||||||||||||||||||||||||||||||||||||||||||||
KP025765            32003 ccccaaccccaggcgctgcctcagctactccatccttggactcctggtgg  32052

ENSOARG000000       27131 CCTGGCCCAGCCACATGCTGATCACAAGGCCGCTGCCTCTGGCCACAGTG  27180
                                 ||||||||||||||||||||||||||||||||||||||||||||||||||
KP025765            32053 cctggcccagccacatgctgatcacaaggccgctgcctctggccacagtg  32102

ENSOARG000000       27181 CAGGGGCTCAGTGTCCTCTGGACGGCATGGAGGGGGCAGAGGTCCCGGAA  27230
                                 ||||||||||||||||||||||||||||||||||||||||||||||||||
KP025765            32103 caggggctcagtgtcctctggacggcatggagggggcagaggtcccggaa  32152

ENSOARG000000       27231 GGCCCTTCTGGCTGCCCCCCCTGCCCCCGGCCCCAGCCCCTTTGTAACCA  27280
                                 ||||||||||||||||||||||||||||||||||||||||||||||||||
KP025765            32153 ggcccttctggctgccccccctgcccccggccccagcccctttgtaacca  32202

ENSOARG000000       27281 AAGAGCTGGACCCAGCATGGCAAGGGCTTGGCTTTGTTCTGCACTATAAT  27330
                                 ||||||||||||||||||||||||||||||||||||||||||||||||||
KP025765            32203 aagagctggacccagcatggcaagggcttggctttgttctgcactataat  32252

ENSOARG000000       27331 AAAGGATTTCGCTACTGCTTGGCTCTGCCCTGACCGTGTCTCTAGGCCCT  27380
                                 ||||||||||||||||||||||||||||||||||||||||||||||||||
KP025765            32253 aaaggatttcgctactgcttggctctgccctgaccgtgtctctaggccct  32302

ENSOARG000000       27381 GTCCAGAGCAGGGAGTGGTTCTCACACCCCCCTTTTCTGATGCCCCAGCA  27430
                                 ||||||||||||||||||||||||||||||||||||||||||||||||||
KP025765            32303 gtccagagcagggagtggttctcacaccccccttttctgatgccccagca  32352

ENSOARG000000       27431 CACATACACCTCTTGAGGCAGGCAGGCATCAGAGTCAGATACAAGTTTAT  27480
                                 ||||||||||||||||||||||||||||||||||||||||||||||||||
KP025765            32353 cacatacacctcttgaggcaggcaggcatcagagtcagatacaagtttat  32402

ENSOARG000000       27481 TGAGCACCCAGACAGGAGGGGAGGCCCGCCGTGTGCTGTCCCGGCGATGG  27530
                                 ||||||||||||||||||||||||||||||||||||||||||||||||||
KP025765            32403 tgagcacccagacaggaggggaggcccgccgtgtgctgtcccggcgatgg  32452

ENSOARG000000       27531 CCTCATAAGCGCAGCACCTTGGCGCCGTCAGCCGCCTGGGAGAGGTAGAA  27580
                                 ||||||||||||||||||||||||||||||||||||||||||||||||||
KP025765            32453 cctcataagcgcagcaccttggcgccgtcagccgcctgggagaggtagaa  32502

ENSOARG000000       27581 GGTGGCGGTCCCATGGTACTGCTCCTGCCGGGGGATGGGGGGTCTGAGCT  27630
                                 ||||||||||||||||||||||||||||||||||||||||||||||||||
KP025765            32503 ggtggcggtcccatggtactgctcctgccgggggatggggggtctgagct  32552

ENSOARG000000       27631 CGGGGGGCCCTGGGAGC---------------------------------  27647
                                 |||||||||||||||||
KP025765            32553 cgggggggccctgggagcccatccctcctgctcccttgccctcccctcccc  32602

ENSOARG000000       27648 --------------------------------------------------  27647

KP025765            32603 tcgactccagcactgccaacctggatgtgctgcatcacccgaggagcggc  32652

ENSOARG000000       27648 --------------------------------------------------  27647
```

```
KP025765         32653 ggaggcctccagcagggtcaccgtgcagcccccaaagccaccgcccgtca  32702

ENSOARG000000    27648 --------------------------------------------------  27647

KP025765         32703 tgcggctgccataaacccccggcgcggagagcgcggcctccaccagctga  32752

ENSOARG000000    27648 --------------------------------------------------  27647

KP025765         32753 tccagctccgggcagctcacctcgtagtcgtctctgtggagagaatacgg  32802

ENSOARG000000    27648 -----------------------------------  27647

KP025765         32803 agcctggagccagccccagctgcaacacgtgtgct  32837


#---------------------------------------
#---------------------------------------
```
