# Supplementary material for: Combining GWAS and RNA-Seq Approaches for Detection of the Causal Mutation for Hereditary Junctional Epidermolysis Bullosa in Sheep
Source: PLoS One. 2015 May 8;10(5):e0126416. doi: 10.1371/journal.pone.0126416 (PMC4425408; doi:10.1371/journal.pone.0126416)
Supplement: S2 Fig — Examples for fluorescent fragment length analysis and RFLP used in carrier detection are displayed. (PDF) [file pone.0126416.s002.pdf]

File S2. Examples of rapid genotyping methods for the detection of JEB-carriers.

1.- Fluorescent fragment length analysis. A 243 bp PCR fragment including the regions carrying the mutation was amplified. Wild-type animals shows a 243 bp fragment (+/+), JEB-affected animals (EB/EB) display a unique 239 bp fragment (EB/EB) and both fragments could be identified in carriers (+/EB).

EB/EB

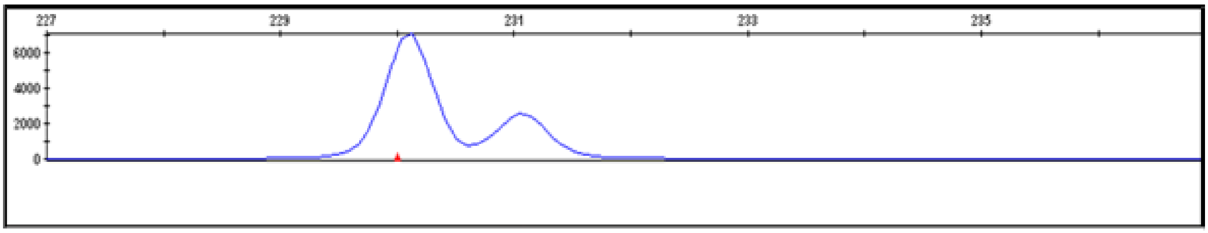

EB/+

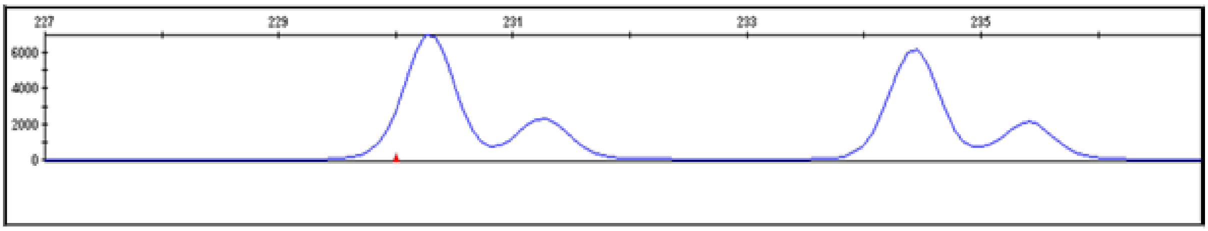

+/+

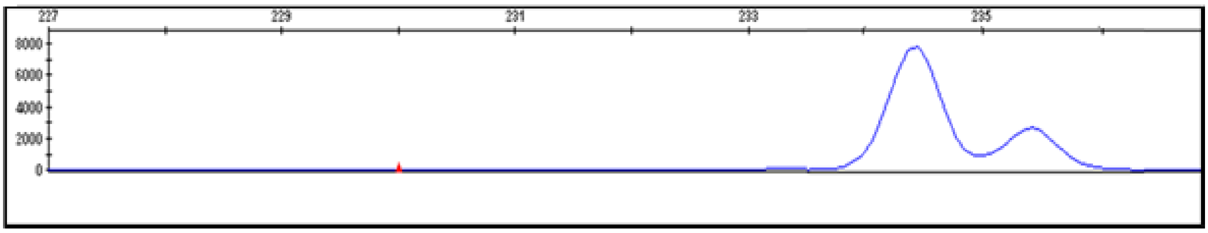

2.- **Restriction fragment length polymorphism analysis**.

Proposed endonucleases for the analysis in wild-type and mutated sequences.

A.) Restriction map analysis of wild-type sequence (*PshAI*)

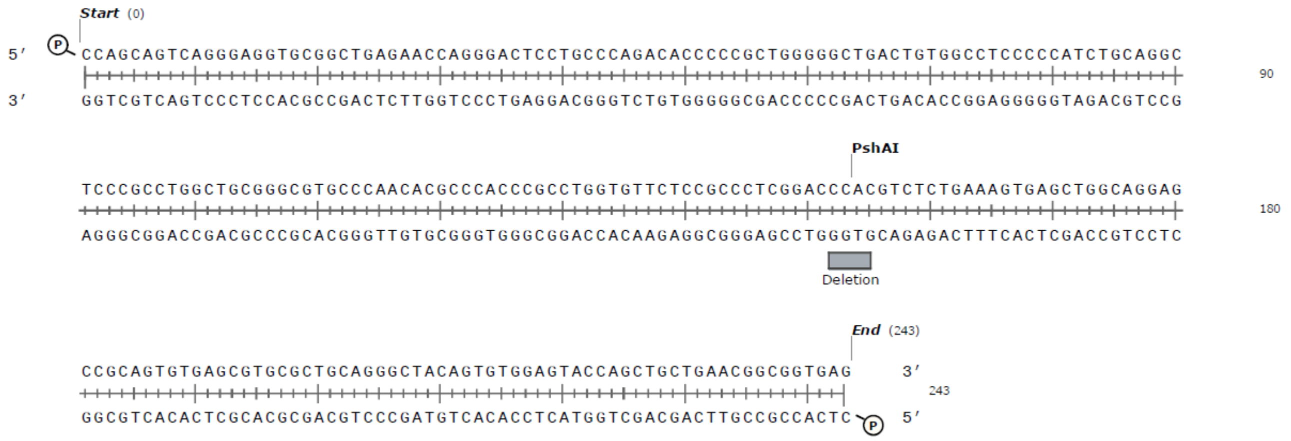

B.) Restriction map analysis of mutated sequence (*AcyI, Hin1I, Hsp92I and BsaHI*)

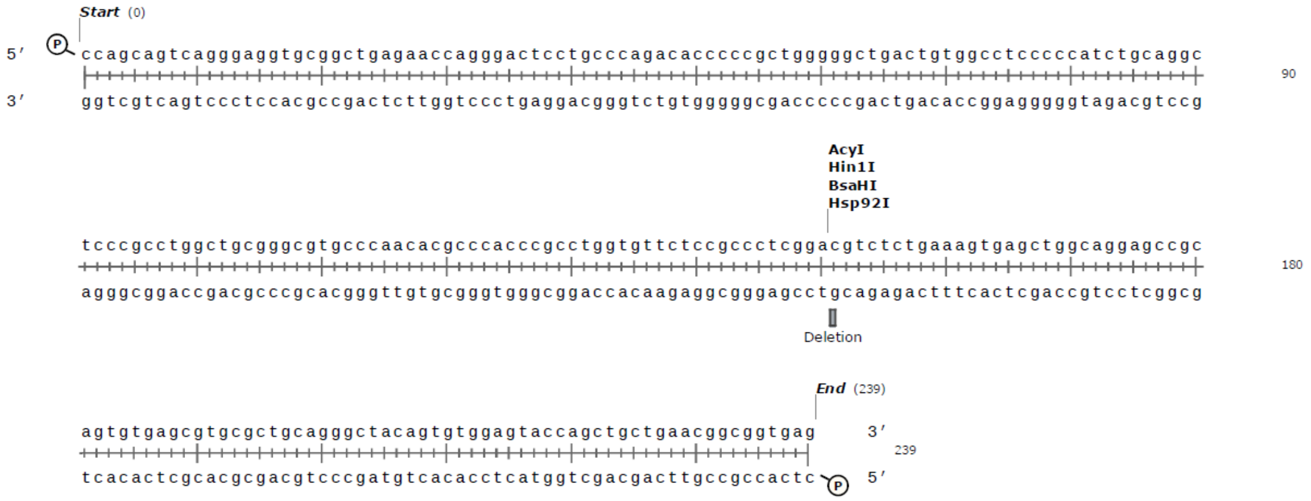

C). Agarose gel showing the RFLP produced with *PshAI* (cut wild-type sequence) and *AcyI* (cut mutated sequence)

PshAI digestion

MW: ϕX174DNA – *HinfI* Digest
Lane 1: Wild –type (+/+)
Lane 2: Affected (EB/EB)
Lane 3: Carrier (EB/+)

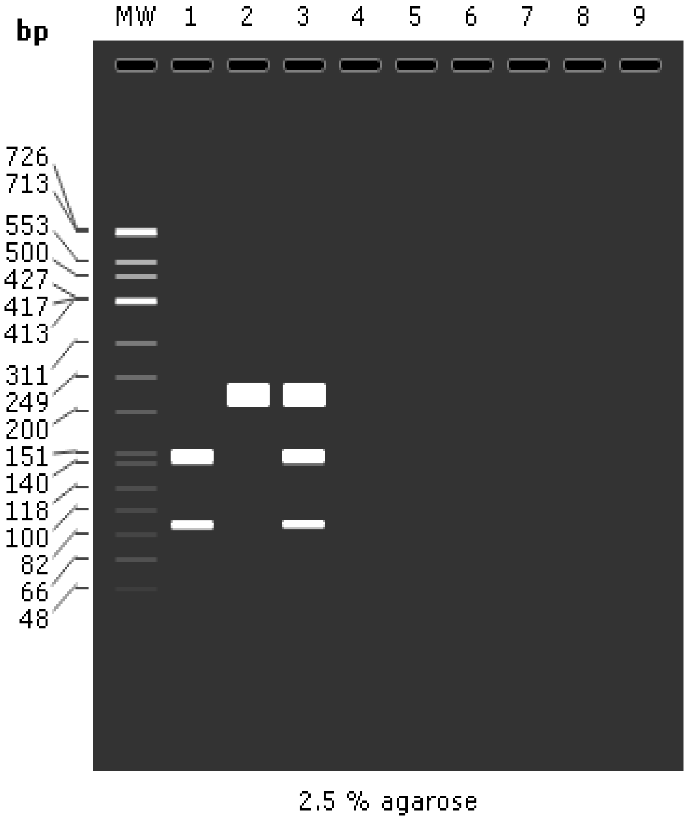

Fragment size:
239 bp: undigested
153 bp + 90 bp (restriction fragments)

AcyI digestion

MW: ϕX174DNA – *HinfI* Digest
Lane 1: Wild –type (+/+)
Lane 2: Affected (EB/EB)
Lane 3: Carrier (EB/+)

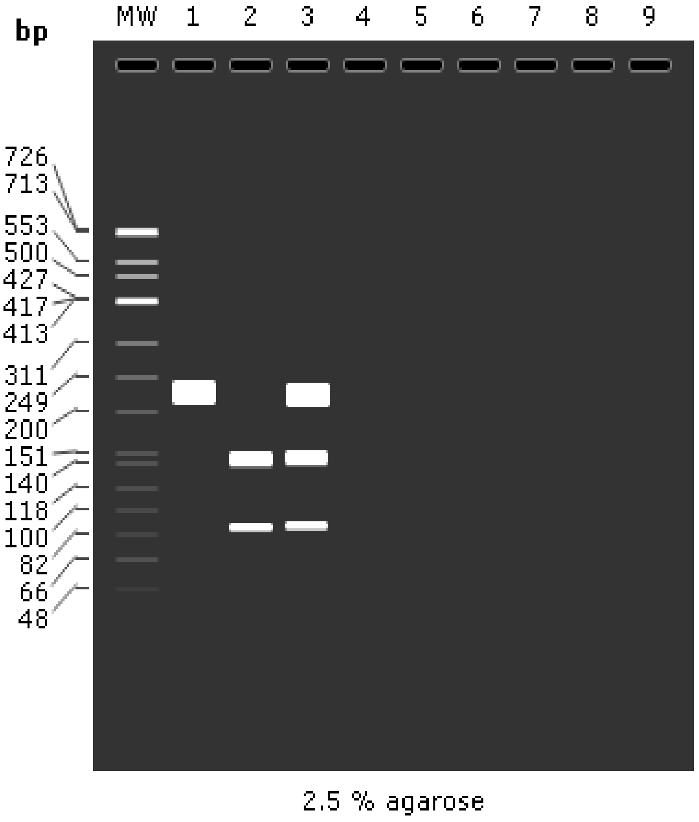

Fragment size:
243 bp: undigested
89 bp + 150 bp (restriction fragments)
